# Histone Lactylation Antagonizes Senescence and Skeletal Muscle Aging by Modulating Aging‐Related Pathways

**DOI:** 10.1002/advs.202412747

**Published:** 2025-05-19

**Authors:** Fanju Meng, Jianuo He, Xuebin Zhang, Wencong Lyu, Ran Wei, Shiyi Wang, Zhehao Du, Haochen Wang, Jinlong Bi, Xueyang Hua, Chao Zhang, Yiting Guan, Guoliang Lyu, Xiao‐Li Tian, Lijun Zhang, Wenbing Xie, Wei Tao

**Affiliations:** ^1^ The State Key Laboratory of Membrane Biology School of Life Sciences Peking University Beijing 100871 China; ^2^ Hefei National Laboratory for Physical Sciences at the Microscale School of Basic Medical Sciences Division of Life Sciences and Medicine University of Science and Technology of China Hefei 230026 China; ^3^ Department of Human Population Genetics Human Aging Research Institute (HARI) and School of Life Sciences Nanchang University Nanchang 330031 China; ^4^ Kunming Institute of Zoology Chinese Academy of Sciences Kunming Yunnan China; ^5^ Zhanjiang Institute of Clinical Medicine Zhanjiang Central Hospital Guangdong Medical University Zhanjiang 524045 China

**Keywords:** epigenetics, histone lactylation, senescence, skeletal muscle aging

## Abstract

Epigenetic alterations are among the prominent drivers of cellular senescence and/or aging, intricately orchestrating gene expression programs during these processes. This study shows that histone lactylation, plays a pivotal role in counteracting senescence and mitigating dysfunctions of skeletal muscle in aged mice. Mechanistically, histone lactylation and lactyl‐CoA levels markedly decrease during cellular senescence but are restored under hypoxic conditions primarily due to elevated glycolytic activity. The enrichment of histone lactylation at promoters is essential for sustaining the expression of genes involved in the cell cycle and DNA repair pathways. Furthermore, the modulation of enzymes crucial for histone lactylation, leads to reduced histone lactylation and accelerated cellular senescence. Consistently, the suppression of glycolysis and the depletion of histone lactylation are also observed during skeletal muscle aging. Modulating the enzymes can also lead to the loss of histone lactylation in skeletal muscle, downregulating DNA repair and proteostasis pathways and accelerating muscle aging. Running exercise increases histone lactylation, which in turn upregulate key genes in the DNA repair and proteostasis pathways. This study highlights the significant roles of histone lactylation in modulating cellular senescence as well as muscle aging, providing a promising avenue for antiaging intervention via metabolic manipulation.

## Graphical abstract

This study reveals histone lactylation as a metabolic‐epigenetic regulator countering cellular senescence and skeletal muscle aging. Reduced lactylation impairs cell cycle, DNA repair and proteostasis pathways, while exercise restores glycolytic activity and lactylation levels, preserving muscle function. The findings position histone lactylation as a novel senescence biomarker and highlight lactate metabolism modulation as a promising antiaging strategy.

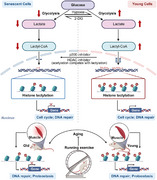

## 1 Introduction

Senescence is characterized by permanent cell cycle arrest along with increased expression of cyclin‐dependent kinase inhibitors and senescence‐associated secretory phenotypes (SASPs).^[^ ^1^ ^]^ Triggered by various extrinsic and intrinsic factors, including developmental signals, senescence plays crucial roles in a range of physiological and pathological processes.^[^ ^1^, ^2^ ^]^ Epigenetic alterations are one of the hallmarks and drivers of senescence and/or aging.^[^ ^3^, ^4^ ^]^ In particular, changes in histone modifications are pivotal to senescence progression via their functions in modulating the gene expression network, organizing chromatin architecture and maintaining genome stability.^[^ ^5^, ^6^, ^7^, ^8^, ^9^ ^]^ A plethora of studies have substantiated the promising applications of small molecules that target epigenetic modifiers in lifespan expansion and aging intervention.^[^ ^10^, ^11^, ^12^, ^13^ ^]^

Notably, senescent cells or aged tissues still retain high metabolic activity, and the cellular metabolic state is closely intertwined with epigenetic modulation of gene expression.^[^ ^14^ ^]^ Metabolic intermediates, such as acetyl‐CoA synthesized in the mitochondria, are critical in providing acetyl groups for posttranslational modifications (PTMs) of both nonhistone proteins and histones.^[^ ^15^ ^]^ These discoveries have greatly advanced our understanding of how histone codes that are influenced by cellular metabolic status contribute to the modulation of chromatin states, gene expression, and cell phenotypes.^[^ ^16^ ^]^

Histone lysine lactylation, a recently identified histone modification, has been shown to play a pivotal role in regulating gene expression and modulating cellular activities through the interplay between metabolism and epigenetics. For example, histone lactylation is involved in processes such as the immune response in macrophages and the development of cancer.^[^ ^17^ ^]^ Although not in great detail, histone lactylation has also been reported to be associated with other biological processes, such as tissue repair, cell proliferation, cell metabolism, neurodevelopment, and embryonic development.^[^ ^17^, ^18^, ^19^, ^20^, ^21^, ^22^, ^23^, ^24^, ^25^, ^26^ ^]^ These observations not only reveal the broad physiological functions of histone lactylation but also prove that the lactate produced from glycolysis is not only a byproduct of metabolism but also, by providing lactyl groups for histone lactylation, acts as a key regulator of cell fate. Indeed, the level of histone lactylation is closely related to metabolic kinetics in response to cellular activities as well as glycolytic enzymes, such as acyltransferase (p300) and histone deacetylase 1/3 (HDAC1/3), which may contribute to writing or erasing histone lactylation with their respective cofactors.^[^ ^17^, ^27^, ^28^ ^]^ Although histone lactylation has been shown to reside in regulatory elements and modulate the expression of genes involved in various biological processes,^[^ ^17^, ^21^, ^29^ ^]^ its roles and mechanisms in regulating cellular senescence and tissue functions during aging remain elusive.

In this study, we show that the level of histone lactylation markedly decreases during replicative senescence and tissue aging. In addition, hypoxia, which is accompanied by an increased level of histone lactylation, delays cellular senescence. Promoter enrichments of histone lactylation activate the cell cycle and DNA repair‐related genes to counteract cellular senescence and enhance DNA repair and proteostasis to antagonize muscle aging. Furthermore, the modulation of lactylation enzymes results in reduced histone lactylation, consequently accelerating senescence and muscle aging. Finally, after running exercise, the level of glycolysis in mouse skeletal muscle is increased and muscle dysfunction is mitigated, primarily owing to the increased abundance of histone lactylation, which reprograms genes and pathways linked to DNA repair and proteostasis. Thus, our data reveal that histone lactylation modulates cell cycle‐, DNA repair‐ and proteostasis‐related genes to antagonize cellular senescence and tissue aging. These findings suggest that histone lactylation may serve as a candidate marker to assess senescence/aging and provide new therapeutic avenues for combating tissue aging and extending the healthy lifespan.

## 2 Results

### 2.1 Histone Lactylation Dramatically Decreases During Senescence

**Figure 1:**
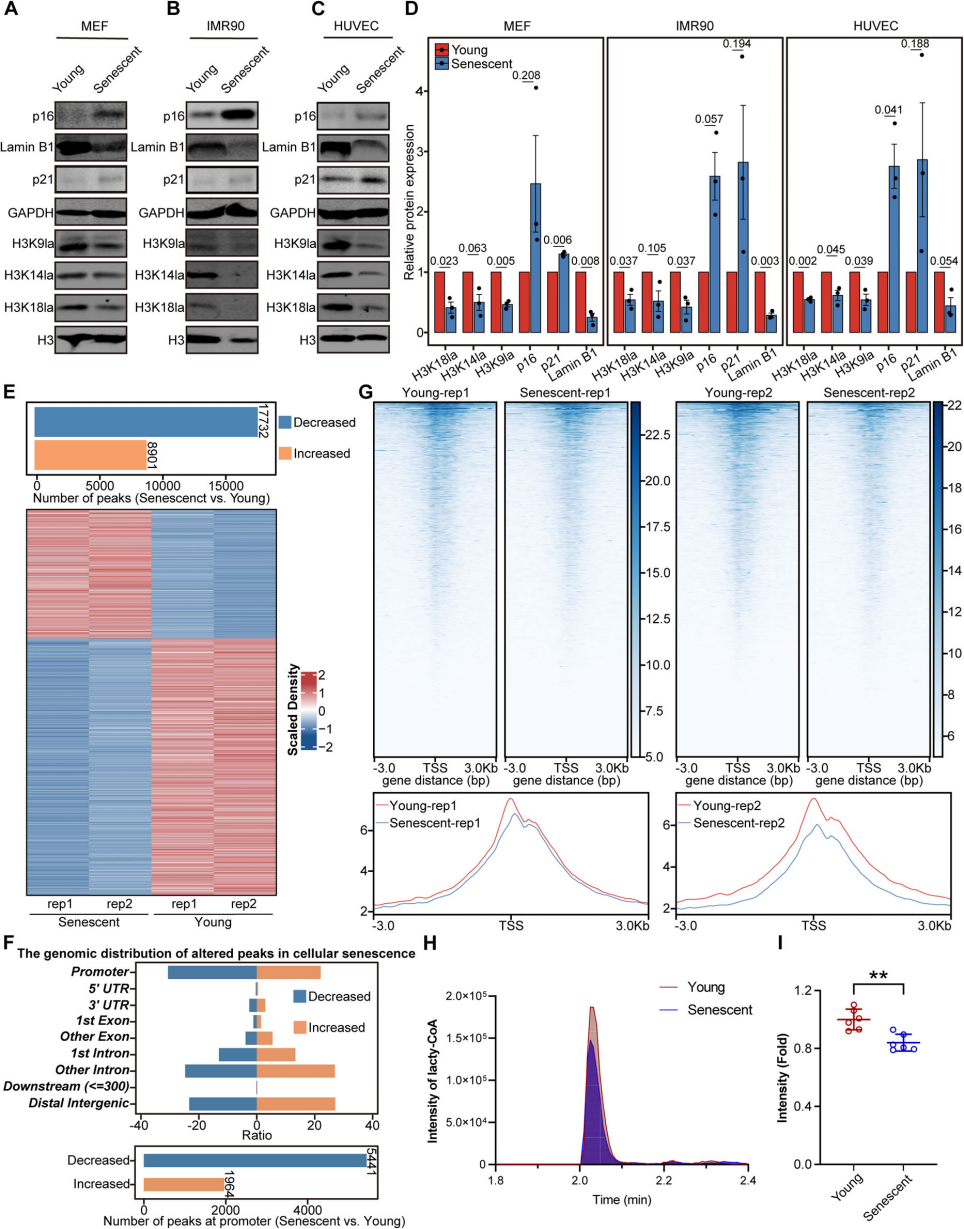
Histone lactylation decreases during cellular senescence. A–C) Immunoblotting of Lamin B1, p16, p21, H3K9la, H3K14la, and H3K18la in young and senescent MEFs (A), IMR90 cells (B), and HUVECs (C). GAPDH and H3 served as loading controls. D) Relative band intensity of immunoblots in A, B and C, *n* = 3. E) Top, number of altered peaks associated with cellular senescence; bottom, heatmap of altered H3K9la peaks associated with cellular senescence. F) Distribution of the altered H3K9la peaks in the genomic elements of IMR90 cells during senescence. G) Heatmaps and intensity profiles of H3K9la around ±3 kb of TSS throughout the genome in young and senescent IMR90 cells. H) Intensity of lactyl‐CoA in young and senescent IMR90 cells. I) Relative lactyl‐CoA intensity fold change in H, *n* = 6. The error bars represent the S.D. of independent experiments. Two‐tailed, unpaired Student's *t* tests were performed. **P < 0.01. TSS, transcription start site.

We utilized three replicative senescence models, i.e., human embryonic lung fibroblasts (IMR90 cells), mouse fibroblasts (MEFs), and human umbilical vein endothelial cells (HUVECs), to investigate how histone lactylation may be correlated with cellular senescence. At early passages, cells grow rapidly and are in a young state; subsequently, cells gradually cease to proliferate, which is referred to as replicative senescence. We used multiple markers to confirm the senescence state: p16/p21 expression and the percentage of cells with SA‐β‐gal^+^ staining significantly increased (**Figure** **1**A–D and Figure S1A–C, Supporting Information), whereas Lamin B1 and Ki‐67 expression decreased (Figure 1A–D and Figure S1D–F, Supporting Information) in all three senescent cell lines. Most importantly, the levels of histone lactylation at different H3 sites, including H3K9la, H3K14la, and H3K18la, dramatically decreased in all three senescent cell lines (Figure 1A–D), implying that senescence‐associated reductions in histone lactylation occur regardless of the cell type or species.

Next, we performed CUT&Tag for H3K9la in IMR90 cells to dissect the genomic regions that lose histone lactylation during senescence. As expected, the total number of decreased H3K9la peaks across the whole genome in senescent cells was significantly greater than that of increased H3K9la peaks (Figure 1E). More importantly, the decreased H3K9la peaks during senescence were enriched mainly at gene promoters, whereas the increased H3K9la peaks were distributed mainly in introns and distal intergenic regions (Figure 1F). In particular, decreases in the histone lactylation signal were pronounced within ±3 kb around the transcription start site (TSS) (Figure 1G). Because lactyl‐CoA is the most direct substrate for histone lactylation,^[^ ^17^ ^]^ we utilized mass spectrometry to measure the levels of lactyl‐CoA in both young and senescent cells. A significant decrease in lactyl‐CoA was associated with senescence (Figure 1H,I), indicating that the global reduction in histone lactylation during replicative senescence may be due to a lack of lactyl‐CoA in senescent cells. Thus, when exogenous sodium lactate (NALA) was applied to MEFs, HUVECs, and IMR90 cells, consistent with previous studies,^[^ ^17^, ^22^ ^]^ the histone lactylation level of the treated cells are increased and the percentage of the treated cells that underwent senescence significantly decreased (Figure S2, Supporting Information), suggesting that histone lactylation may function to prevent cellular senescence.

### 2.2 Glycolysis Inhibition Leads to a Loss of Histone Lactylation During Senescence

**Figure 2:**
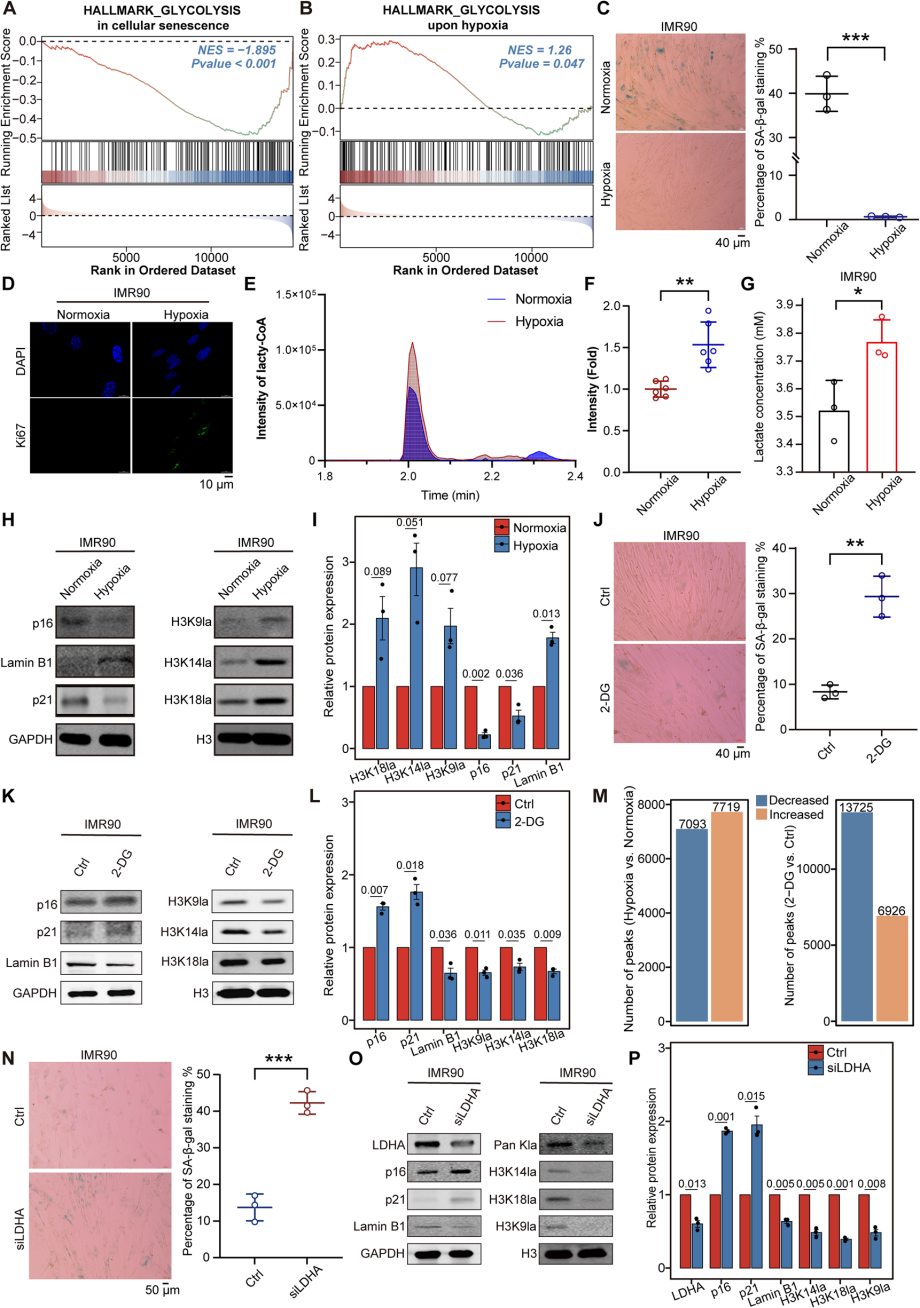
Glycolysis inhibition leads to a loss of histone lactylation and triggers cellular senescence. A) GSEA of glycolysis genes in IMR90 cells during senescence. B) GSEA of glycolysis genes in IMR90 cells upon hypoxia exposure. C) SA‐β‐gal staining of IMR90 cells cultured under normoxia and hypoxia. The percentages of SA‐β‐gal^+^ cells are shown on the right, *n* = 3. D) Immunofluorescence of Ki67 and DAPI in IMR90 cells cultured under normoxia and hypoxia. E) Intensity of lactyl‐CoA in normoxic and hypoxic IMR90 cells. F) Relative lactyl‐CoA intensity fold change in E, *n* = 6. G) Intracellular lactate levels in IMR90 cells cultured under normoxia and hypoxia, *n* = 3. H) Protein levels of p16, Lamin B1, p21, H3K9la, H3K14la, and H3K18la in IMR90 cells cultured under normoxia and hypoxia. GAPDH and H3 served as the loading controls. I) Relative band intensity of immunoblots in H, *n* = 3. J) SA‐β‐gal staining of IMR90 cells with or without 2‐DG treatment in a hypoxic environment. The percentages of SA‐β‐gal^+^ cells are shown on the right, *n* = 3. K) Immunoblotting of Lamin B1, p16, p21, H3K9la, H3K14la, and H3K18la in IMR90 cells cultured under hypoxia with and without 2‐DG treatment. GAPDH and H3 served as loading controls. L) Relative band intensity of immunoblots in K, *n* = 3. M) The number of altered peaks upon hypoxia or 2‐DG treatment. N) SA‐β‐gal staining of IMR90 cells in the presence or absence of *Ldha* siRNA. The percentages of SA‐β‐gal^+^ cells are shown on the right, *n* =3. O) Immunoblotting of LDHA, Pan Kla, H3K18la, H3K14la, H3K9la, Lamin B1, p16, and p21 in IMR90 cells in the presence or absence of *Ldha* siRNA. H3 and GAPDH served as the loading controls. P) Relative band intensity of immunoblots in O, *n* = 3. The error bars represent the S.D. of independent experiments. Two‐tailed, unpaired Student's *t* tests were performed. *P < 0.05, **P < 0.01, ***P < 0.001. Normoxia, 20% O_2_; hypoxia, 3% O_2_.

Considering that exogenous lactate treatment can increase the level of histone lactylation and further inhibit cellular senescence, we hypothesize that active intervention in the endogenous lactate level in cells is sufficient to modulate the abundance of histone lactylation and impact senescence progression. Hypoxia can induce glycolysis and produce large amounts of endogenous lactate,^[^ ^30^ ^]^ and previous studies have shown that hypoxia considerably extends the cell lifespan and that upregulation of glycolysis can resist senescence;^[^ ^31^ ^]^ therefore, we established a physiological hypoxic (3% O_2_) environment in IMR90 and MEF cells to test the aforementioned hypothesis. As expected, a decrease in glycolysis levels in senescent IMR90 cells was observed (**Figure** **2**A), while hypoxia treatment led to a significant increase in glycolysis levels (Figure 2B and Figure S3A, Supporting Information). Thus, under normoxic conditions, IMR90 cells rapidly became senescent, and their proliferative capacity was very poor. However, in the hypoxic environment, the proliferative capacity of senescent IMR90 cells was elevated, and the progression of senescence was significantly delayed (Figure 2C,D). The same phenomenon was also observed in MEFs (Figure S4A, Supporting Information). In addition, both lactyl‐CoA and lactate levels were significantly greater in IMR90 cells under hypoxic conditions than in those under normoxic conditions (Figure 2E–G). Consistently, hypoxia treatment markedly increased histone lactylation, including H3K9la, H3K14la and H3K18la, in IMR90 and MEF cells (Figure 2H,I and Figure S4B, Supporting Information).

To test whether hypoxia affects histone lactylation via the glycolysis‐lactate/lactyl‐CoA axis, we subjected IMR90 cells to hypoxic conditions and treated these cells with the glycolysis inhibitor 2‐deoxy‐d‐glucose (2‐DG). Our results revealed that 2‐DG treatment significantly inhibited glycolysis, and more importantly, the ability of hypoxia to mitigate senescence was abolished in IMR90 cells treated with 2‐DG (Figure 2J–L and Figure S3B, Supporting Information). In addition, the abundance of histone lactylation was primarily increased across the whole genome upon hypoxia exposure and decreased upon 2‐DG treatment (Figure 2M and Figure S5, Supporting Information). These results indicate that hypoxia restores histone lactylation by enhancing intracellular glycolytic flux.

LDHA is a critical enzyme that converts pyruvate to lactate and plays a pivotal role in glycolysis.^[^ ^32^ ^]^ The expression of LDHA was downregulated during senescence and upregulated following hypoxia treatment (Figure S6A, Supporting Information). To further confirm that the glycolysis‐lactate/lactyl‐CoA axis directly influences histone lactylation levels to impact cellular senescence, we knocked down the LDHA gene in IMR90 cells, and depletion of LDHA resulted in decreased levels of lactate as well as histone lactylation (Figure 2O and Figure S6B, Supporting Information). More importantly, knockdown of LDHA accelerated IMR90 cell senescence, as manifested by increased SA‐β‐gal staining, elevated expression of p21 and p16, and decreased Lamin B1 expression (Figure 2N–P). Similarly, LDHA inhibitors such as galloflavin and oxamate also reduced lactate levels in IMR90 cells (Figure S6C, Supporting Information), thereby reducing histone lactylation levels and promoting cellular senescence (Figure S6D–G, Supporting Information). Additionally, LDHA inhibitors can induce senescence in MEFs (Figure S6H, Supporting Information). Therefore, glycolysis inhibition and the resulting deficiency in lactate/lactyl‐CoA during cellular senescence are the primary contributors to the senescence‐associated loss of histone lactylation, and modulation of the metabolic environment may be an effective strategy to intervene in cellular senescence.

Besides, the addition of NALA to 2‐DG‐treated or siLDHA IMR90 cells indeed restored histone lactylation levels and alleviated cellular senescence phenotype (Figure S7A–D, Supporting Information). However, for IMR90 cells cultured under hypoxic condition, the addition of NALA did not further increase the histone lactylation levels (Figure S7E, Supporting Information). Moreover, the knockdown of LDHA effectively inhibited the hypoxia‐induced increase in histone lactylation and reversed the delayed senescence that occurs under hypoxic conditions (Figure S7F,G, Supporting Information). Therefore, modulating lactate levels can alter histone lactylation, thereby influencing cellular senescence.

### 2.3 Histone Lactylation Modulates Cell Cycle‐ and DNA Repair‐Related Genes to Antagonize Cellular Senescence

**Figure 3:**
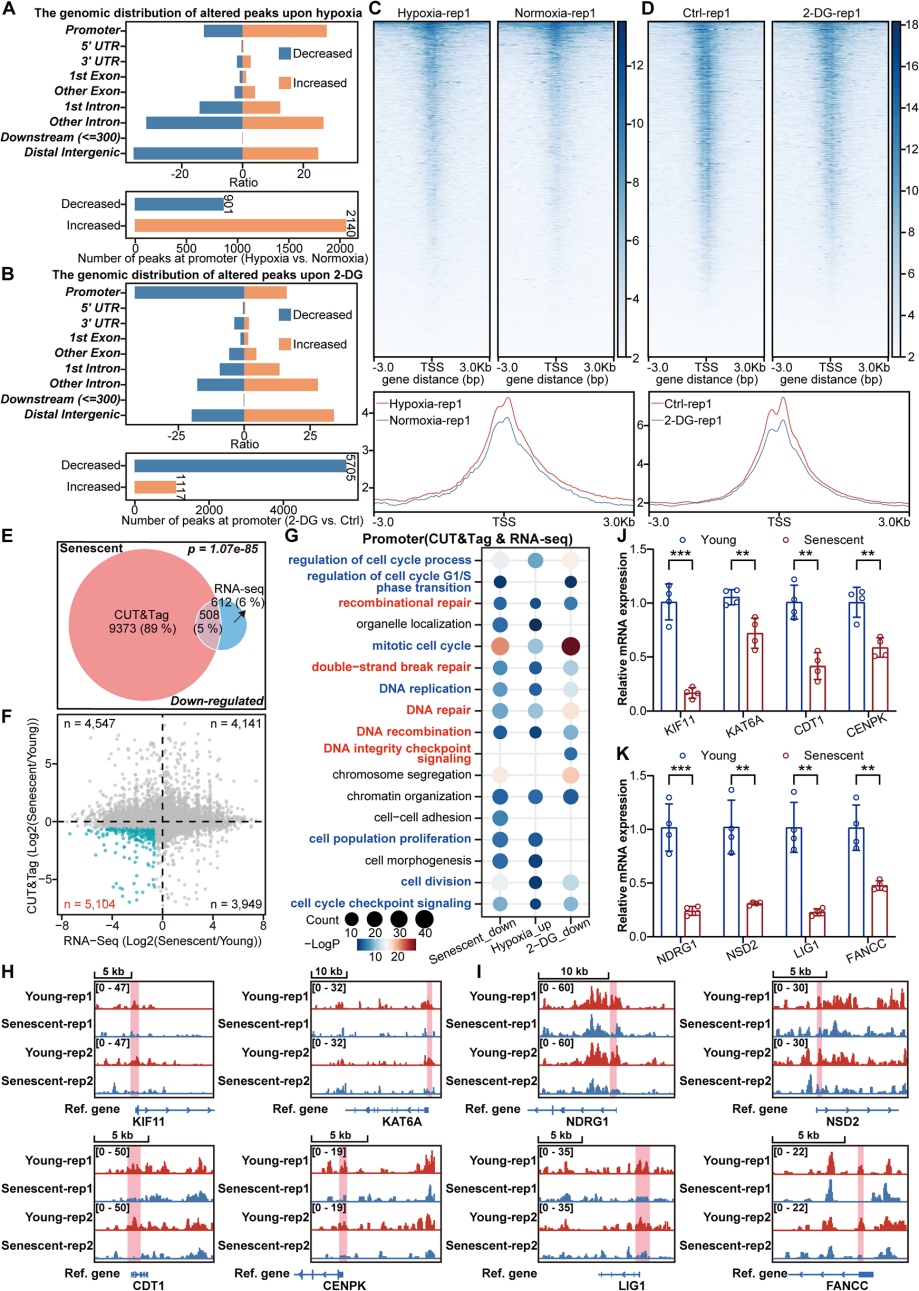
Histone lactylation modulates the expression of genes related to the cell cycle and DNA repair. A) Distribution of the altered H3K9la peaks in genomic elements in IMR90 cells upon hypoxia exposure. B) Distribution of the altered H3K9la peaks in genomic elements in IMR90 cells treated with 2‐DG in a hypoxic environment. C) Heatmaps and intensity profiles of H3K9la around ± 3 kb of TSS throughout the genome in IMR90 cells cultured under hypoxia or normoxia. D) Heatmaps and intensity profiles of H3K9la around ± 3 kb of TSS throughout the genome with or without 2‐DG treatment in IMR90 cells cultured in a hypoxic environment. E) Venn diagram showing the overlapping genes between decreased H3K9la peak‐associated genes identified via CUT&Tag and downregulated genes identified via RNA‐seq data related to cellular senescence. F) Scatter plot showing the relationships between genes with alterations in promoter histone lactylation and those with changes in gene expression during cellular senescence. The green dots indicate the genes that lost promoter histone lactylation and in which senescence was downregulated. G) Gene Ontology pathway analysis of genes with altered H3K9la peaks at their promoters and corresponding downregulation during senescence, upregulation upon hypoxia, or downregulation upon 2‐DG treatment in IMR90 cells. H) Snapshots of H3K9la peaks at the promoters of *KIF11*, *KAT6A*, *CDT1*, and *CENPK* in young and senescent IMR90 cells. I) Snapshots of H3K9la peaks at the promoters of *NDRG1*, *NSD2*, *LIG1*, and *FANCC* in young and senescent IMR90 cells. J) mRNA levels of the cell cycle‐related genes *KIF11*, *KAT6A*, *CDT1*, and *CENPK* in young and senescent IMR90 cells. The cycle threshold (Ct) values of these genes were normalized to that of *ACTB*, *n* = 4. K) mRNA levels of the DNA repair‐related genes *NDRG1*, *NSD2*, *LIG1*, and *FANCC* in young and senescent IMR90 cells. The cycle threshold (Ct) values of these genes were normalized to that of *ACTB*, *n* = 4. The error bars represent the S.D. of independent experiments. Two‐tailed, unpaired Student's *t* tests were performed. **P < 0.01, ***P < 0.001. Normoxia, 20% O_2_; hypoxia, 3% O_2_; TSS, transcription start site.

We aimed to elucidate how alterations in histone lactylation during senescence may impact senescence progression. Since histone lactylation levels dramatically decrease during cell senescence and the decreased H3K9la peaks during senescence are enriched mainly at gene promoters, we also performed CUT&Tag experiments for H3K9la in hypoxia‐ or 2‐DG‐treated IMR90 cells. As expected, hypoxic conditions mainly stimulated histone lactylation in IMR90 cells, whereas 2‐DG treatment reduced the global level of histone lactylation. More importantly, the increased H3K9la peaks upon hypoxia exposure mostly resided in promoter regions, whereas the decreased H3K9la peaks were primarily distributed in introns and distal intergenic regions (**Figure** **3**A,C and Figure S8A, Supporting Information). In contrast, the H3K9la peaks whose expression decreased upon 2‐DG treatment were also enriched mainly at promoter regions, whereas the H3K9la peaks whose expression increased were largely distributed in introns and distal intergenic regions (Figure 3B,D and Figure S8B, Supporting Information). Our analysis also identified a significant overlap of genes among three experimental conditions: those with decreased H3K9la peaks during cellular senescence, those with increased H3K9la peaks under hypoxic conditions, and those with decreased H3K9la peaks following 2‐DG treatment (Figure S9, Supporting Information). These results suggest that promoter regions may be the key genomic regions through which histone lactylation impacts the progression of senescence. This finding is in line with a previous study showing that increased histone lactylation activates the promoters of homeostatic genes in the late phase of M1 macrophage polarization.^[^ ^17^ ^]^

Hence, we conducted intersection analyses between CUT&Tag data and RNA‐seq data to determine which groups of genes or pathways affect histone lactylation. We compared genes associated with decreased H3K9la peaks in cellular senescence or 2‐DG treatment with their corresponding downregulated genes, and we compared genes associated with increased H3K9la peaks upon hypoxia exposure with their respective upregulated genes. Unsurprisingly, the intersecting gene numbers between the two datasets in all three conditions were significant (Figure 3E and Figure S10, Supporting Information). Additionally, more genes lost histone lactylation at their promoters and were downregulated with increasing cellular senescence (Figure 3F). In all conditions, altered peaks exhibited a significantly positive correlation with the corresponding differentially expressed genes (DEGs) (Figure S11A–F, Supporting Information). Moreover, genome‐wide analysis revealed a significantly positive correlation between all detected CUT&Tag peaks and their corresponding expressed genes across the three conditions (Figure S11G–L, Supporting Information). Intriguingly, genes involved in pathways such as the mitotic cell cycle, DNA replication, cell population proliferation, double‐strand break repair and recombinational repair lost histone lactylation at their promoters and were downregulated during senescence, but their promoters were lactylated and activated in a hypoxic environment (Figure 3G). These cell cycle‐ and DNA repair‐related genes were delactylated at promoters and inhibited upon 2‐DG treatment (Figure 3G). We present here several key cell cycle‐related genes, such as *KIF11*, *KAT6A*, *CDT1*, and *CENPK* ^[^ ^33^, ^34^, ^35^, ^36^ ^]^ (Figure 3H,J), and DNA repair‐related genes, such as *NDRG1*, *NSD2*, *LIG1*, and *FANCC* ^[^ ^37^, ^38^, ^39^, ^40^ ^]^ (Figure 3I,K), as well as other cell cycle‐ and DNA repair‐related genes whose promoters are delactylated and downregulated during cellular senescence (Figure S12, Supporting Information).

To further substantiate the cause‐effect relationship between histone lactylation and the expression of cell cycle and DNA repair‐associated genes, we show that, on a per‐gene basis, the levels of both promoter histone lactylation and the expression of cell cycle‐ and DNA repair‐related genes were concurrently elevated in response to hypoxic condition (Figure S13A,B,E, Supporting Information). In contrast, under 2‐DG treatment, the histone lactylation and expression of cell cycle‐ and DNA repair‐ related genes were both downregulated (Figure S13C,D,F, Supporting Information). In our further set of experiments, we added NALA to 2‐DG‐treated IMR90 cells or knocked down LDHA in IMR90 cells under hypoxic condition to investigate whether perturbing lactylation levels would impact the expression of cell cycle and DNA repair‐related genes. We found that cell cycle and DNA repair‐related genes get delactylated and downregulated under 2‐DG treatment, but supplementation with NALA restores both histone lactylation levels and gene expression of the above genes (Figure S14A,B, Supporting Information). Conversely, cell cycle and DNA repair‐related genes get their promoters lactylated and upregulated upon hypoxia exposure, but knockdown of LDHA in these cells could reverse these changes (Figure S14C,D, Supporting Information). Thus, these results collectively highlight the critical role of histone lactylation in modulating gene expression of cell cycle and DNA repair‐related genes under different metabolic conditions.

Next, we treated senescent IMR90 cells with exogenous pyruvate and observed elevated histone lactylation levels and reduced cellular senescence (Figure S15, Supporting Information). Notably, LDHA knockdown blocked both the pyruvate‐induced increase in histone lactylation and the reduction in senescence (Figure S15, Supporting Information). Subsequent H3K9la CUT&Tag analysis, in which we compared the pyruvate‐treated group with the control group, verified a marked elevation in histone lactylation, particularly within promoter regions (Figure S16A–C, Supporting Information). Moreover, we found that genes associated with cell cycle and DNA repair pathways exhibited enhanced histone lactylation at their promoters and were upregulated upon pyruvate treatment (Figure S16D–G, Supporting Information). These findings again demonstrate that histone lactylation antagonizes the progression of senescence primarily by modulating genes related to DNA repair and cell cycle.

### 2.4 Inhibiting Lactyltransferase p300 or Delactylase HDAC Reduces Histone Lactylation and Accelerates Cellular Senescence

Previous research has shown that the lactyl groups involved in histone lactylation modifications are directly supplied by l‐lactyl CoA and are catalyzed by p300.^[^ ^17^ ^]^ Additionally, class I HDACs (HDAC1‐3) are the most effective “erasers” of lysine lactylation modifications in vitro.^[^ ^41^ ^]^ We aimed to investigate how these epigenetic modifiers of histone lactylation may impact senescence progression.

**Figure 4:**
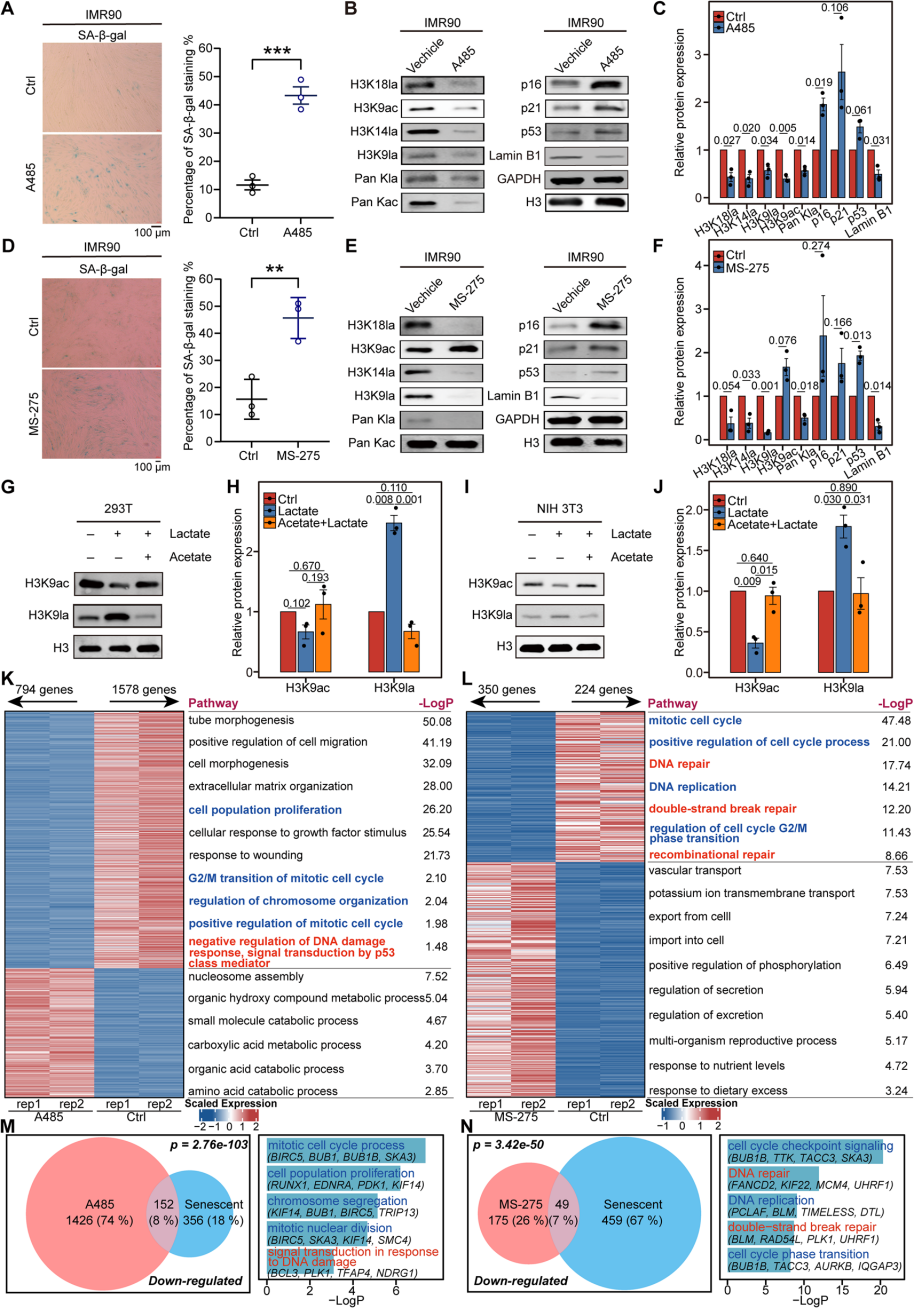
Inhibiting lactyltransferase p300 or delactylase HDAC decreases histone lactylation and accelerates cellular senescence. A) SA‐β‐gal staining of IMR90 cells in the presence or absence of A485 (10 µM). The percentages of SA‐β‐gal^+^ cells are shown on the right, *n* = 3. B) Immunoblotting of H3K18la, H3K14la, H3K9la, H3K9ac, Pan Kla, Pan Kac, Lamin B1, p16, p21, and p53 in IMR90 cells in the presence or absence of A485. H3 and GAPDH served as the loading controls. C) Relative band intensity of the immunoblots in B, *n* = 3. D) SA‐β‐gal staining of IMR90 cells in the presence or absence of MS‐275. The percentages of SA‐β‐gal^+^ cells are shown on the right, *n* = 3. E) Immunoblotting of H3K18la, H3K14la, H3K9la, H3K9ac, Pan Kla, Pan Kac, Lamin B1, p16, p21, and p53 in IMR90 cells in the presence or absence of MS‐275 (10 µM). H3 and GAPDH served as the loading controls. F) Relative band intensity of the immunoblots in E, *n* = 3. G) Immunoblotting of H3K9la and H3K9ac in 293T cells in the presence or absence of lactate (20 mM) or lactate (20 mM) plus acetate (20 mM). H) Relative band intensity of immunoblots in G, *n* = 3. I) Immunoblotting of H3K9la and H3K9ac in NIH‐3T3 cells in the presence or absence of lactate and lactate plus acetate. J) Relative band intensity of immunoblots in I, *n* = 3. K) Heatmap showing the expression levels of altered genes and Gene Ontology pathway analysis of these DEGs in IMR90 cells treated with A485. L) Heatmap showing the expression levels of altered genes and Gene Ontology pathway analysis of these DEGs in IMR90 cells treated with MS‐275. M) Venn diagram showing the overlapping genes between genes downregulated upon A485 treatment and those with decreased histone lactylation and downregulation during senescence. Bar plot showing the Gene Ontology pathway analysis of the overlapping genes. N) Venn diagram showing the overlapping genes between genes downregulated upon MS‐275 treatment and those with decreased histone lactylation and downregulation during senescence. Bar plot showing the Gene Ontology pathway analysis of the overlapping genes. The error bars represent the S.D. of independent experiments. Two‐tailed, unpaired Student's *t* tests were performed. **P < 0.01, ***P < 0.001.

The introduction of the p300 inhibitor A485^[^ ^42^ ^]^ into IMR90 cells effectively led to increased SA‐β‐gal^+^ signals (**Figure** **4**A) and accelerated cellular senescence (Figure 4B,C). Notably, A485 suppressed histone lactylation at multiple histone sites, including H3K9la, H3K14la, and H3K18la (Figure 4B,C). Intriguingly, the introduction of A485 also resulted in a decrease in histone acetylation levels (Figure 4B,C). We subsequently introduced MS‐275 to inhibit class I HDAC^[^ ^43^ ^]^ in IMR90 cells, and surprisingly, MS‐275 also effectively decreased histone lactylation levels at multiple histone lysine sites and led to an increase in SA‐β‐gal^+^ signals and the acceleration of cellular senescence (Figure 4D–F). Notably, these effects were accompanied by an increase in histone acetylation levels (Figure 4E,F). In accordance with a recently published study,^[^ ^44^ ^]^ we hypothesized that a competitive relationship exists between histone lactylation and histone acetylation. Therefore, we separately introduced lactate and acetate into 293T or NIH‐3T3 cells, and the above two modifications indeed exhibited a competitive relationship in both cell types (Figure 4G–J).

Moreover, we conducted CUT&Tag experiments for H3K9la and H3K9ac in A485‐ and MS‐275‐ treated cells to dissect the alteration profiles for these two histone modifications in the above treatments. We found that histone lactylation was significantly decreased after the inhibition of both p300 and HDAC, while histone acetylation was decreased after p300 inhibition (A485) and increased after HDAC inhibition (MS‐275) (Figure S17, Supporting Information). Our analysis identified a significant overlap of genes between two experimental conditions: those with decreased H3K9la peaks under A485 treatment and those with decreased H3K9la peaks under MS‐275 treatment (Figure S18A, Supporting Information). And both treatment groups exhibited a significant positive correlation between histone lactylation and gene expression (Figure S18B,C, Supporting Information). Moreover, upon MS‐275 treatment, the cell cycle and DNA repair genes, despite showing slightly increased histone acetylation at their promoters, are associated with decreased histone lactylation and exhibit marked downregulation (Figure S19, Supporting Information). These results indicate that the downregulation of cell cycle and DNA repair pathways is specifically associated with decreased histone lactylation, not histone acetylation. In addition, we knocked down *p300* and *Hdac1* in IMR90 cells. Similarly, we found that histone lactylation was significantly decreased after the knockdown of both *p300* and *Hdac1*, while histone acetylation was decreased after *p300* knockdown and increased after *Hdac1* knockdown, with a similar competitive relationship between the two histone modifications (Figure S20, Supporting Information). Thus, perturbing both writers and erasers of histone lactylation led to the loss of histone lactylation and the acceleration of cellular senescence.

Next, we aimed to identify the genes and pathways disrupted by inhibiting the writers and erasers involved in histone lactylation. Bulk RNA‐seq of A485 treated‐treated IMR90 cells revealed a decreased expression of genes involved in the cell cycle and DNA repair‐related pathways, such as those involved in cell proliferation, positive regulation of the mitotic cell cycle, negative regulation of the DNA damage response, and the cellular response of growth factors to stimuli (Figure 4K). MS‐275 treatment also led to the downregulation of genes enriched in pathways related to the mitotic cell cycle, positive regulation of the cell cycle process, DNA repair, and DNA replication (Figure 4L). These results are similar to our previous observations in which the loss of histone lactylation during cellular senescence led to downregulation of cell cycle‐ and DNA repair related‐related genes and pathways.

Thus, we further aimed to determine whether the genes downregulated following A485 or MS‐275 treatment overlap with those inhibited by the loss of histone lactylation during cellular senescence. Indeed, we found a significant overlap of genes between senescence and inhibitor treatments, and these genes were enriched predominantly in pathways such as the cell cycle and DNA repair (Figure 4M,N and Figure S21, Supporting Information). In summary, histone lactylation loss, whether through the inhibition of histone modifiers or cellular senescence, reduces the expression of genes related to the cell cycle and DNA repair and promotes cellular senescence.

### 2.5 Glycolysis and Histone Lactylation Levels decrease During Skeletal Muscle Aging

**Figure 5:**
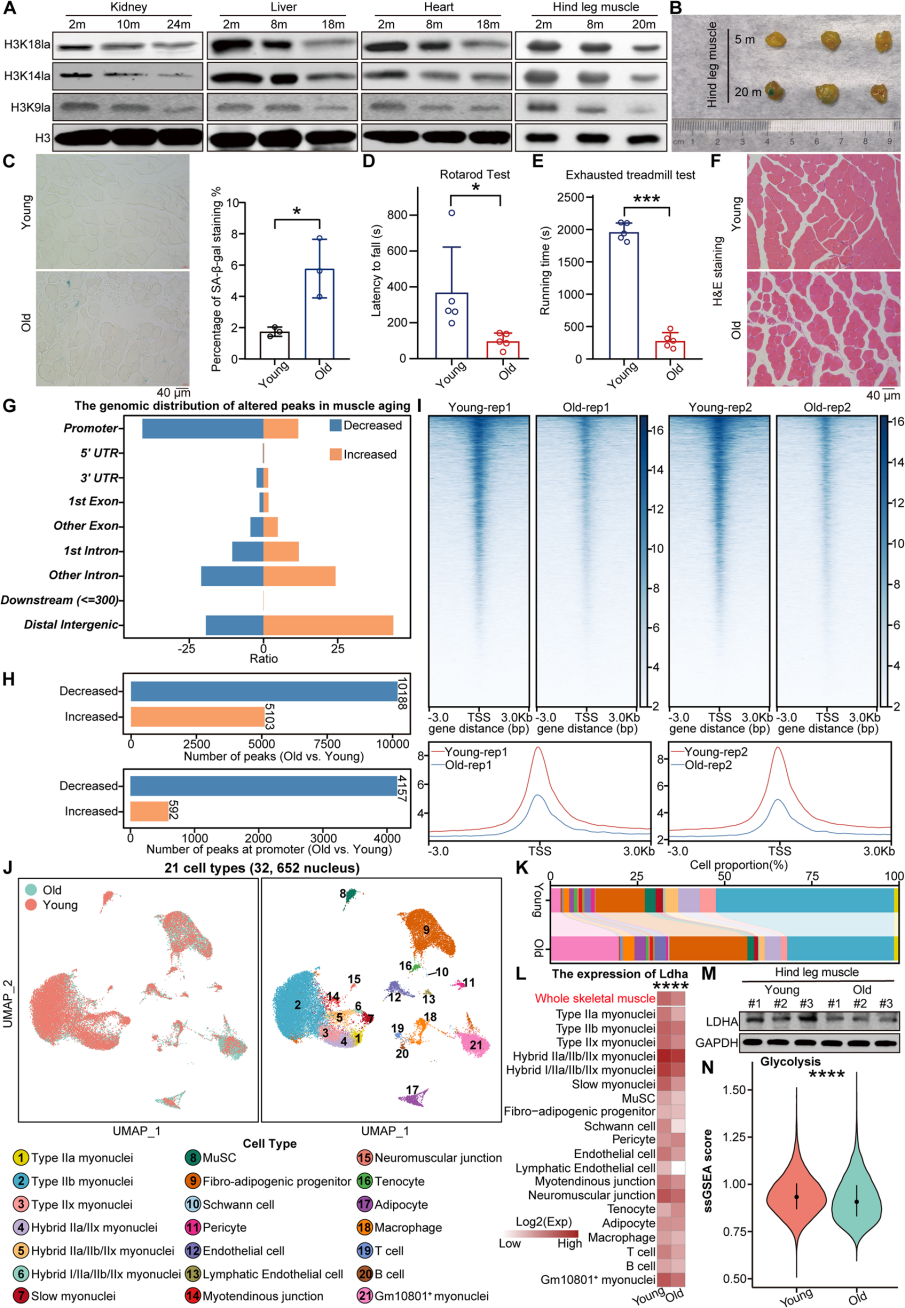
Glycolysis and histone lactylation levels decrease during skeletal muscle aging. A) Protein levels of H3K18la, H3K14la, and H3K9la in the kidney, liver, heart, and hindleg muscle of female mice. H3 served as the loading control. B) SA‐β‐gal staining of skeletal muscle from the gastrocnemius legs of young and old female mice. Three mice were measured in each group. C) SA‐β‐gal staining of skeletal muscle sections from the hindlegs of young and old female mice. Three mice were measured in each group. The percentage of SA‐β‐gal^+^ staining is shown on the right. D) Rotarod performance test for young and old male mice. The *y*‐axis represents the persistence time spent on the rotating rod (latency to fall). Five mice were tested in each group. E) Treadmill fatigue test for young and old male mice. The y‐axis represents the time taken to reach fatigue. Five mice were tested in each group. F) H&E staining of skeletal muscle from the hindlegs of young and old female mice. G) Distribution of the altered H3K9la peaks in genomic elements in muscle of young and old mice. H) The number of peaks at whole genome and peaks at promoter in muscle of young and old mice. I) Heatmaps and intensity profiles of H3K9la around ± 3 kb of TSS throughout the genome in muscle of young and old mice. J) UMAP visualization of the 21 major cell types in muscle of young and old mice. The left part shows the UMAP plot of the sample origin (*n*  =  2 per group). K) Stacked bar chart of the cell proportions in muscle of young and old mice. L) The mRNA expression level of *Ldha* in the whole skeletal muscle and each cell type of young and old mice. M) Protein levels of LDHA in the skeletal muscle of young and old mice. GAPDH served as the loading control. N) Alterations in the expression of glycolysis hallmark genes in skeletal muscle between young and old mice. The error bars represent the S.D. of independent experiments. Two‐tailed, unpaired Student's *t* tests were performed. *P < 0.05, ***P < 0.001, ****P < 0.001. TSS, transcription start site.

Given the difference between cellular senescence and the tissue aging of individuals, we further explored the dynamics of histone lactylation in the most metabolically active organs of mice during aging. Immunoblotting analysis revealed a significant reduction in histone lactylation levels in organs from aged mice, including the kidney, liver, heart, and skeletal muscle, indicating a pronounced decrease in these modifications with age (**Figure** **5**A).

Skeletal muscle is the primary motor organ. Therefore, we hypothesized that histone lactylation likely affects the exercise capacity of mice. We first evaluated the changes in the structure and motor ability of skeletal muscle during aging and found that fiber fractures accumulate and that SA‐β‐gal^+^ staining increases in aged mice (Figure 5B,C). Moreover, the exercise capacity of aged mice considerably decreased (Figure 5D,E). Our histological analysis revealed age‐related muscular alterations characterized by compromised tissue architecture, including widened intercellular spaces and rounded myocyte morphology. In addition, muscle atrophy became evident with obvious morphological changes during muscle aging (Figure 5F).

We performed H3K9la CUT&Tag experiment in gastrocnemius muscle from young (3‐month‐old) and old (20‐month‐old) mice. Consistent with previous findings, we observed a significant reduction in histone lactylation levels in the old skeletal muscle when compared to the young samples. Echoing our prior results in cellular senscence, this decrease of histone lactylation was predominantly concentrated at gene's promoters (Figure 5G–I). Next, to confirm that histone lactylation is functionally important for young skeletal muscle, we performed single‐nucleus RNA sequencing (snRNA‐seq) using young (3‐month‐old) and old (20‐month‐old) gastrocnemius muscle tissues to elucidate the changes in the cell landscape during skeletal muscle aging. After quality control, we acquired 32,652 nucleus and identified a total of 21 cell types: Type IIa myonuclei, Type IIb myonuclei, Type IIx myonuclei, Hybrid IIa/IIx myonuclei, Hybrid IIa/Ib/IIx myonuclei, Hybrid I/IIa/IIb/IIx myonuclei, Slow myonuclei, Muscle stem cell (MuSC), Fibro‐adipogenic progenitor, Schwann cell, Pericyte, Endothelial cell, Lymphatic endothelial cell, Myotendinous junction, Neuromuscular junction, Tenocyte, Adipocyte, Macrophage, T cell, B cell, and Gm10801^+^ myonuclei (Figure 5J and Figure S22A, Supporting Information).

We found that the proportion of Type II myonuclei was significantly decreased as well as MuSC during muscle aging, while adipocyte and immune cells (such as macrophage and T cell) exhibited obvious upward trends in old mice (Figure 5K). These changes align with the macroscopic transitions in skeletal muscle aging, including stem cell loss, muscle mass decline, fat accumulation, and increased immune response.^[^ ^45^, ^46^, ^47^, ^48^, ^49^ ^]^ Moreover, we identified that Type IIb myonuclei and Adipocyte exhibited the highest number of DEGs, with Type IIb myonuclei showing the most sensitive to aging, highlighting its profound transcriptomic changes during muscle aging (Figure S22B, Supporting Information). We found that the transcriptional noise of various cell types increases significantly during skeletal muscle aging, indicating the augmentation of transcription disorders and disturbance of gene expression (Figure S22C, Supporting Information). Consistent with the previous reports,^[^ ^45^, ^46^, ^47^ ^]^ our results also demonstrated that the activation of the SASP occurs during muscle aging, accompanied by the activation of the inflammatory response (Figure S23A,B, Supporting Information). Furthermore, autophagy was inhibited during muscle aging (Figure S23C, Supporting Information). We also assessed changes in aging‐related pathways and found that most cell types in skeletal muscle underwent senescence, with myonuclei exhibiting the most pronounced aging responses (Figure S24, Supporting Information).

Furthermore, considering the previously observed decrease in glycolytic levels and the resulting loss of histone lactylation during cellular senescence, we aimed to explore glycolytic alterations as well as the function of histone lactylation in muscle aging. The glycolytic capacity of old skeletal muscle was significantly decreased, as revealed by reduced expression of key glycolysis genes such as Ldha (Figure 5L–N). And these observations were also true for most cell types, implying that these cell types may also undergo alterations in histone lactylation during muscle aging. (Figure 5L).

### 2.6 Decreased Histone Lactylation in Aged Muscle Dampens DNA Repair and Proteostasis Pathways

**Figure 6:**
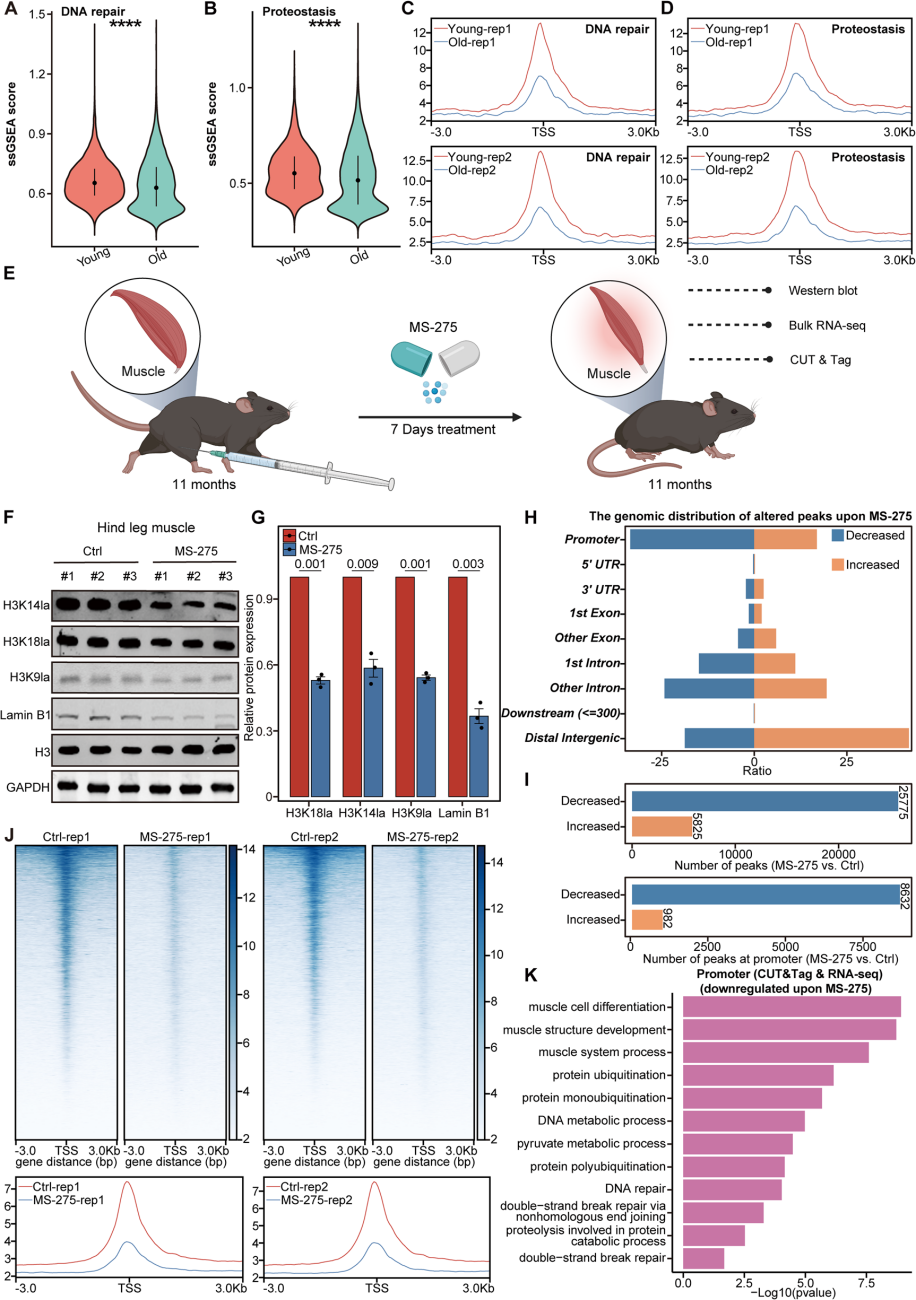
Decreased histone lactylation in aging muscle dampens DNA repair and proteostasis pathways. A) The expression of genes related to DNA repair in muscle of young and old mice. B) The expression of genes related to proteostasis in muscle of young and old mice. C) Intensity profiles of H3K9la around ± 3 kb of TSS on DNA repair pathway in muscle of young and old mice. D) Intensity profiles of H3K9la around ± 3 kb of TSS on proteostasis pathway in muscle of young and old mice. E) Experimental schema for intramuscular MS‐275 injection in the gastrocnemius muscle. The graphical elements in this study's figures are sourced from BioRender.com. F) Immunoblotting of H3K18la, H3K14la, H3K9la, and Lamin B1 in the gastrocnemius muscle with or without MS‐275 injection. H3 and GAPDH served as the loading controls. G) Relative band intensity of the immunoblots in F, *n* = 3. H) Distribution of the altered H3K9la peaks in genomic elements in muscle with or without MS‐275 treatment. I) The number of peaks at whole genome and peaks at promoter in muscle with or without MS‐275 treatment. J) Heatmaps and intensity profiles of H3K9la around ± 3 kb of TSS throughout the genome in muscle with or without MS‐275 treatment. K) Gene Ontology pathway analysis of genes with decreased H3K9la peaks at their promoters and corresponding downregulation with MS‐275 treatment. The error bars represent the S.D. of independent experiments. Two‐tailed, unpaired Student's *t* tests were performed. ****P < 0.001. TSS, transcription start site.

We previously showed that loss of histone lactylation accelerates cellular senescence by repressing genes involved in the cell cycle and DNA repair pathways. Considering that muscle cells are highly differentiated, we observed parallel downregulation of genes involved in DNA repair and a decline in the proteostasis pathway during muscle aging (**Figure** **6**A,B). These observations are consistent with previous reports that aging is associated with impaired DNA repair and loss of proteostasis.^[^ ^3^ ^]^

We further found that the histone lactylation levels of the promoters for DNA repair and proteostasis pathways were decreased during muscle aging (Figure 6C,D). In addition, key genes in DNA repair, such as *Pds5b*, *Park7*, *Eya1*, *Fam168a*, *Epc1*, *Rev3l*, *Ino80*, and *Exd2* ^[^ ^50^, ^51^, ^52^, ^53^, ^54^, ^55^, ^56^, ^57^ ^]^ and proteostasis, such as *Nedd4*, *Ecpas*, *Wwp1*, *Ubc*, *Fbxo3*, *Btrc*, *Senp1*, and *Fbxl17* ^[^ ^58^, ^59^, ^60^, ^61^, ^62^, ^63^, ^64^, ^65^ ^]^ were significantly downregulated due to loss of histone lactylation at their promoters (Figure S25, Supporting Information). Therefore, consistent with the findings we observed in cellular senescence, histone lactylation decreases during muscle aging and leads to the repression of genes associated with DNA repair and proteostasis. Besides, 20‐month‐old aged mice were intramuscularly injected with NALA for a week. We observed a significant upregulation in histone lactylation levels within the muscle tissue, concomitant with elevated Lamin B1 levels (Figure S26A, Supporting Information). Transcriptome sequencing further revealed that NALA injection could enhance the expression of pathways associated with muscle tissue function (including muscle adaptation, muscle cell development, muscle cell differentiation), proteostasis, and DNA repair. Additionally, NALA injection mitigates the heightened inflammatory response characteristic of muscle aging, such as cytokine‐mediated signaling pathways (Figure S26B, Supporting Information).

To further test the effects of histone lactylation on muscle aging, we continuously injected class I HDAC inhibitor MS‐275 into the gastrocnemius muscle of mice for 7 days and then assessed the histone lactylation levels and aging markers in the muscle (Figure 6E). Consistent with our previous results in cell experiments, inhibition of HDAC1 by MS‐275 resulted in a significant reduction in histone lactylation and a loss of Lamin B1 in muscle tissue (Figure 6F,G). Additionally, RNA‐seq analysis revealed that MS‐275 injection markedly accelerated muscle aging, primarily through the regulation of genes involved in genomic instability (*Cdkn1a*, *Ercc1*, *Ercc5*, and *Polb*), stem cell exhaustion (*Bmi1* and *Ccna2*), loss of proteostasis (*Tpp2*), cellular senescence (*Pdgfb*), SASP (*Mmp3*, *Mmp8*, *Mmp9*, *Mmp13*, *Irf5*, *Ccl5*, *Icam1*, and *Ptges*) and NF‐κB (*Tradd* and *Plcg1*) (Figure S27, Supporting Information). Besides, intramuscular MS‐275 administration decreased histone lactylation levels, with the decreased peaks predominantly located in promoter regions (Figure 6H–J). We also found that genes involved in DNA repair and proteostasis pathways lost histone lactylation at their promoters and were significantly downregulated after MS‐275 injection (Figure 6K and Figure S28, Supporting Information). These results demonstrate that the loss of histone lactylation in muscle downregulates DNA repair and proteostasis pathways, thereby impairing muscle function and accelerating muscle aging.

### 2.7 Exercise Activates Glycolysis and Restores Histone Lactylation Levels in Skeletal Muscle

**Figure 7:**
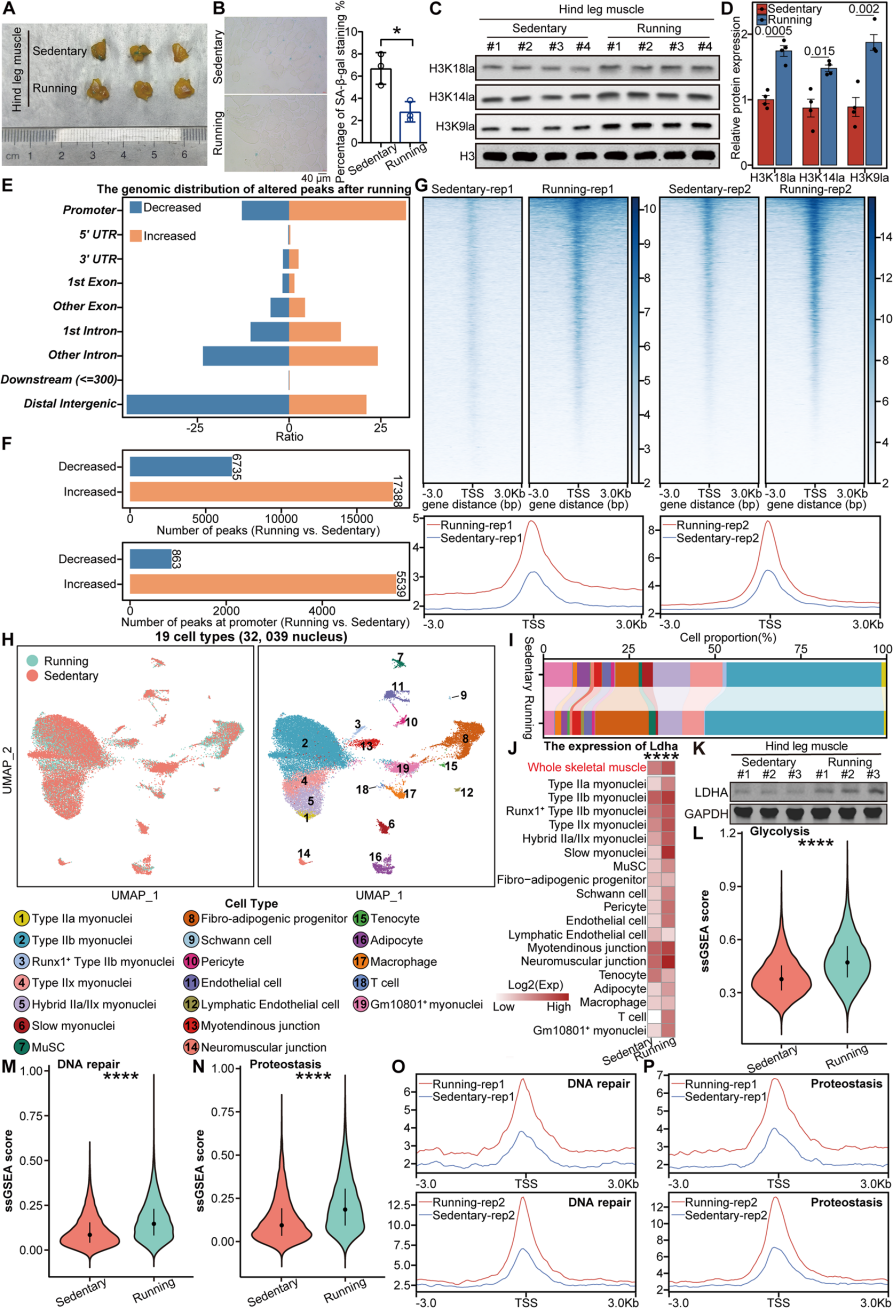
Exercise rescues muscle histone lactylation levels and restores DNA repair as well as proteostasis. A) SA‐β‐gal staining of skeletal muscle from the gastrocnemius legs of sedentary and running female mice. Three mice were measured in each group. B) SA‐β‐gal staining of sedentary and running hindleg skeletal muscle sections from 13‐month‐old female mice. Three mice were measured in each group. The percentage of SA‐β‐gal^+^ staining is shown on the right. C) Protein levels of H3K18la, H3K14la, and H3K9la in sedentary and running mice. GAPDH and H3 served as loading controls. D) Relative band intensity of immunoblots in C, *n* = 4. E) Distribution of the altered H3K9la peaks in genomic elements in muscle of sedentary and running mice. F) The number of peaks at whole genome and peaks at promoter in muscle of sedentary and running mice. G) Heatmaps and intensity profiles of H3K9la around ± 3 kb of TSS throughout the genome in muscle of sedentary and running mice. H) UMAP visualization of the 19 major cell types in muscle of sedentary and running mice. The left part shows the UMAP plot of the sample origin (*n*  =  2 per group). I) Stacked bar chart of the cell proportions in muscle of sedentary and running mice. J) The mRNA expression level of *Ldha* in the whole skeletal muscle and each cell type of sedentary and running mice. K) Protein levels of LDHA in the skeletal muscle of sedentary and running mice. GAPDH served as the loading control. L) Alterations in the expression of glycolysis hallmark genes in muscle between sedentary and running mice. M) The expression of genes related to DNA repair in muscle of sedentary and running mice. N) The expression of genes related to proteostasis in muscle of sedentary and running mice. O) Intensity profiles of H3K9la around ± 3 kb of TSS on DNA repair pathway in muscle of sedentary and running mice. P) Intensity profiles of H3K9la around ± 3 kb of TSS on proteostasis pathway in muscle of sedentary and running mice. The error bars represent the S.D. of independent experiments. Two‐tailed, unpaired Student's *t* tests were performed. *P < 0.05, ****P < 0.001.

To investigate possible intervention approaches for reversing the functional deterioration of aged skeletal muscle linked to histone lactylation loss, we trained middle‐aged mice with long‐term running exercise. Compared with that in sedentary mice, the percentages of SA‐β‐gal^+^ cells in skeletal muscle were lower in trained animals (**Figure** **7**A,B). After exercise, lactate levels and histone lactylation at H3K9, H3K14, and H3K18 were found to be upregulated (Figure 7C,D and Figure S29, Supporting Information). We then performed an H3K9la CUT&Tag experiment in gastrocnemius muscle from sedentary and running mice. Consistent with previous findings, running exercise resulted in a notable increase in histone lactylation levels compared to sedentary controls, with the elevated signals primarily localized to the promoter regions (Figure 7E–G). Subsequently, snRNA‐seq was performed to evaluate the potential roles of histone lactylation in improving the function of gastrocnemius muscle during animal exercise.^[^ ^66^ ^]^ After quality control, 32,039 nucleus were acquired for downstream analysis, and 19 cell types were identified in sedentary and running mice: Type IIa myonuclei, Type IIb myonuclei, Runx1^+^ Type IIb myonuclei, Type IIx myonuclei, Hybrid IIa/IIx myonuclei, Slow myonuclei, MuSC, Fibro‐adipogenic progenitor, Schwann cell, Pericyte, Endothelial cell, Lymphatic endothelial cell, Myotendinous junction, Neuromuscular junction, Tenocyte, Adipocyte, Macrophage, T cell, and Gm10801^+^ myonuclei (Figure 7H and Figure S30A, Supporting Information). A significant rearrangement of cell composition was observed in the skeletal muscle of running mice compared with that of sedentary mice. Following running exercise, we observed a significant increase in the proportions of Type IIb myonuclei and MuSC, while a marked decrease was found in the proportion of Adipocyte (Figure 7I), demonstrating that exercised mice acquired young muscle functions such as strength, endurance, and myofiber regeneration. Moreover, we identified that Adipocyte exhibited the greatest number of DEGs and the most sensitive to running, highlighting its profound transcriptomic changes after running exercise (Figure S30B, Supporting Information).

Furthermore, running exercise significantly increased the glycolytic capacity of skeletal muscle, as revealed by increased expression of key glycolysis genes such as Ldha (Figure 7J–L). And these observations were also true for most cell types, implying that these cell types may also undergo alterations in histone lactylation after running exercise (Figure 7J).

### 2.8 Exercise Increases Lactylation Levels and Promotes DNA Repair and Proteostasis

As a decrease in histone lactylation was observed during muscle aging and subsequently increased following running exercise, we investigated whether the decrease in DNA repair and proteostasis pathways during aging could be reversed by increased histone lactylation in running mice. Intriguingly, both pathways were significantly activated after running exercise (Figure 7M,N). Ultimately, the expression of DNA repair and proteostasis pathways is not only upregulated following running exercise, but there is also a significant enhancement of histone lactylation at the promoter regions of the key regulatory genes (Figure 7O,P and Figure S31, Supporting Information). These findings reveal that running exercise can restore histone lactylation, which is instrumental in enhancing DNA repair and proteostasis pathways, thereby positively influencing the function of skeletal muscle.

## 3 Discussion

**Figure 8:**
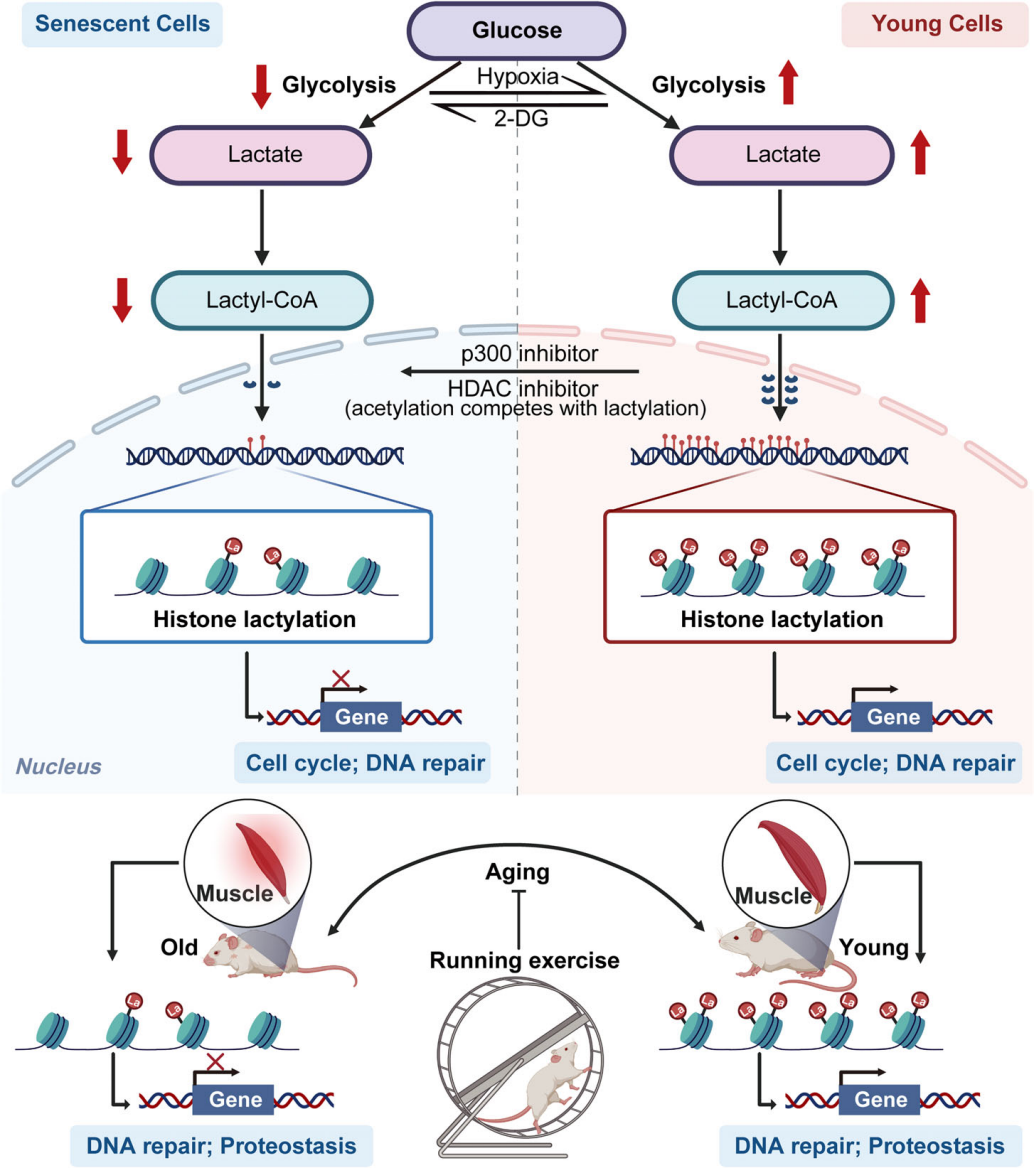
Schematic illustration. During cellular senescence and muscle aging, a significant decrease in histone lactylation is associated with the inhibition of glycolysis and the subsequent scarcity of lactate and lactyl‐CoA. Hypoxic reprogramming in primary cells can restore the level of histone lactylation, suggesting a positive correlation with a rejuvenated cellular state. Genome‐wide profiling revealed that histone lactylation predominantly occurs at the promoters of genes pivotal to the cell cycle and DNA repair pathways. Furthermore, a reduction in glycolysis leads to decreased histone lactylation during muscle aging. By enhancing glycolysis, exercise elevates histone lactylation levels, underscoring the critical role of exercise in the regulation of aging and the maintenance of muscle function, particularly through the modulation of DNA repair and proteostasis pathways. The graphical elements in this study's figures are sourced from BioRender.com.

Our research highlights the characteristics and importance of histone lactylation in the senescence and aging processes of skeletal muscle. Herein, we report a significant reduction in histone lactylation due to the inhibition of glycolysis, which occurs during both cellular senescence and muscle aging. Hypoxic reprogramming in primary cells can restore histone lactylation levels, indicating a positive link between this histone modification and a more youthful cellular state. The genome‐wide profiles revealed that histone lactylation primarily occurs at the promoters of genes that are key to the cell cycle and DNA repair pathways. Moreover, a reduction in glycolysis leads to a decrease in histone lactylation during muscle aging. Running exercise increases glycolysis and histone lactylation levels, underscoring the key role of this modification in aging regulation and muscle function maintenance, particularly through the modulation of DNA repair and proteostasis pathways (**Figure** **8**).

We found that histone lactylation participates in senescence progression by regulating cell cycle‐ and DNA repair‐related genes. Similarly, a recent report revealed that lactate‐driven lactylation of NBS1 promotes homologous recombination (HR)‐mediated DNA repair to facilitate chemotherapy resistance in cancer cells.^[^ ^67^ ^]^ Several other functions of histone lactylation, including the promotion of homeostasis in M1 macrophages^[^ ^17^ ^]^ and the activation of proliferation in ocular melanoma cells, have also been reported.^[^ ^22^ ^]^ The regulatory effect of histone lactylation on chromatin structure is also worthy of exploration. Moreover, exploring its potential roles across various organs, given its broad presence, may also yield valuable insights.

Our findings revealed a consistent decrease in histone lactylation levels in various cell lines, including MEFs, HUVECs, and IMR90, during senescence and in mouse tissues such as heart, liver, kidney, and skeletal muscle during aging. These findings suggest a conserved role for histone lactylation across different cell types and species in the aging process. However, variations in lactylation levels may also occur depending on the cell type, organ, and specific stage of senescence/aging, as well as at different histone sites not examined in this study. Notably, divergent lactylation patterns have been observed between brain and muscle tissues during aging, indicating the complexity of this epigenetic modification (data not disclosed).

The interplay between histone lactylation and other epigenetic modifications has been described in various studies,^[^ ^16^, ^21^, ^68^ ^]^ implying that the interaction of histone lactylation with other key epigenetic marks, such as histone acetylation and DNA methylation, also deserves further exploration within the contexts of senescence and aging. Our results and those reported by others highlight the multifaceted roles of histone lactylation in a plethora of physiological and pathological processes.^[^ ^17^ ^]^ Furthermore, akin to recent studies, our data reveal the intriguing competitive dynamics between histone lactylation and acetylation at specific genomic regions in certain conditions. The sensitivity of histone lactylation to lactate levels, both exogenous and endogenous, is highly pronounced. The levels of lactylation and acetylation of histones may mirror the availability of acetyl‐CoA and lactyl‐CoA, respectively, and their balance reflects metabolic flux through pyruvate. However, during replicative senescence and muscle aging, histone acetylation changes are not consistently similar to or reversed relative to histone lactylation. Instead, it is the decrease in histone lactylation that primarily modulates aging‐related pathways, thus histone lactylation plays a more significant role in regulating aging processes. Further investigation is needed to understand how histone lactylation interacts with other histone modifications to influence senescence and aging.

## 4 Conclusion

In summary, our study significantly advances the understanding of the impact of histone lactylation on cellular senescence and tissue aging processes. We also reveal new histone lactylation‐related mechanisms that underlie the beneficial effects of physical exercise on slowing cellular senescence, alleviating aging‐related phenotypes, and enhancing skeletal muscle function.

## 5 Experimental Section

### Cell Culture

IMR90 cells were purchased from the American Type Culture Collection (ATCC), HUVECs were purchased from AllCells, and MEFs were isolated from 12.5‐ to 14‐day‐old embryos of C57BL/6 mice. IMR90, MEF, NIH‐3T3 and 293T cells were cultured in DMEM (Fisher Scientific) supplemented with 10% fetal bovine serum (FBS, Fisher Scientific), and nonessential amino acids (NEAA, 100×, Macgene) were added for IMR90 cell culture. For the physiological hypoxia experiments, IMR90 and MEF cells were cultured in a hypoxic chamber (STEMCELL, 27310) containing mixed gas (3% O_2_, 5% CO_2_, 92% N_2_). All other cells were cultured at 37 °C with 5% CO_2_. For 2‐DG treatment, IMR90 cells were cultured with 2‐DG for 5 days, roughly in accordance with the number of passages carried out under hypoxic conditions.

### Mouse Models

C57BL/6 mice were purchased from Charles River. All animal experiments met the requirements of the Institutional Animal Care and Use Committee of Peking University (AAALAC unit number: 001249, approval number: SYXK20190032). In the aging model, 3‐month‐old mice as the young group and 20‐month‐old mice as the old group were selected. To assess behavioral changes associated with muscle aging, rotarod performance tests and treadmill fatigue tests on 20‐month‐old aged mice and 3‐month‐old young mice were conducted. In the MS‐275 injection model, 11‐month‐old female mice received intramuscular injections in the gastrocnemius muscle, with a week‐long regimen of MS‐275 at a concentration of 20 mg kg^−1^. In the NALA injection model, 500mg k^−1^ NALA injections to 20‐month‐old mice for one week were administered. In the running exercise model, 11‐month‐old middle‐aged mice were used and subjected them to a 2‐month period of exercise. For long‐term running exercise on a treadmill, the speed was set to 9 m min^−1^, and the running time was 30 min for each training session. The running exercise lasted for 2 months, with 2–3 days of rest after each training session. Five exercised mice ran for 2 min at 6 m min^−1^ to adapt to the treadmill each time. Besides, the muscle tissues used in this study were all from the mouse gastrocnemius muscle.

### Behavioral Performance Test

For the rotarod performance test, the initial speed was set to 5 rpm, and the final speed was 32 rpm, with an acceleration time of 300 s. Five mice in each group were tested on three consecutive days, and the latency to fall (s) was recorded. For the treadmill fatigue test, five mice in each group were tested on three separate days, with an interval of one day. The slope of the treadmill was set to 5°, and the speed (m/min) was increased as follows: 10 (2 min), 14 (5 min), 16 (5 min), 18 (5 min), and 20 (5 min). The running time to fatigue was recorded.

### Senescence‐Associated β‐Galactosidase Staining (SA‐β‐Gal Staining)

Cells were seeded onto 6‐well plates and cultured to the required density, after which SA‐β‐gal staining was performed via a Senescence Cells Histochemical Staining Kit (Sigma, CS0030). The cells were incubated with the staining mixture overnight at 37 °C. Images were acquired via a DMI 6000B microscope at 10 × 10 magnification (Leica). ImageJ software (NIH) was used to analyze the percentage of SA‐β‐gal^+^ signals. The experimental procedures for SA‐β‐gal staining of skeletal muscle were performed in 15‐mL tubes, and images were acquired with a camera.

Tissues for SA‐β‐gal staining were sectioned under frozen conditions, followed by gentle rinsing in PBS after thawing. Each rinse lasted for 5 min, and this process was repeated three times. The tissue sections were circled with a histochemical pen, and an appropriate volume of staining fixative solution was added to cover the tissue adequately, followed by a 30‐min fixation. The fixative solution was removed, and the tissue was washed three times with PBS, each lasting 8 min. After the PBS was removed, each tissue sample was incubated in 30 µl of SA‐β‐gal staining solution, ideally ensuring complete submersion, and sealed with plastic wrap to prevent evaporation. The samples were incubated at 37 °C overnight. On the following day, the working solution was discarded, and the tissue sections were sealed with a coverslip for observation and imaging under an optical microscope. ImageJ software (NIH) was used to analyze the percentage of SA‐β‐gal^+^ signals.

### Immunofluorescence

Cells were seeded onto sterile coverslips in 10‐cm plates and cultured to the required density. The cells were fixed for 10 min with 4% paraformaldehyde, blocked for 30 min with 1% bovine serum albumin (BSA) at room temperature, and incubated overnight at 4 °C with an anti‐Ki67 antibody (Abcam, ab15580, 1:500). The next day, the cells were incubated with Alexa Fluor 594 Donkey anti‐Rabbit IgG (Life Technologies) for 2 h at room temperature and then stained with 1 ng/µL DAPI for 3 min. Immunofluorescence images were acquired from sealed coverslips using a fluorescent microscope (Leica).

Upon the return of tissue sections to room temperature, antigen retrieval was initiated. Permeabilization was carried out at room temperature with 0.5% Triton X‐100 for 20 min, followed by a 1‐hour incubation in BSA for blocking. The primary antibody was appropriately diluted in antibody dilution buffer and left to incubate at 4 °C overnight. On the subsequent day, the humid chamber was removed and allowed to equilibrate for 30 min, followed by a thorough washing with 1xPBS. The fluorescent secondary antibody was diluted in antibody dilution buffer and incubated at room temperature for 2 hours, with meticulous light avoidance. Subsequently, mounting was accomplished using glycerol supplemented with an antifading agent, and DAPI staining was applied to the cell nuclei as necessary. Immunofluorescence images were acquired from sealed coverslips using a fluorescent microscope (Leica).

### Immunoblotting

TRIzol (Invitrogen, 15596018) was used to extract proteins from cells or tissues, which were separated via SDS‒PAGE and transferred to nitrocellulose membranes. The membranes were incubated with 5% skim milk for 1 h, followed by incubation with the following specific antibodies overnight at 4 °C: anti‐Lamin B1 (Proteintech, 12987‐1‐AP, 1:1000), anti‐p16 (Proteintech, 10883‐1‐AP, 1:500), anti‐p21 (Santa Cruz Biotechnology, sc397, 1:500), anti‐β‐ACTIN (Santa Cruz Biotechnology, sc‐47778, 1:1000), anti‐α‐TUBULIN (Sigma, T6199, 1:5000), anti‐GAPDH (Proteintech, 60004‐1‐Ig, 1:10000), anti‐l‐lactyllysine (pan‐Kla, PTM BIOLABS, PTM1401, 1:1000), anti‐H3K9la (PTM BIOLABS, PTM1419RM, 1:1000), anti‐H3K14la (PTM BIOLABS, PTM1414, 1:1000), and anti‐LDHA (Proteintech, 21799‐1‐AP, 1:5000). The next day, the membranes were incubated with IRDye 800CW goat/donkey anti‐mouse/rabbit antibodies (LI‐COR Biosciences, 926‐32210, 1:10000) for 2 h at room temperature. Images were acquired via an Odyssey Infrared Imaging System (LI‐COR).

### Lactate Measurement

l‐Lactate levels were measured via a lactate assay kit (Abcam, ab65331), which facilitated the quantification of lactate levels in cells and tissues.

### CUT&Tag Sequencing (CUT&Tag‐seq)

Cells were harvested via trypsin digestion at room temperature and then counted. CUT&Tag experiments were performed according to the CUT&Tag Assay Kit manual (Vazyme, TD903). Briefly, cell pellets were collected at 600 × g for 5 min, washed with wash buffer, and incubated with ConA beads for 10 min at room temperature in 8‐strip tubes. The cell–ConA bead complexes were subsequently incubated with anti‐H3K9la (PTM BIOLABS, PTM1419RM) at 4 °C overnight and with a secondary antibody for 1 h at room temperature. The pA/G‐Tnp transposon mediated the transposition reaction, and TTBL mediated DNA fragmentation. DNA extraction beads were used to extract CUT&Tag‐enriched DNA, which was then subjected to library amplification for Illumina sequencing. The PCR products were purified via VAHTS DNA Clean Beads (Vazyme, N411) and sequenced on the NovaSeq 6000 PE150 platform at GENEWIZ.

### RNA‐seq

For bulk RNA‐seq, total RNA was extracted from cells using TRIzol (Invitrogen, 15596018). The cDNA library construction and Illumina sequencing were performed by Novogene (HiSeq PE150) or GENEWIZ (NovaSeq 6000 PE150). For snRNA‐seq, fresh mouse skeletal muscle was frozen in liquid nitrogen and used for library construction and sequencing by BGI Genomics and 10X Genomics.

### qPCR

All‐in‐One Supermix (TransGen Biotech, AT341) was used to generate cDNA from total RNA. cDNA or ChIP DNA was measured by qPCR using a LightCycler 96 Instrument (Roche) after the addition of SYBR Green premix (Vazyme, Q712). The primers used for RT‒qPCR and ChIP‒qPCR are listed in Tables S1 and S2 (Supporting Information), respectively.

### Hematoxylin and Eosin (H&E) Staining

Skeletal muscle was paraffin embedded, sectioned, and stained sequentially with hematoxylin and eosin. Images were acquired using a NIKON Eclipse Ci microscope (Nikon).

### Bulk RNA‐seq Data Processing

The raw sequencing data were processed using trim_galore (version 0.6.10) to remove adapters and low‐quality reads with the following parameters: ‐q 25 –phred33 –stringency 4 –length 36 ‐e 0.1. The trimmed reads were then mapped to the hg38 or mm10 reference genome using hisat2 (version 2.2.1), and the sam files were converted to sorted bam files using samtools (version 1.7). The gene expression count matrix was obtained using featureCounts (version 2.0.6) and normalized with DESeq2 (version 1.34.0). The thresholds for DEGs were identified by |log_2_(FC)| > 1 or 0.58, and adjusted *P* value < 0.05.

### CUT&Tag Data Processing

The adapters and low‐quality sequences were removed for downstream analysis using trim_galore (version 0.6.10). Then, the clean data were mapped to the hg38 or mm10 reference genome using Bowtie2 (version 2.2.5) with the following parameters: –end‐to‐end –very‐sensitive –no‐mixed –no‐discordant –phred33 ‐I 10 ‐X 700. The sorted bam files were converted to bigWig format using bamCoverage in deepTools (version 3.5.1) with the following parameters: –normalizeUsing RPKM. In addition, computeMatrix, plotHeatmap, and plotProfile in deepTools were utilized for calculating and visualizing signal intensities ±3 kb of TSS. Peak calling was performed using MACS2 (version 2.2.7.1) with the parameters ‐f BAMPE ‐g hs ‐q 0.1. For samples with biological replicates, the R package DESeq2 (version 1.34.0) was used to identify altered peaks as defined by |log_2_(FC)| > 1 and *P* value < 0.05, edgeR (version 3.36.0) and MACS2 were used to identify altered peaks for samples with single biological replicate. Peaks distribution and annotation were analyzed using the R package ChIPseeker (version 1.30.3).

### Processing of Raw Data, Filtering, Integration, Clustering, and Identification of Cell Types

For DNBelab C of MGI, the raw fastq‐format data of snRNA‐seq was aligned to the mm10 reference genome and the filtered gene expression count matrix was obtained using PISA (version 0.12).^[^ ^69^ ^]^ For 10x Genomics, the filtered gene expression count matrix was generated using Cell Ranger (version 6.1.2) aligned to mm10 reference genome. The R package Seurat (version 4.1.1) was used to perform the downstream analysis. For each sample, genes detected in at least 3 cells expressing at least 200 genes were kept. Subsequently, cells with the following three criteria were retained: more than 200 detected genes, fewer than 6000 detected genes, and fewer than 6% mitochondrial genes. The “RunHarmony” function in the R package harmony (version 1.0) was used to remove batch variances. Meanwhile, mitochondrial genes and ribosomal genes were removed to avoid noise in downstream analysis.

The “RunPCA” function was used to perform dimensionality reduction, and then the nearest‐neighbor graph was constructed using the “FindNeighbors” function. Cell clusters were identified using the “FindClusters” and “RunUMAP” functions. Cell type‐specific marker genes were calculated with the cutoff of |log_2_FC| > 0.25 and adjusted *P* value < 0.05 using the “FindAllMarkers” function. Cell types were annotated using canonical marker genes,^[^ ^48^, ^66^, ^70^, ^71^, ^72^ ^]^ including *Myh2* and *Ppara* for Type IIa myonuclei; *Myh4*, *Pde4d*, and *Mylk4* for Type IIb myonuclei; *Runx1* and *Sh3d19* for Runx1^+^ Type IIb myonuclei; *Myh1* for Type IIx myonuclei; *Myh7* and *Tpm3* for Slow myonuclei; *Pax7* and *Chodl* for MuSC; *Dcn* and *Abca8a* for Fibro‐adipogenic progenitor; *Cdh19* and *Mpz* for Schwann cell; *Rgs5* and *Cacna1c* for Pericyte; *Ptprb* and *Pecam1* for Endothelial cell; *Mmrn1* and *Prox1* for Lymphatic endothelial cell; *Col22a1* and *Ankrd1* for Myotendinous junction; *Ano4* and *Col4a3* for Neuromuscular junction; *Thbs4* and *Fmod* for Tenocyte; *Pde3b* and *Acsl1* for Adipocyte; *Tbxas1* and *Rbpj* for Macrophage; *Skap1* and *Il7r* for T cell; *Cd79a* and *Ms4a1* for B cell; *Gm10801* for Gm10801^+^ myonuclei.

### Aging‐Related and Running‐Related Differential Expression Analysis

For each cell type, DEGs between old and young mice, as well as between running and sedentary mice, were calculated using the “FindMarkers” function with the Wilcoxon rank sum test. Genes with |log_2_FC| > 0.25 and adjusted *P* value < 0.05 were identified as aging‐related or running‐related DEGs specific to each cell type. For the global DEGs between old and young mice, as well as between running and sedentary mice, the threshold for DEGs was set as |log_2_FC| > 0.15 and adjusted *P* value < 0.05.

### Transcriptional Noise Analysis

To investigate transcriptional fluctuation during aging, we analyzed aging‐related transcriptional noise in each cell type using the approach described by Angelidis et al.^[^ ^73^ ^]^ Cells were down sampled to equalize UMI count, and old and young cells within each cell type were standardized to the same number to eliminate differences in total UMI counts as well as cell type frequencies. Transcriptional noise for each cell was quantified by computing the Euclidean distance from the respective cell type mean between old and young groups.

### Cell Type Prioritization Analysis

Augur (version 1.0.3)^[^ ^74^ ^]^ was used to prioritize aging‐sensitive cell types in the Old versus Young group and running‐sensitive cell types in the Running versus Sedentary group. The results were further visualized using the R package ggplot (version 3.4.2).

### Gene Set Score Analysis

Gene sets were downloaded from the MSigDB database (version 2023.1). Specifically, pathways “HALLMARK_GLYCOLYSIS”, “GOBP_REGULATION_OF_CELL_CYCLE_PROCESS”, “GOBP_DNA_REPAIR”, “GOBP_PROTEOLYSIS_INVOLVED_IN_PROTEIN_CATABOLIC_PROCESS”, “SenMayo”,^[^ ^75^ ^]^ “GOBP_POSITIVE_REGULATION_OF_INFLAMMATORY_RESPONSE”, and “REACTOME_MACROAUTOPHAGY” were respectively used to evaluate the glycolysis, cell cycle, DNA repair, proteostasis, SASP, inflammatory, and autophagy pathways.

For snRNA‐seq data, the gene sets were then used to calculate scores for each cell using method “ssGSEA” of R package GSVA (version 1.40.1). A Wilcoxon rank‐sum test was performed to compare the old and young mice, as well as the running and sedentary mice. For CUT&Tag data, deepTools was used to evaluate the intensity of H3K9la for cell cycle, DNA repair, and proteostasis pathways.

### Functional Enrichment Analyses

Metascape(version 3.5)^[^ ^76^ ^]^ and clusterProlifer (version 4.6.2)^[^ ^77^ ^]^ was used to perform Gene Ontology (GO) Biological Processes enrichment analyses. The representative terms were selected with the parameter *P* value < 0.05. The results were further visualized with R package ggplot2.

### Statistical Analysis

The statistical analysis is conducted via a two‐tailed Student's *t* test and one‐way ANOVA in GraphPad Prism 9.5.1 and R software. The results are presented as the mean ± S.D., with statistical significance defined as *P* < 0.05. Significance levels are denoted as *P < 0.05, **P < 0.01, ***P < 0.001, and ****P < 0.0001.

## Conflict of Interest

The authors declare no conflict of interest.

## Supporting information



Supporting Information

## Data Availability

All sequencing data from this study are available through the Gene Expression Omnibus under accession number GSE226008.
