# Supplementary material for: Histone Lactylation Antagonizes Senescence and Skeletal Muscle Aging by Modulating Aging‐Related Pathways
Source: Adv Sci (Weinh). 2025 May 19;12(22):2412747. doi: 10.1002/advs.202412747 (PMC12165025; doi:10.1002/advs.202412747)
Supplement: Supplementary file 1 — Supporting Information [file ADVS-12-2412747-s001.docx]

**Histone Lactylation Antagonizes Senescence and Skeletal Muscle Aging by Modulating Aging-Related Pathways**

Fanju Meng^1, #^ **|** Jianuo He^1, #^ **|** Xuebin Zhang^1, #^ **|** Wencong Lyu^1, #^ **|** Ran Wei^1^ **|** Shiyi Wang^1^ **|** Zhehao Du^1^ **|** Haochen Wang^1^ **|** Jinlong Bi^1^ **|** Xueyang Hua^2^ **|** Chao Zhang^4^ **|** Yiting Guan^5^ **|** Guoliang Lyu^1^ **|** Xiao-Li Tian^3^ **|** Lijun Zhang^1, *^ **|** Wenbing Xie^2, *^ **|** Wei Tao^1, 6, *^

^1^The State Key Laboratory of Membrane Biology, School of Life Sciences, Peking University, Beijing 100871, China

^2^Hefei National Laboratory for Physical Sciences at the Microscale, School of Basic Medical Sciences, Division of Life Sciences and Medicine, University of Science and Technology of China, Hefei 230026, China

^3^Department of Human Population Genetics, Human Aging Research Institute (HARI) and School of Life Sciences, Nanchang University, Nanchang 330031, China

^4^Kunming Institute of Zoology, Chinese Academy of Sciences, Kunming, Yunnan, 650201, China

^5^Zhanjiang Institute of Clinical Medicine, Zhanjiang Central Hospital, Guangdong Medical University, Zhanjiang, 524045, China

^6^Lead Contact

^#^These authors contributed equally to this work

^*^Correspondence

Wei Tao, Email: [weitao@pku.edu.cn](mailto:weitao@pku.edu.cn).

Wenbing Xie, Email: [wxie6@ustc.edu.cn](mailto:wxie6@ustc.edu.cn).

Lijun Zhang, Email: [lj_zhang@pku.edu.cn](mailto:lj_zhang@pku.edu.cn).

Funding information

The National Key Research and Development Project, Grant No. 2021YFA0909300, 2023YFC3603300. The National Natural Science Foundation of China, Grant No. 32241006, 82192891.

Supplementary figures and figure legends (S1-S31)

Supplementary tables (Table S1-2)

**
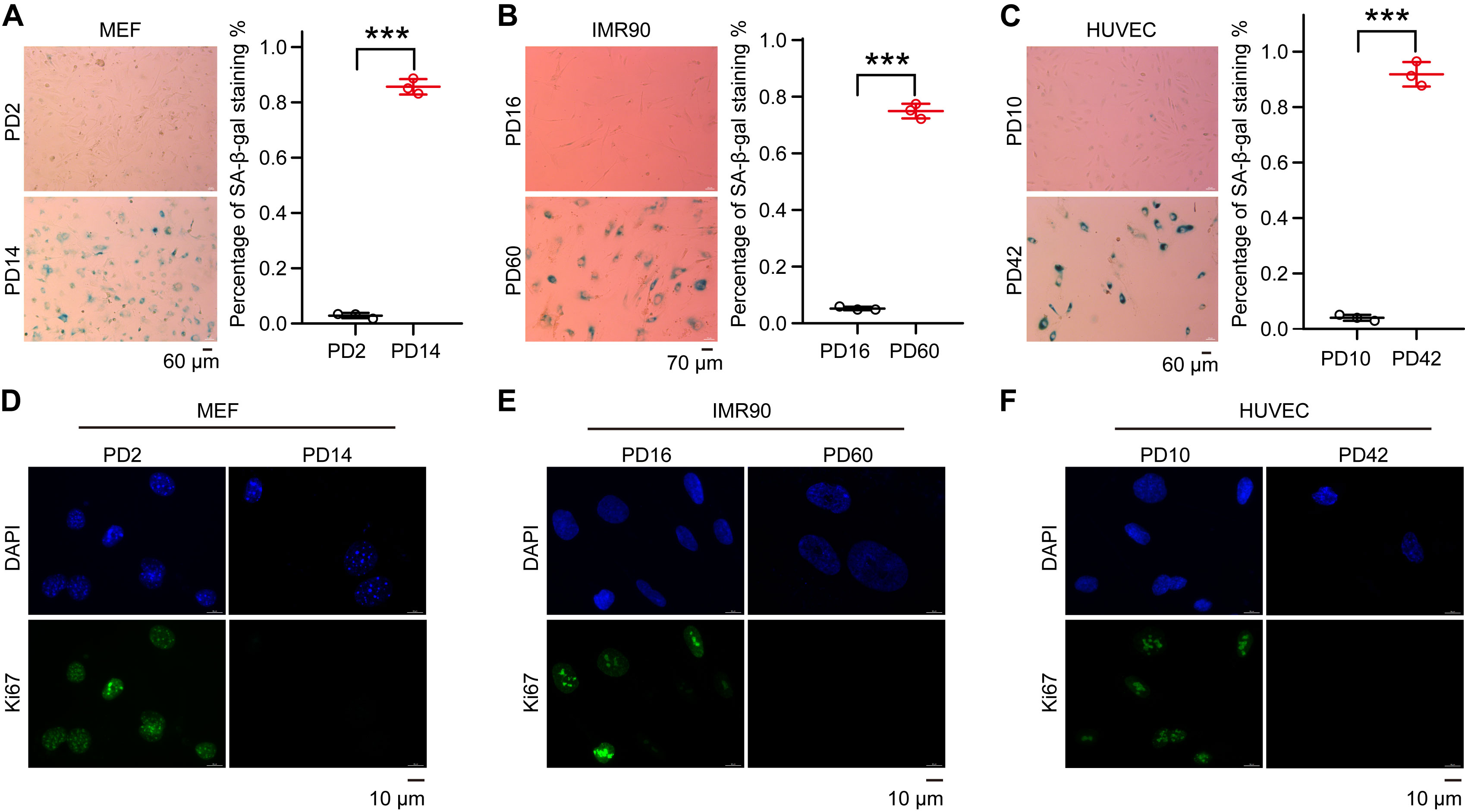
Fig. S1. Increased SA-β-gal^+^ staining and reduced proliferative capacity are associated with senescence. A-C**, SA-β-gal staining of young and senescent MEFs (**A)**, IMR90 cells (**B**), and HUVECs (**C**). The percentages of SA-β-gal^+^ cells are shown on the right of the respective figures. PD, population doubling (the total number of cells in each population doubled during *in vitro* culture). **D-F**, Immunofluorescence of Ki67 and DAPI in young and senescent MEFs (**D**), IMR90 cells (**E**), and HUVECs (**F**). The error bars represent the S.D. of independent experiments, n = 3. Two-tailed, unpaired Student’s *t* tests were performed. ****P* < 0.001.

**
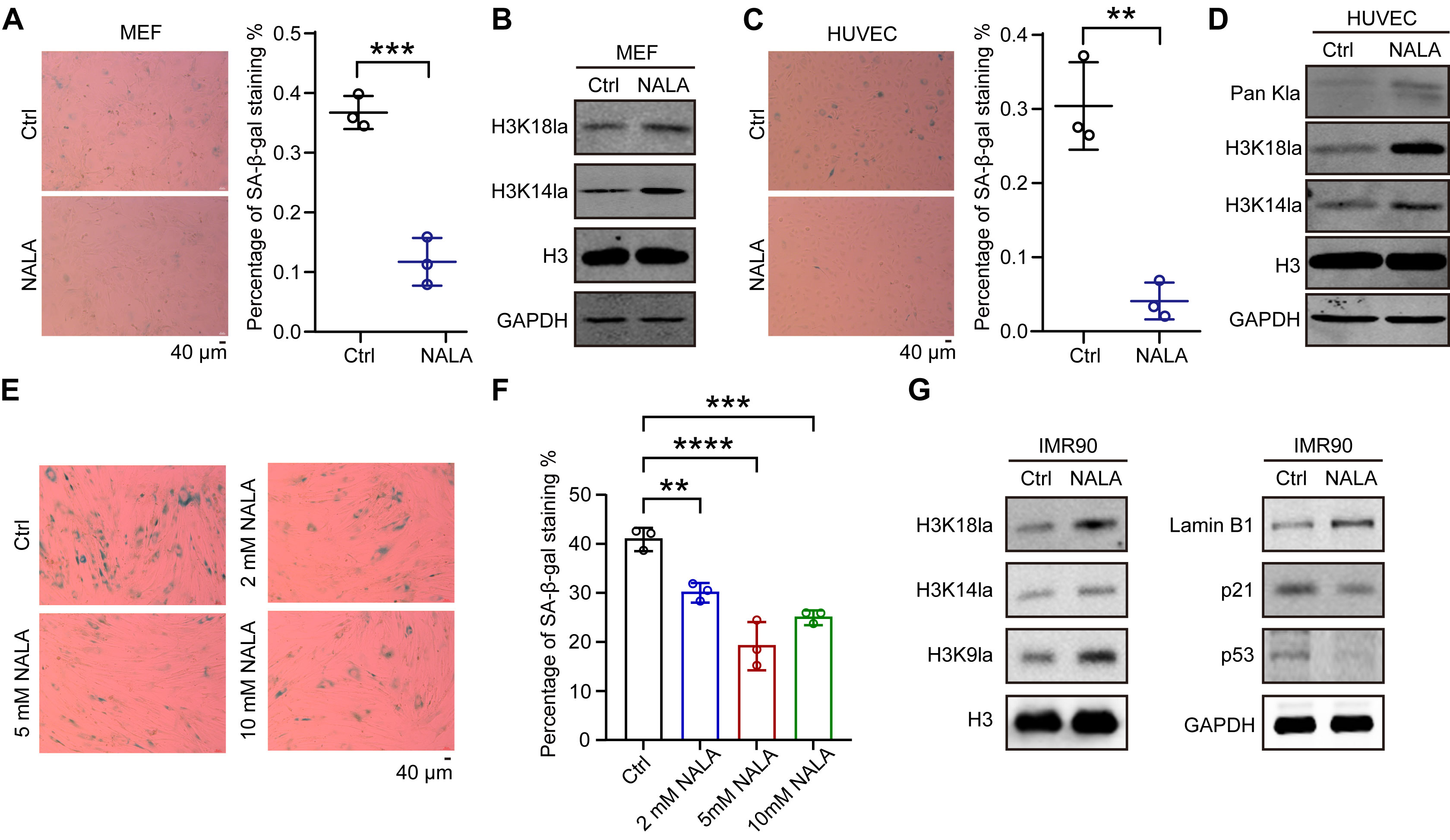
Fig. S2. NALA treatment ameliorates replicative senescence.** **A**, SA-β-gal staining of MEFs treated with NALA. The percentages of SA-β-gal^+^ cells are shown on the right. **B**, Immunoblotting of H3K18la and H3K14la levels in MEFs treated with NALA. H3 and GAPDH served as the loading controls. **C**, SA-β-gal staining of HUVECs treated with NALA. The percentages of SA-β-gal^+^ cells are shown on the right. **D**, Immunoblotting of Pan Kla, H3K18la, and H3K14la levels in MEFs treated with NALA. H3 and GAPDH served as the loading controls. **E**, SA-β-gal staining of IMR90 cells treated with NALA. **F**, The percentages of SA-β-gal^+^ cells are shown on the right. **G**, Immunoblotting of H3K9la, H3K14la, H3K18la, Lamin B1, p21, and p53 levels in IMR90 cells treated with NALA. H3 and GAPDH served as the loading controls. The error bars represent the S.D. of independent experiments, n = 3. Two-tailed, unpaired Student’s *t* tests were performed in **A** and **C**. One-way ANOVA was performed in **F**. ***P* < 0.01, ****P* < 0.001, *****P* < 0.0001.

**
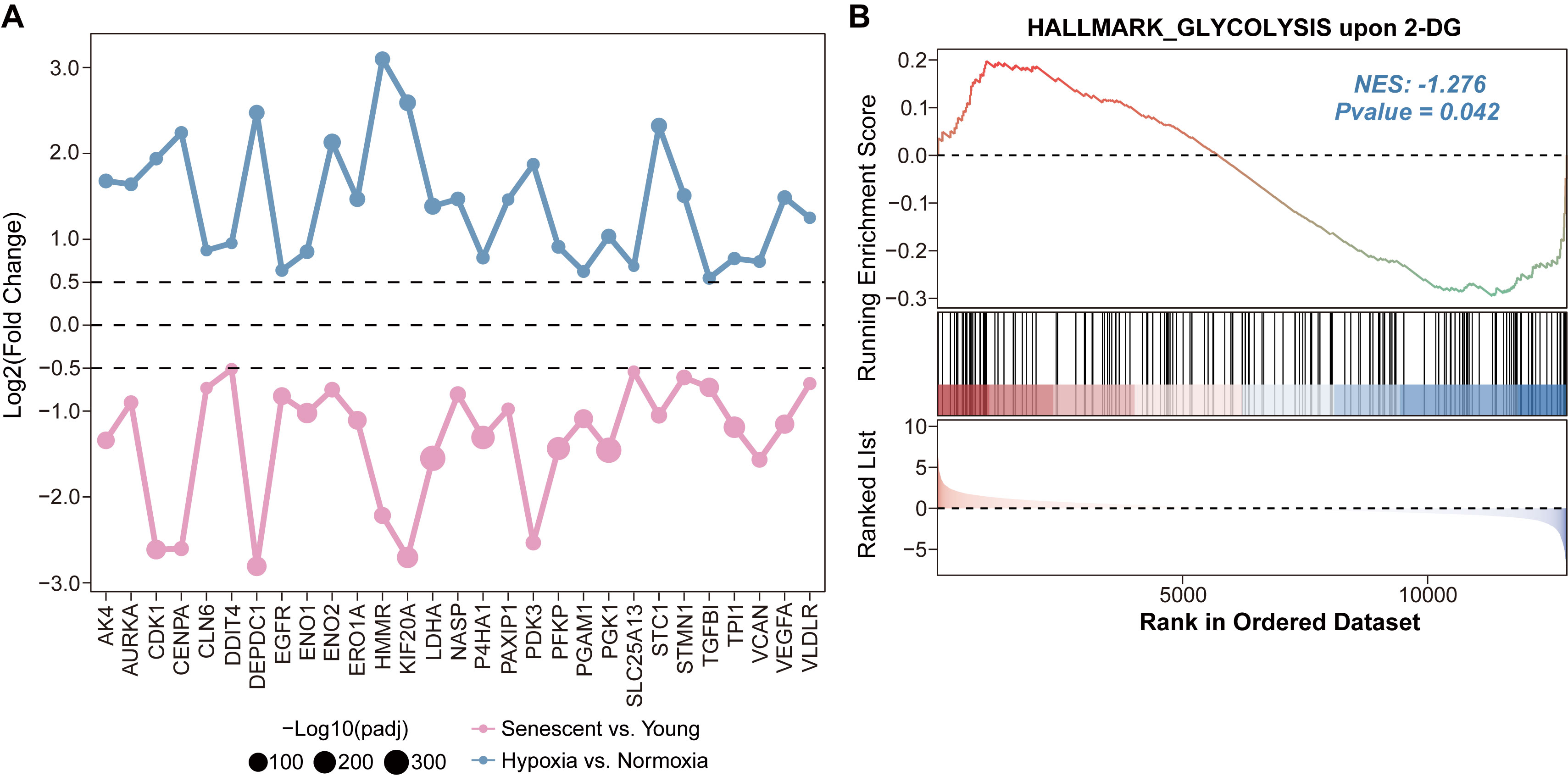
Fig. S3. Altered expression of glycolysis genes during senescence, hypoxia exposure, and 2-DG treatment. A**, Expression levels of glycolysis hallmark genes during senescence or hypoxia. **B**, GSEA of glycolysis genes in IMR90 cells upon 2-DG treatment.

**
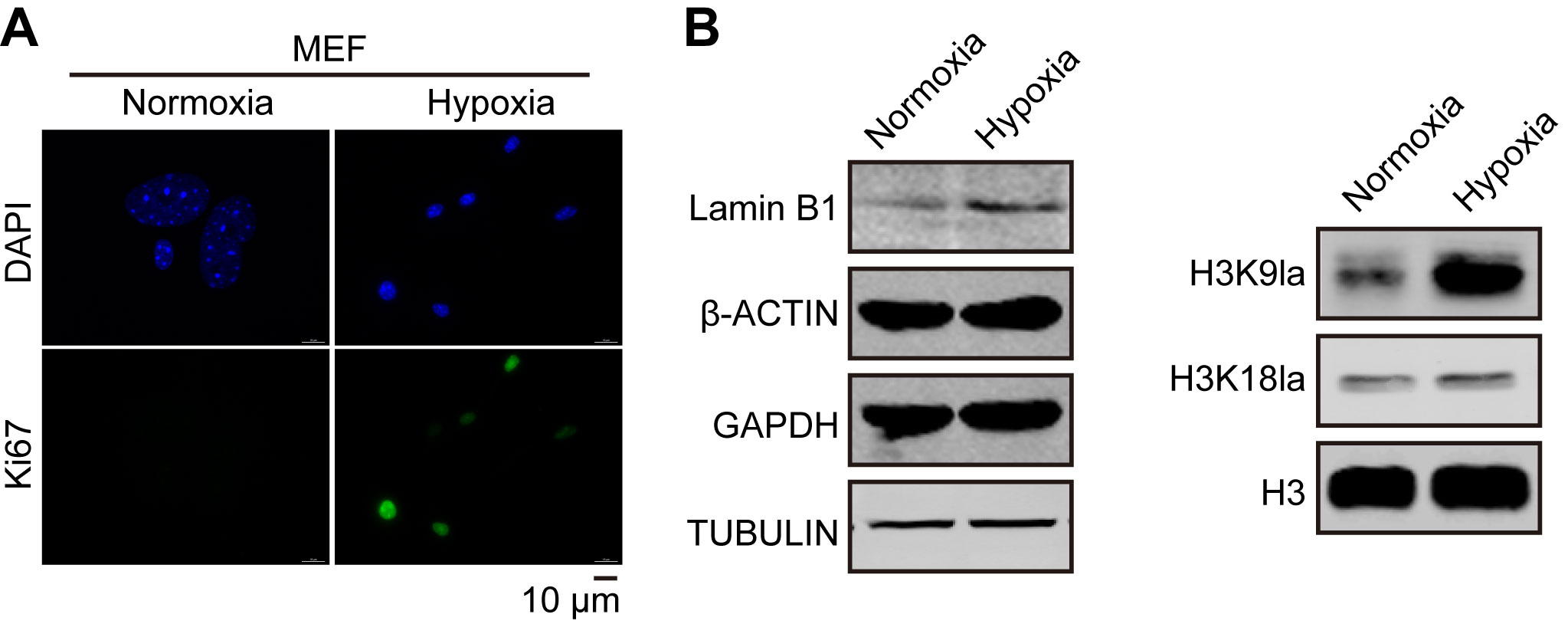
Fig. S4. Hypoxia mitigates senescence and restores histone lactylation in MEFs. A**, Immunofluorescence of Ki67 in MEFs cultured under normoxia and hypoxia. DAPI was used to stain the nuclei. **B**, Immunoblotting of Lamin B1, H3K9la, and H3K18la in MEFs cultured under normoxia and hypoxia. β-ACTIN, GAPDH, TUBULIN, and H3 served as loading controls.

**
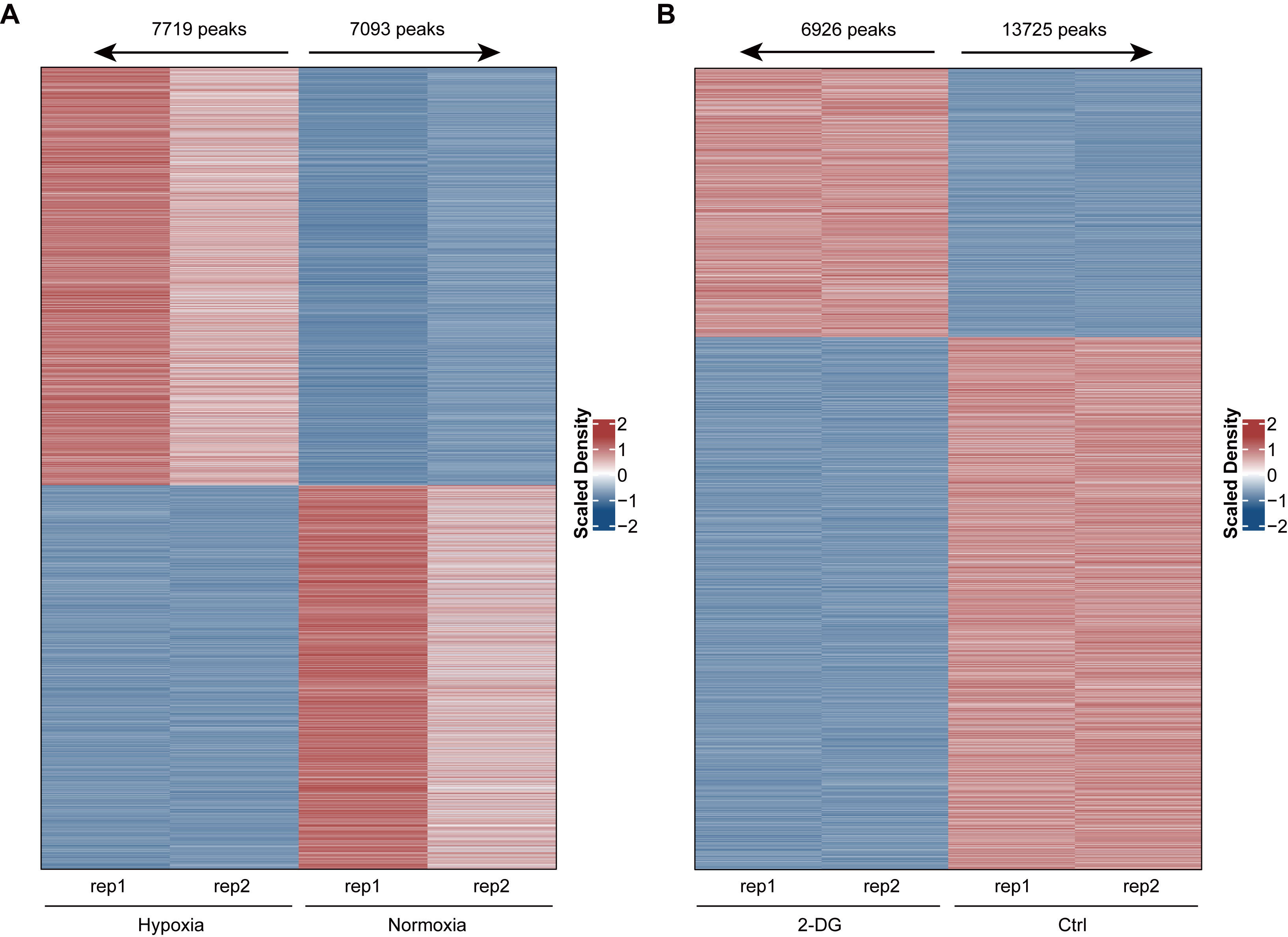
Fig. S5. Hypoxia exposure increases H3K9la while 2-DG treatment decreases H3K9la. A**, Heatmap of altered H3K9la peaks upon hypoxia. **B**, Heatmap of altered H3K9la peaks upon 2-DG treatment.

**
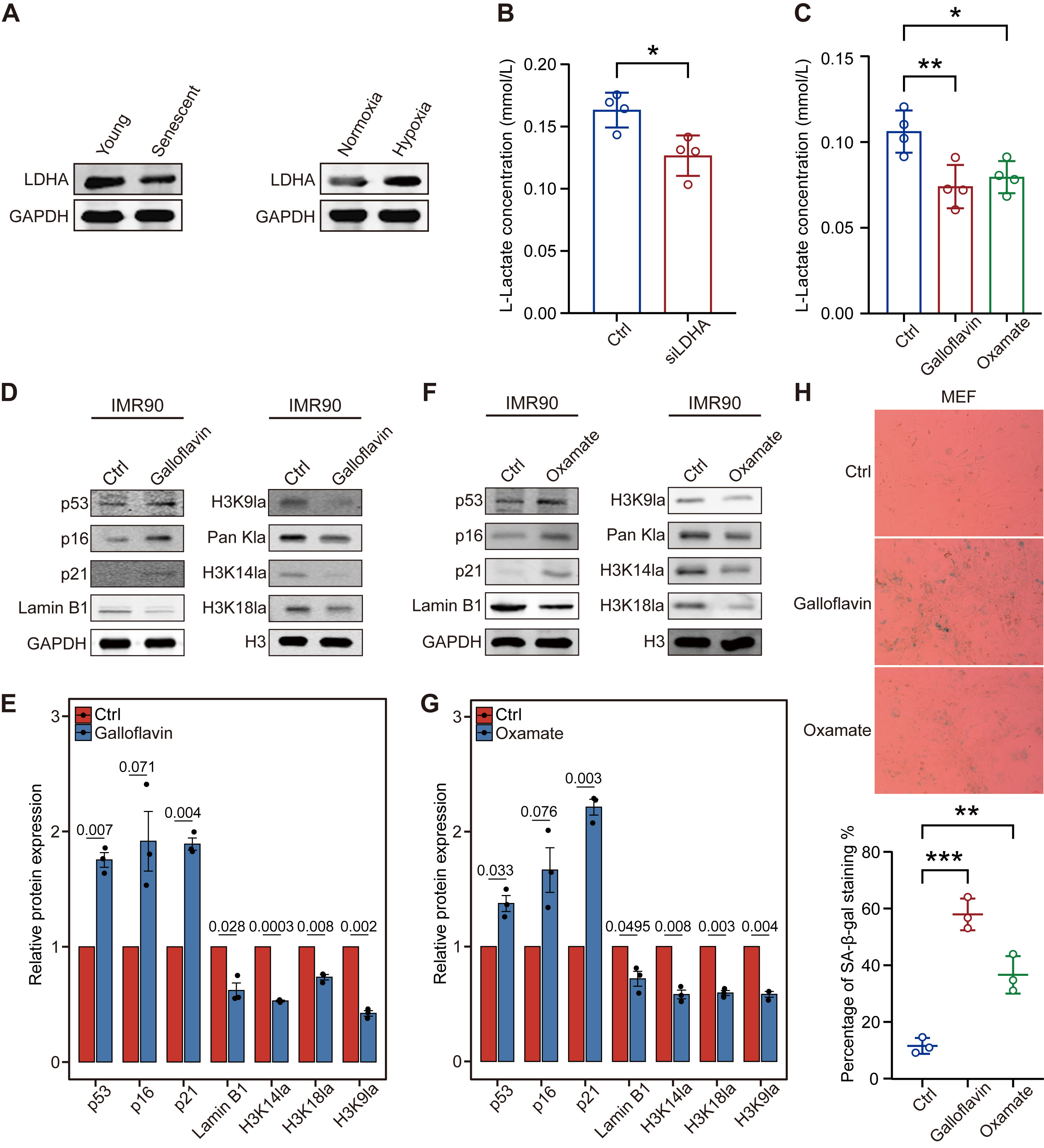
Fig. S6. The inhibition of LDHA results in decreased lactate levels and cellular senescence.** **A**, Protein levels of LDHA in IMR90 cells cultured during senescence and under normoxia and hypoxia. GAPDH served as the loading control. **B**, Lactate levels in IMR90 cells with or without siRNA treatment, n = 4. **C**, Lactate levels in IMR90 cells with or without galloflavin and oxamate treatment, n = 4. **D**, Protein levels of p53, p16, p21, Lamin B1, Pan Kla, H3K9la, H3K14la, and H3K18la in IMR90 cells cultured with or without galloflavin treatment. GAPDH and H3 served as loading controls. **E**, Relative band intensity of the immunoblots in **D**, n=3. **F**, Protein levels of p53, p16, p21, Lamin B1, Pan Kla, H3K9la, H3K14la, and H3K18la in IMR90 cells cultured with and without oxamate treatment. GAPDH and H3 served as loading controls. **G**, Relative band intensity of immunoblots in **F**, n=3. **H**, SA-β-gal staining of MEFs with or without galloflavin and oxamate, n = 3. The percentages of SA-β-gal^+^ cells are shown at the bottom. Two-tailed, unpaired Student’s *t* tests were performed in **B**. One-way ANOVA was performed in **C** and **H**. The error bars represent the S.D. of independent experiments. **P* < 0.05, ***P* < 0.01, ****P* < 0.001.

**
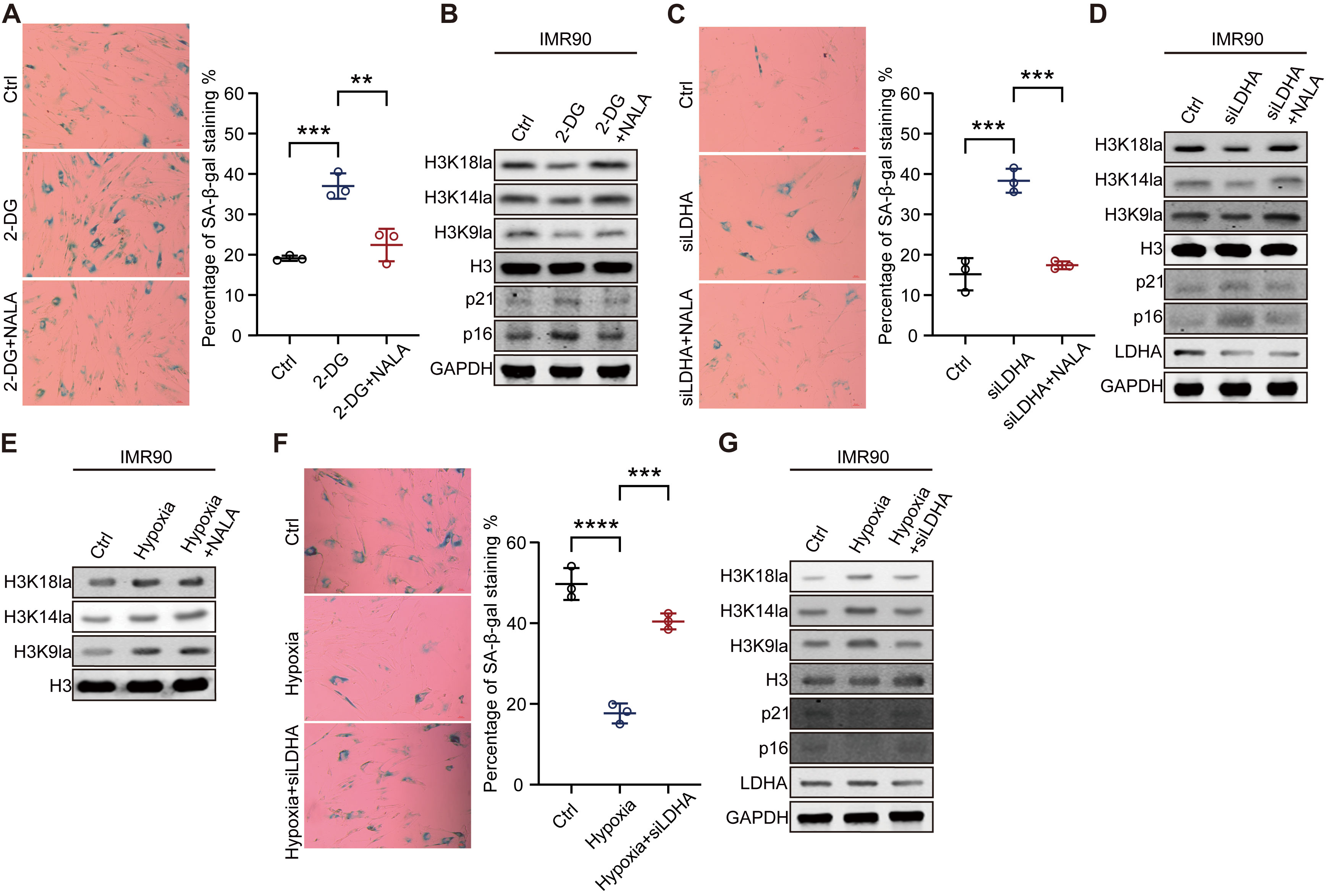
**

**Fig. S7.** **Lactate restores histone lactylation and blocks cell senescence.** **A**, SA-β-gal staining of IMR90 cells in the presence or absence of 2-DG and 2-DG + NALA. The percentages of SA-β-gal^+^ cells are shown on the right. **B**, Immunoblotting of H3K18la, H3K14la, H3K9la, p16, and p21 of IMR90 cells in the presence or absence of 2-DG and 2-DG + NALA. H3 and GAPDH served as the loading controls. **C**, SA-β-gal staining of IMR90 cells in the presence or absence of si*LDHA* and si*LDHA* + NALA. The percentages of SA-β-gal^+^ cells are shown on the right. **D**, Immunoblotting of H3K18la, H3K14la, H3K9la, p16, and p21 of IMR90 cells in the presence or absence of si*LDHA* and si*LDHA* + NALA. H3 and GAPDH served as the loading controls. **E**, Immunoblotting of H3K18la, H3K14la, and H3K9la of IMR90 cells in the presence or absence of hypoxia and hypoxia + NALA. H3 served as the loading control. **F**, SA-β-gal staining of IMR90 cells in the presence or absence of hypoxia and hypoxia + si*LDHA*. The percentages of SA-β-gal^+^ cells are shown on the right. **G**, Immunoblotting of H3K18la, H3K14la, H3K9la, p16, and p21 of IMR90 cells in the presence or absence of hypoxia and hypoxia + si*LDHA*. H3 and GAPDH served as the loading controls. The error bars represent the S.D. of independent experiments, n = 3. One-way ANOVA was performed. ***P* < 0.01, ****P* < 0.001, *****P* < 0.0001.

**
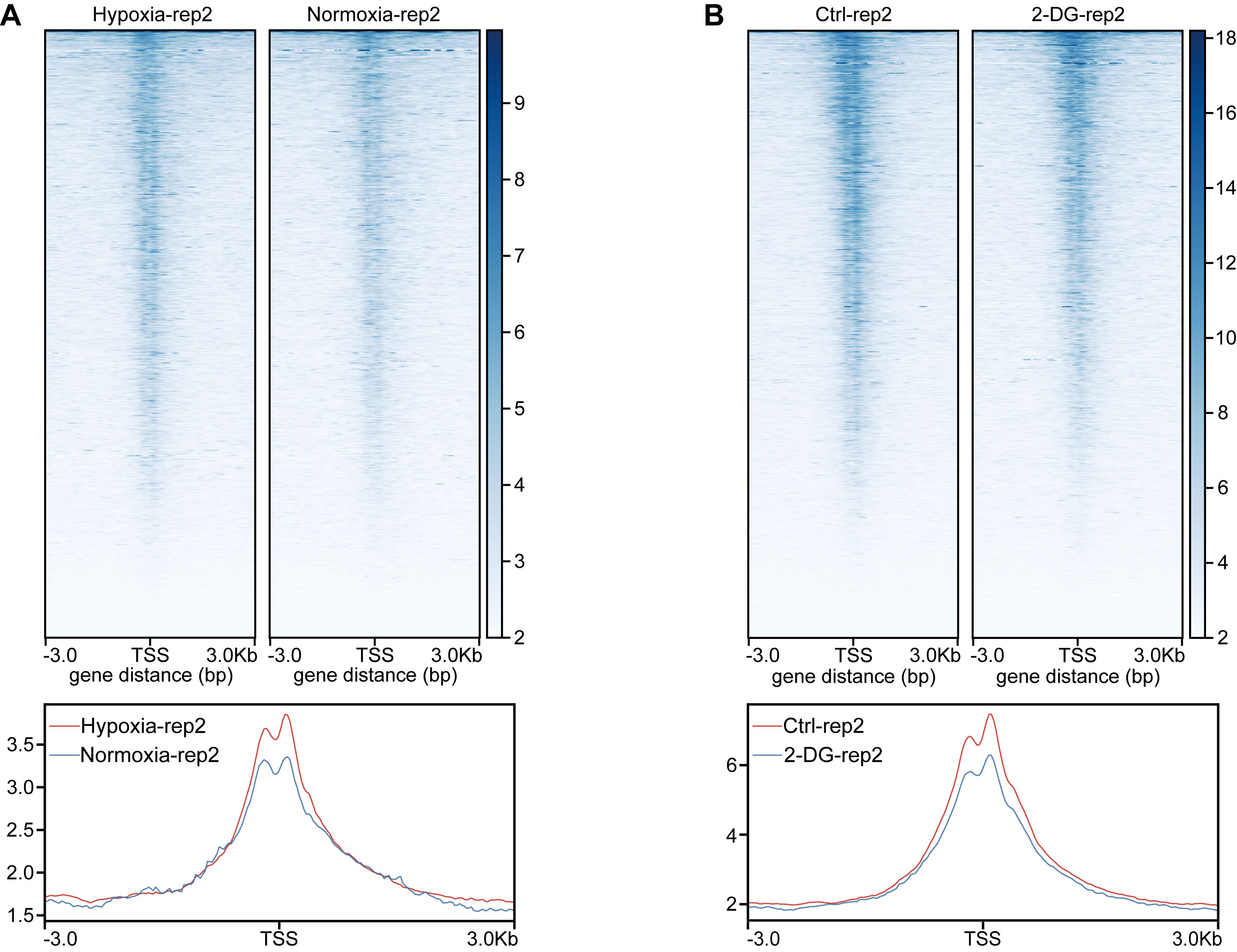
Fig. S8. Histone lactylation levels are increased under hypoxia exposure while decreased under 2-DG treatment at promoter.** **A**, Heatmaps and intensity profiles of H3K9la around ± 3 kb of TSS throughout the genome in IMR90 cells cultured under normoxia or hypoxia. **B**, Heatmaps and intensity profiles of H3K9la peak signals around ± 3 kb of TSS throughout the genome in IMR90 cells cultured with or without 2-DG treatment. TSS, transcription start site.

**
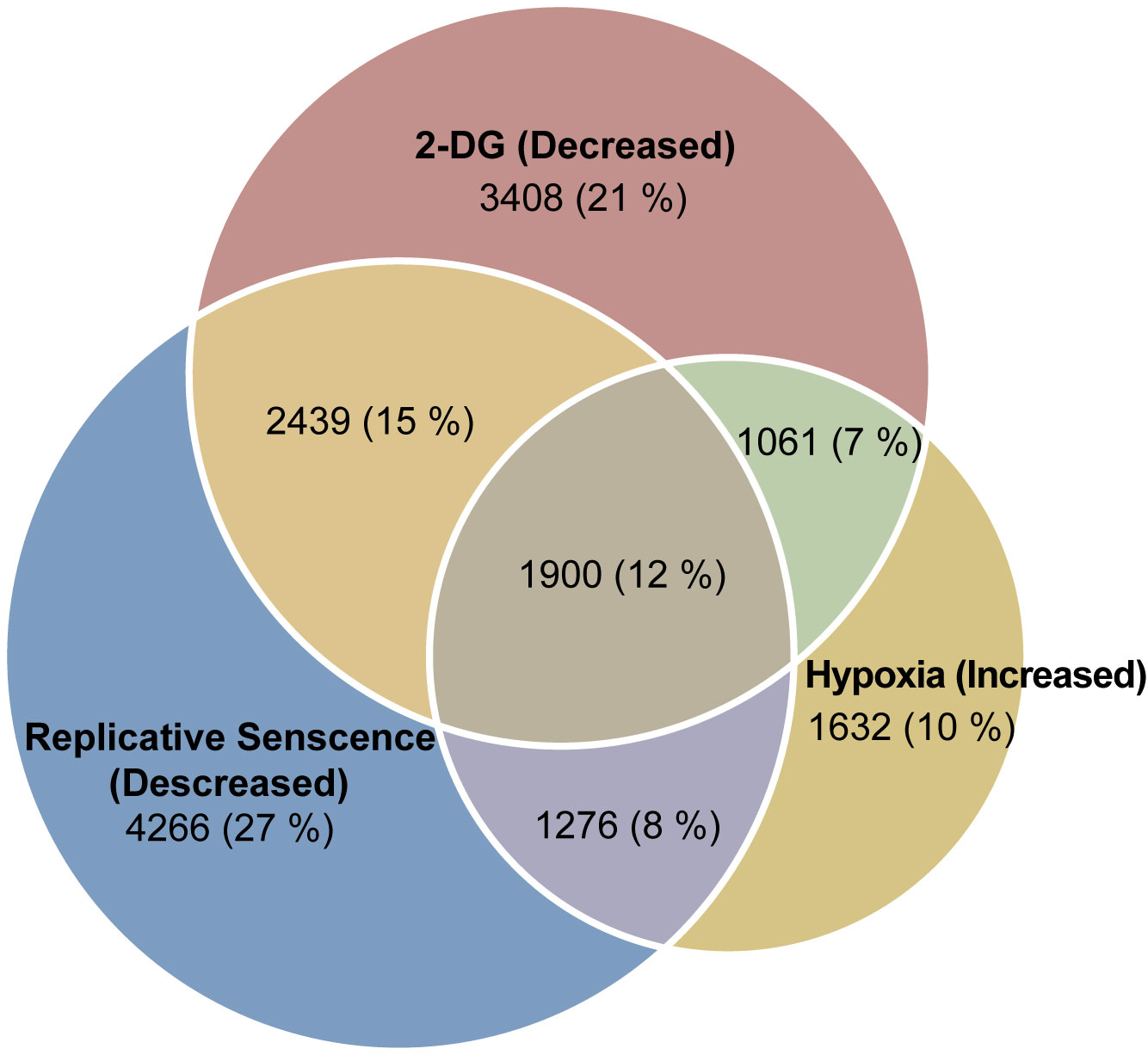
Fig. S9.** **The overlap of genes associated with decreased H3K9la peaks during cell senescence, increased H3K9la peaks under hypoxia, and decreased H3K9la peaks with 2-DG treatment is significant.** The Venn diagram showing the overlap of genes associated with decreased H3K9la peaks during cell senescence, increased H3K9la peaks under hypoxia, and decreased H3K9la peaks with 2-DG treatment.

**
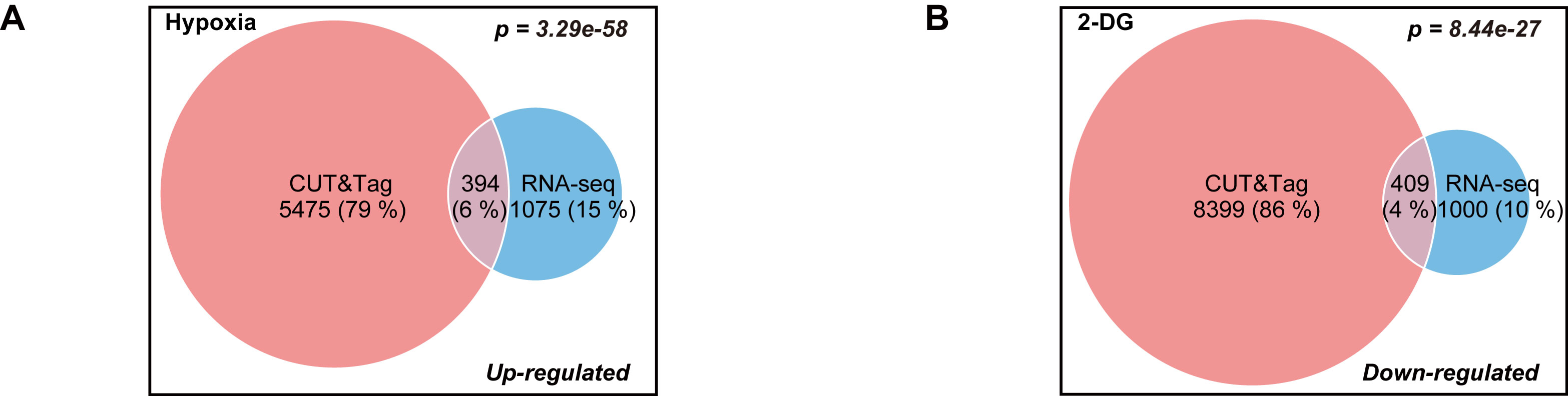
**

**Fig. S10. The overlap of DEGs and altered peaks associated genes under hypoxia or 2-DG treatment is significant. A**, Venn diagram showing the intersection between increased peak-associated genes of CUT&Tag and upregulated genes in the RNA-seq data obtained under hypoxia. **B**, Venn diagram showing the overlapping genes between decreased H3K9la peak-associated genes identified via CUT&Tag and downregulated genes identified via RNA-seq data upon 2-DG.

**
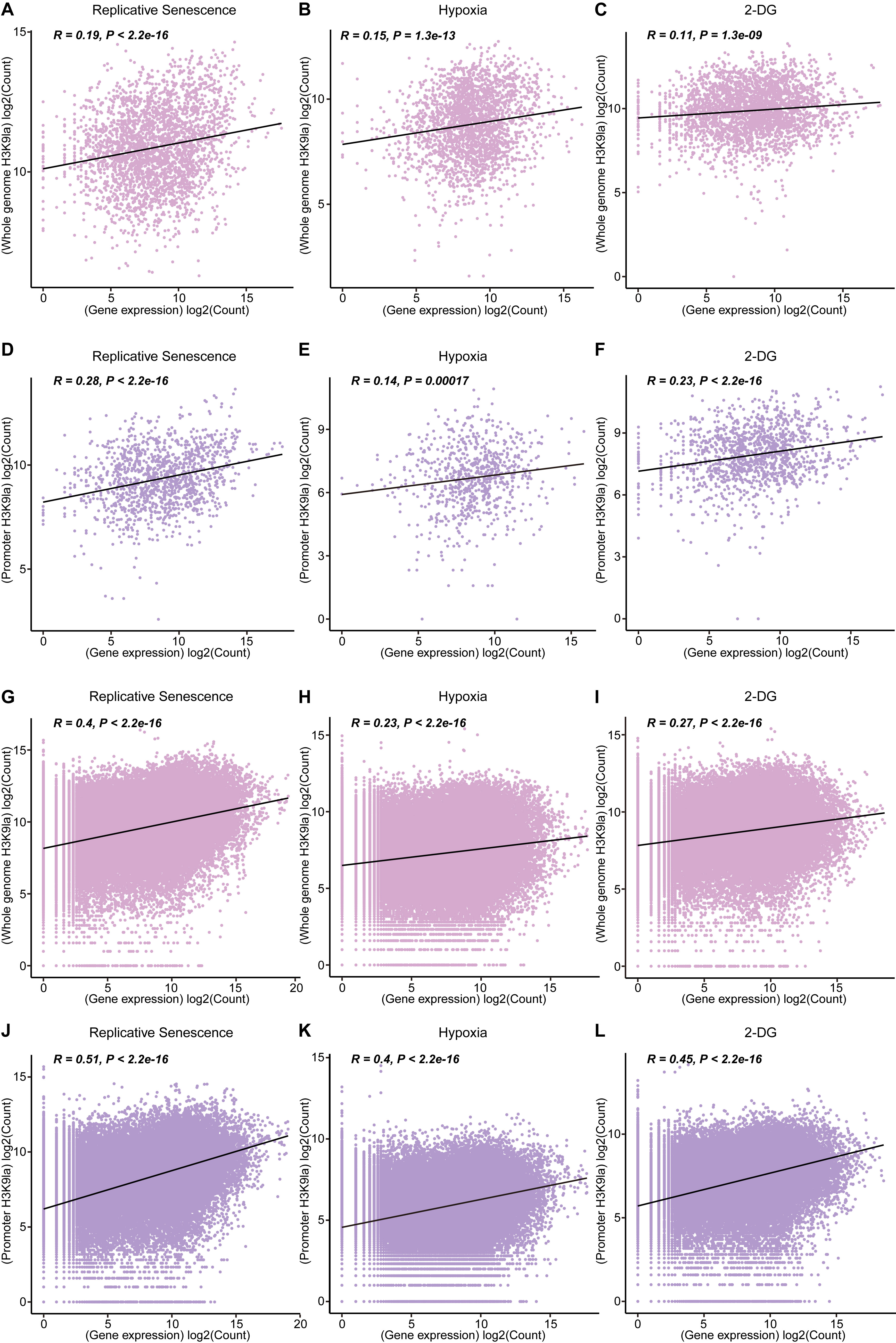
Fig. S11. Histone lactylation exhibits positive correlation with gene expression in replicative senescence, hypoxia, and 2-DG treatment. A-C**, Scatter plots showing the correlation between altered H3K9la peaks in the whole genome (log2Count, y-axis) and the corresponding differentially expressed genes expression (log2Count, x-axis) for replicative senescence (**A**), hypoxia (**B**), and 2-DG treatment (**C**). **D-F**, Scatter plots showing the correlation between altered H3K9la peaks at promoter (log2Count, y-axis) and the corresponding differentially expressed genes expression (log2Count, x-axis) for replicative senescence (**D**), hypoxia (**E**), and 2-DG treatment (**F**). Pearson’s correlation coefficient R and P value are indicated. **G-I**, Scatter plots showing the correlation between all H3K9la peaks at promoter (log2Count, y-axis) and the corresponding gene expression (log2Count, x-axis) for replicative senescence (**G**), hypoxia (**H**), and 2-DG treatment (**I**). **J-L**, Scatter plots showing the correlation between all H3K9la peaks in the whole genome (log2Count, y-axis) and the corresponding gene expression (log2Count, x-axis) for replicative senescence (**J**), hypoxia (**K**), and 2-DG treatment (**L**). Pearson’s correlation coefficient R and P value are indicated.


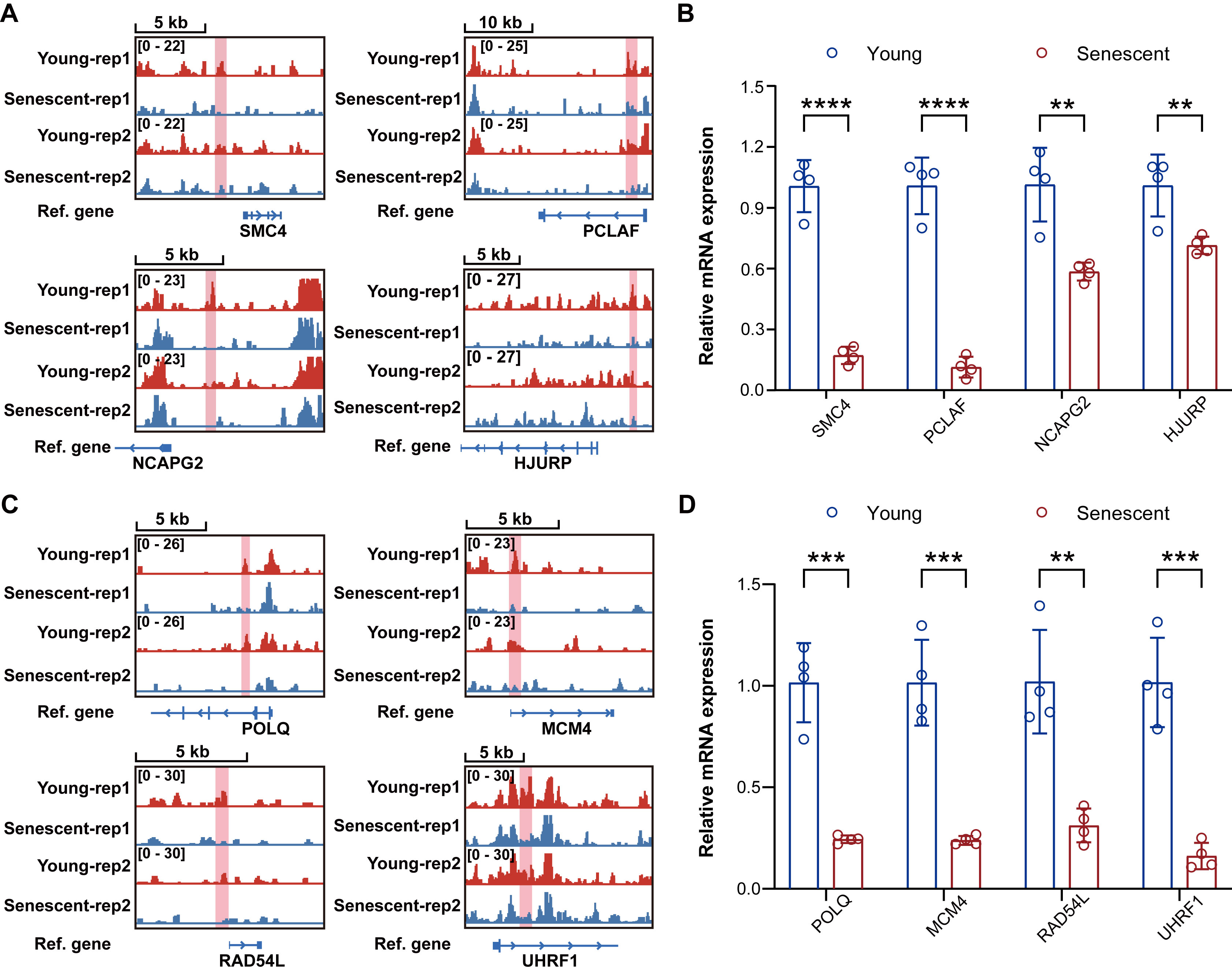


**Fig. S12. Histone lactylation regulates genes involved in the cell cycle and DNA repair pathways during cellular senescence. A**, Snapshots of H3K9la peaks at the promoters of *SMC4*, *PCLAF*, *NCAPG2*, and *HJURP* in young and senescent IMR90 cells. **B**, mRNA levels of *SMC4*, *PCLAF*, *NCAPG2*, and *HJURP* in young and senescent IMR90 cells.**C**, Snapshots of H3K9la peaks at the promoters of *POLQ*, *MCM4*, *RAD54L*, and *UHRF1* in young and senescent IMR90 cells.**D**, mRNA levels of *POLQ*, *MCM4*, *RAD54L*, and *UHRF1* in young and senescent IMR90 cells. The cycle threshold (Ct) values of these genes were normalized to that of *ACTB*. The error bars represent the S.D. of independent experiments, n = 4. Two-tailed, unpaired Student’s *t* tests were performed. ***P* < 0.01, ****P* < 0.001, *****P* < 0.0001.

**
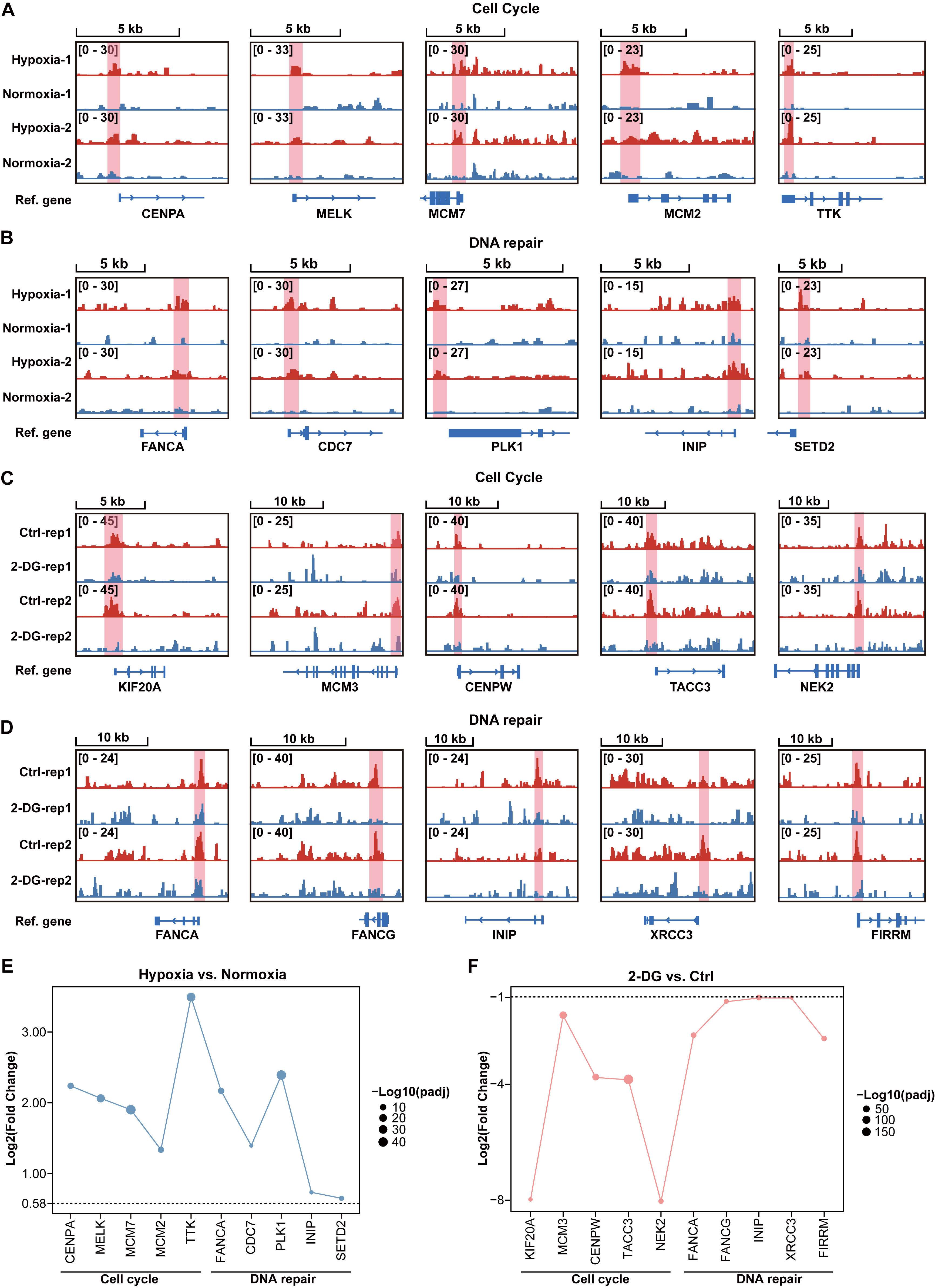
Fig. S13.** **Histone lactylation and gene expression of cell cycle and DNA repair genes increase under hypoxia but decrease with 2-DG treatment. A**, **B**, Snapshots of H3K9la peaks at the promoters of cell cycle- (**A**) and DNA repair- (**B**) related genes in IMR90 cells cultured under normoxia and hypoxia. **C**, **D**, Snapshots of H3K9la peaks at the promoters of cell cycle- (**C**) and DNA repair- (**D**) related genes in IMR90 cells cultured with or without 2-DG treatment. **E**, **F**, mRNA levels of the cell cycle- and DNA repair- related genes in IMR90 cells cultured under normoxia and hypoxia (**E**) and with or without 2-DG treatment (**F**).


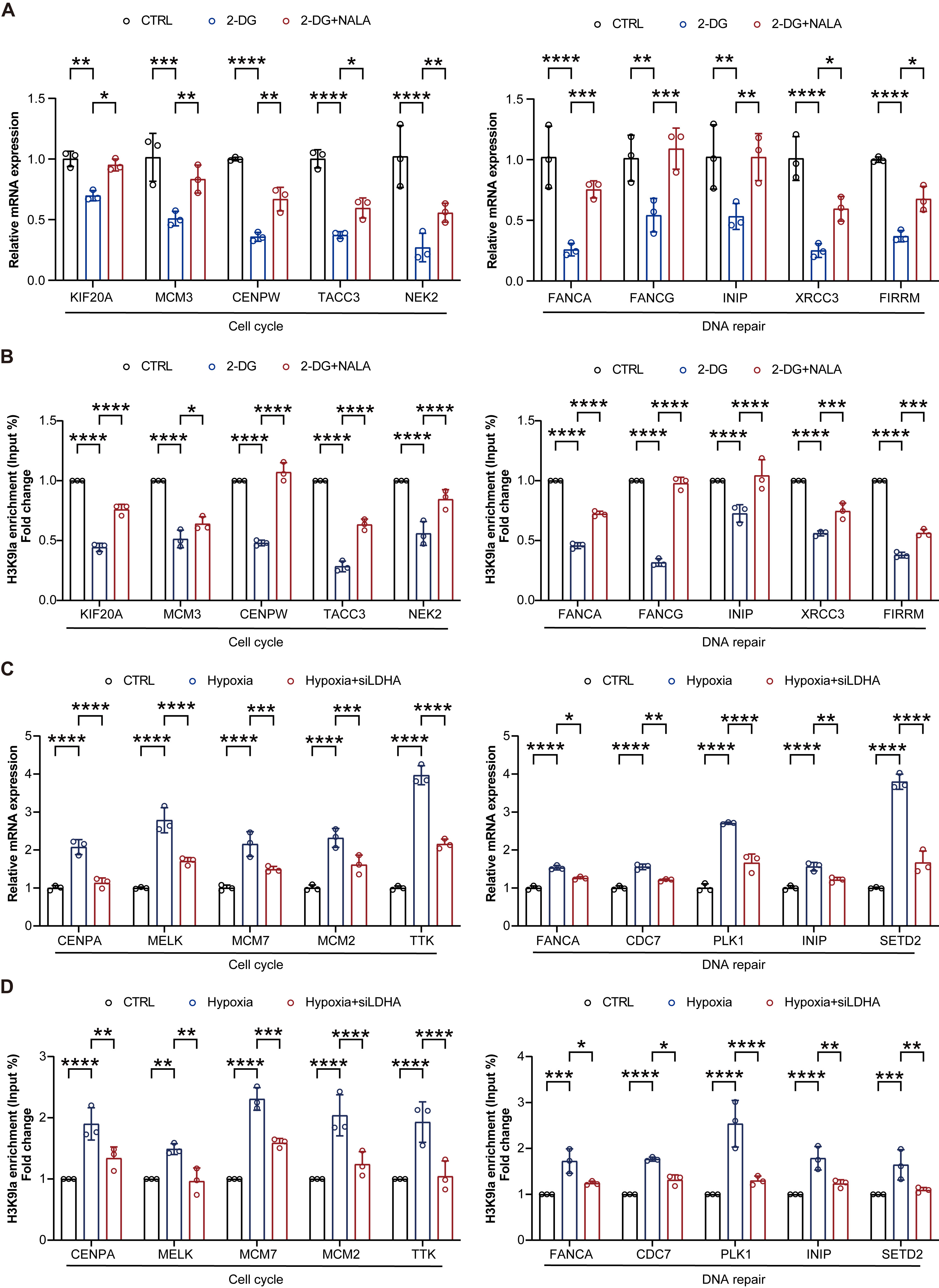


**Fig. S14.** **Gene expression of cell cycle and DNA repair related genes is consistent with histone lactylation.** **A**, **B**, mRNA levels (**A**) and ChIP‒qPCR (**B**) of the cell cycle- and DNA repair-related genes in IMR90 cells cultured with or without 2-DG treatment and 2-DG + NALA. **C**, **D**, mRNA levels (**C**) and ChIP‒qPCR (**D**) of the cell cycle-related and DNA repair-related genes in IMR90 cells cultured under normoxia, hypoxia, and hypoxia + si*LDHA*. The cycle threshold (Ct) values of these genes were normalized to that of *ACTB*. The error bars represent the S.D. of independent experiments, n = 3. One-way ANOVA was performed. **P* < 0.05, ***P* < 0.01, ****P* < 0.001, *****P* < 0.0001.


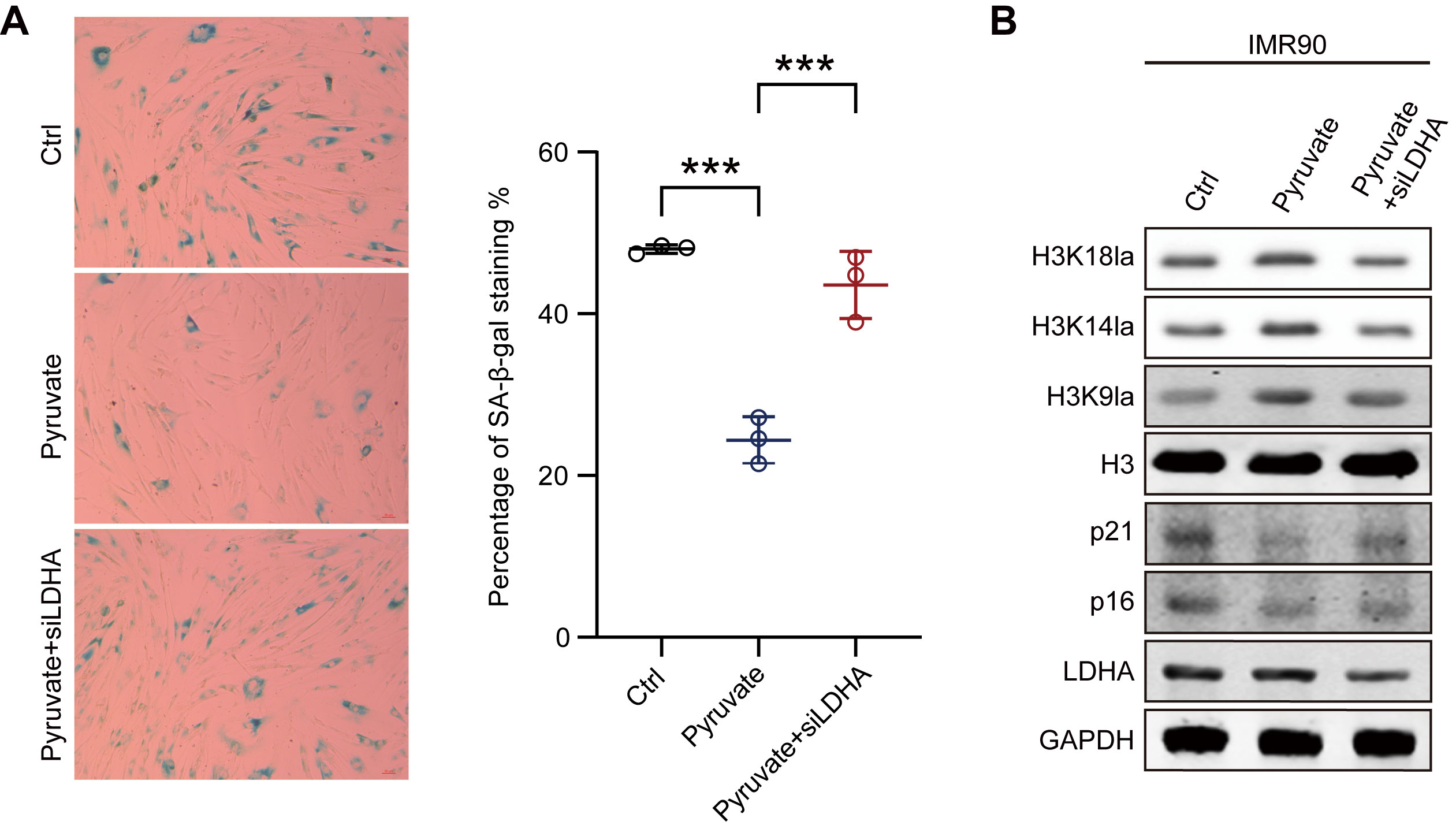


**Fig. S15. Pyruvate increases histone lactylation and delays senescence. A**, SA-β-gal staining of IMR90 cells in the presence or absence of pyruvate and pyruvate + si*LDHA*. The percentages of SA-β-gal^+^ cells are shown on the right. **B**, Immunoblotting of H3K18la, H3K14la, H3K9la, p16, LDHA, and p21 of IMR90 cells in the presence or absence of in the presence or absence of pyruvate and pyruvate + si*LDHA*. H3 and GAPDH served as the loading controls. The error bars represent the S.D. of independent experiments, n = 3. One-way ANOVA was performed. ****P* < 0.001.

**
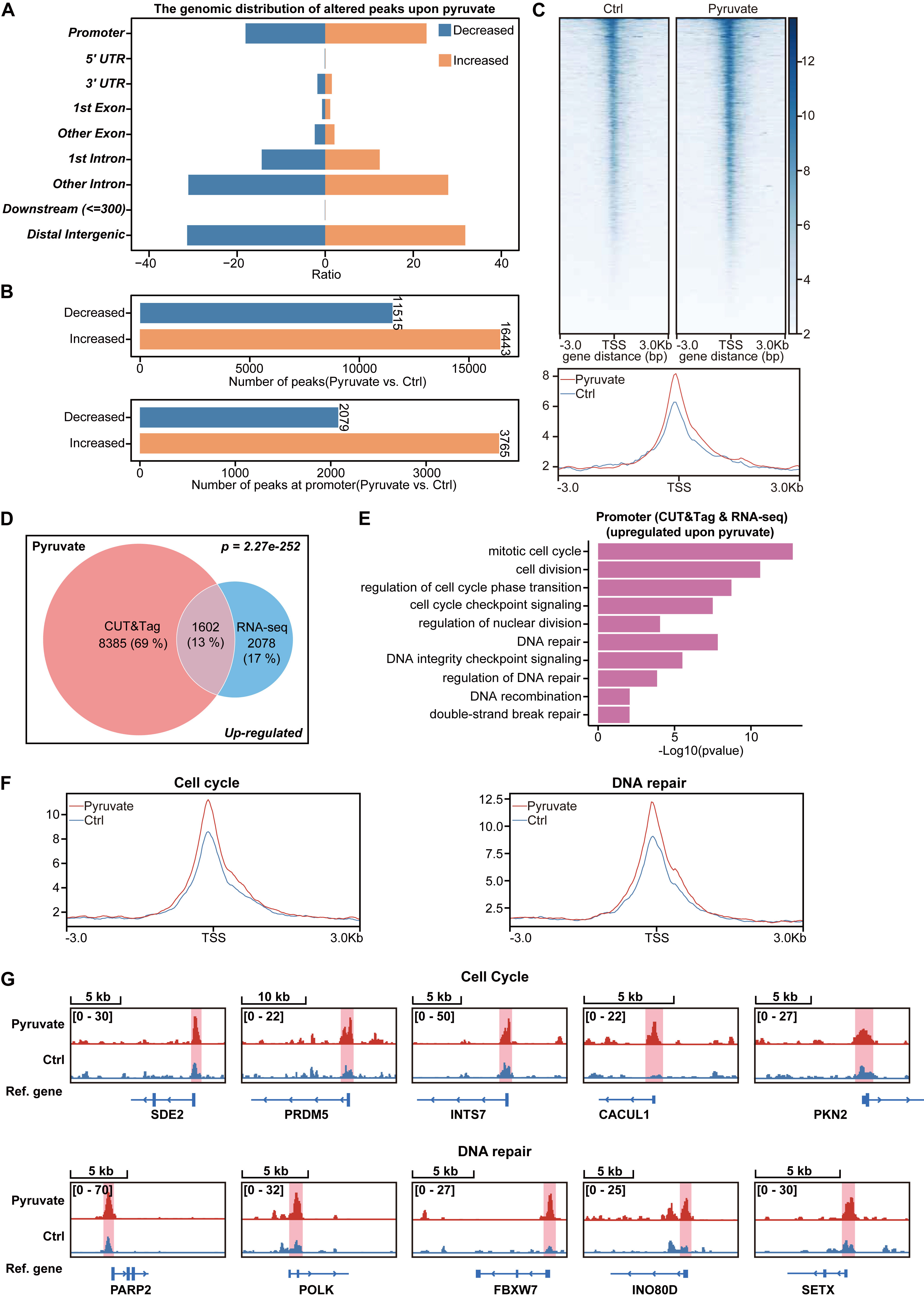
Fig. S16.** **Pyruvate delays senescence by improving cell cycle and DNA repair pathways. A**, Distribution of the altered H3K9la peaks in genomic elements in IMR90 cells with or without pyruvate treatment. **B**, The number of peaks at whole genome and peaks at promoter in IMR90 cells with or without pyruvate treatment. **C**, Heatmaps and intensity profiles of H3K9la around ± 3 kb of TSS throughout the genome in IMR90 cells with or without pyruvate treatment. **D**, Venn diagram showing the overlapping genes between increased H3K9la peak-associated genes identified via CUT&Tag and upregulated genes identified via RNA-seq data related to pyruvate treatment. **E**, Gene Ontology pathway analysis of genes with increased H3K9la peaks at their promoters and corresponding upregulation with pyruvate treatment. **F**, Intensity profiles of H3K9la around ± 3 kb of TSS on cell cycle and DNA repair pathways in IMR90 cells with or without pyruvate treatment. **G**, Snapshots of H3K9la peaks at the promoters of cell cycle- and DNA repair- related genes in IMR90 cells with or without pyruvate treatment. TSS, transcription start site.


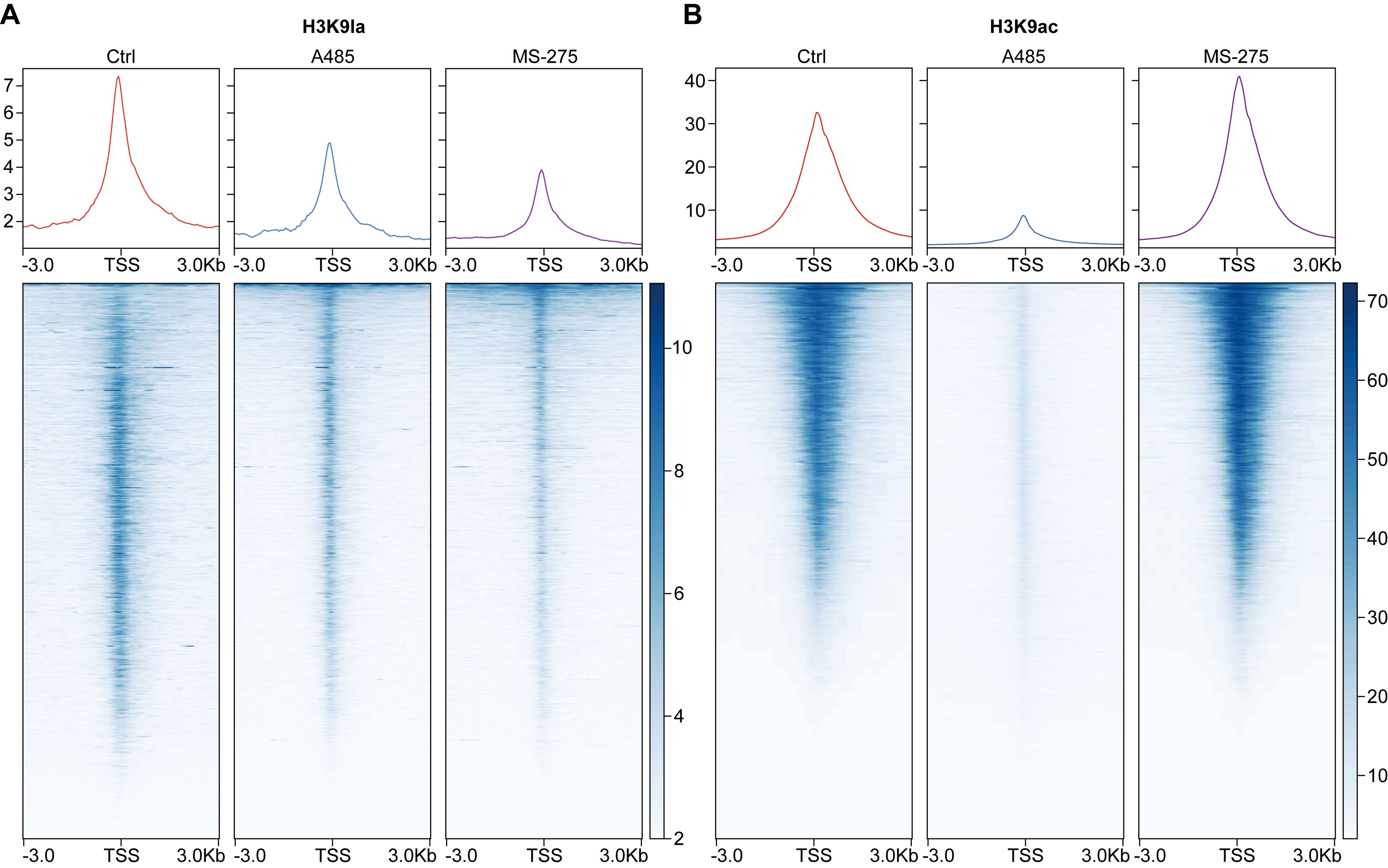


**Fig. S17.** **Histone lactylation and acetylation exhibit different trends after treatment with A485 and MS-275.** **A**, **B**, Heatmaps and intensity profiles of H3K9la (**A**) or H3K9ac (**B**) around ± 3 kb of TSS throughout the genome in IMR90 cells cultured with or without A485 and MS-275 treatment. TSS, transcription start site.

**
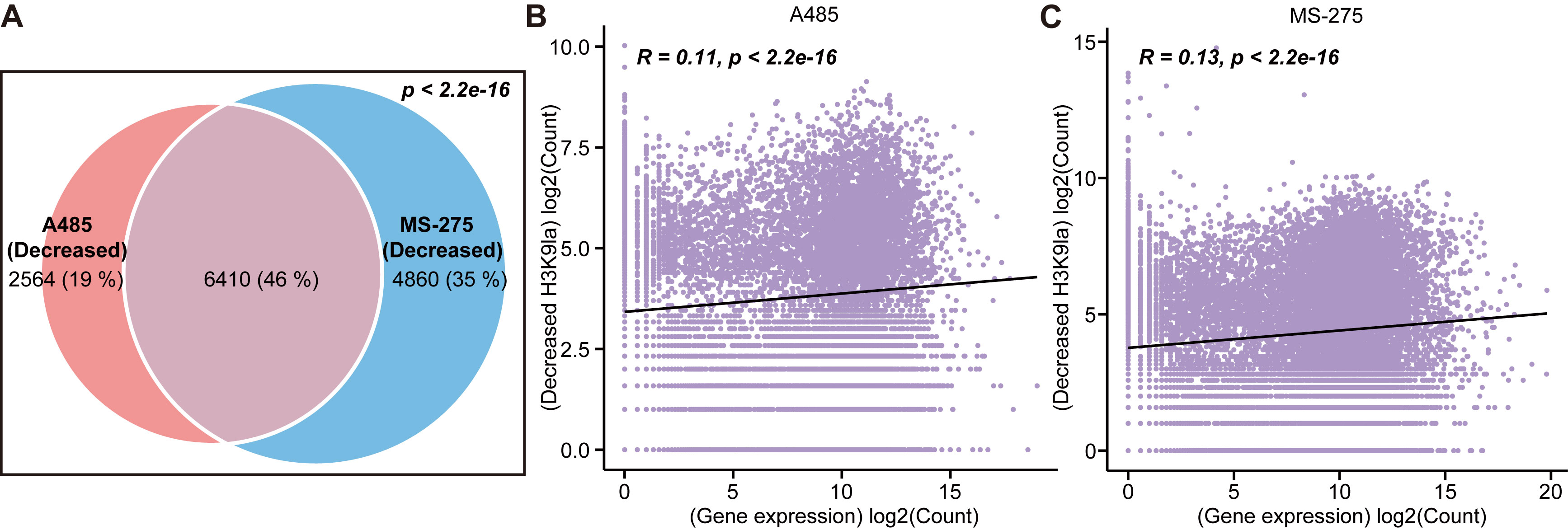
Fig. S18.** **The overlap of genes associated with decreased H3K9la peaks with A485 treatment and decreased H3K9la peaks with MS-275 treatment is significant. A**, The Venn diagram showing the overlap of genes associated decreased H3K9la peaks with A485 treatment and decreased H3K9la peaks with MS-275 treatment. **B**, **C**, Scatter plots showing the correlation between decreased H3K9la peaks in the whole genome (log_2_Count, y-axis) and the corresponding differentially expressed genes expression (log_2_Count, x-axis) for A485 (**B**), and MS-275 (**C**). Pearson’s correlation coefficient R and P value are indicated.


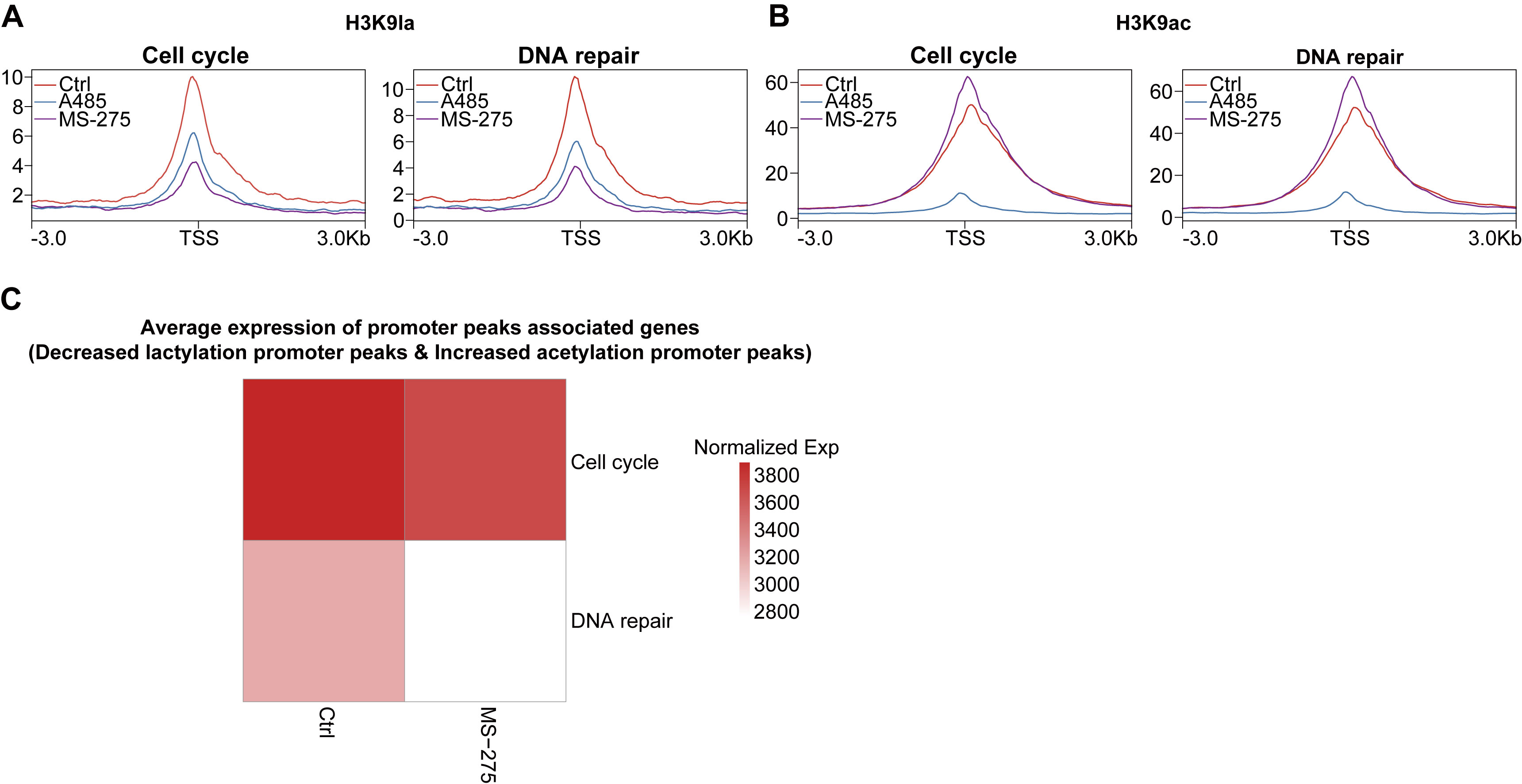


**Fig. S19.** **Decreased histone lactylation downregulates cell cycle and DNA repair pathways. A**, **B**, Intensity profiles of H3K9la (**A**) or H3K9ac (**B**) around ± 3 kb of TSS on cell cycle and DNA repair pathways in IMR90 cells cultured with or without A485 and MS-275 treatment. **C**, Heatmap showing the average expression of cell cycle and DNA repair genes that simultaneously meet the conditions of decreased lactylation promoter peaks and increased acetylation promoter peaks with or without MS-275 treatment. TSS, transcription start site.


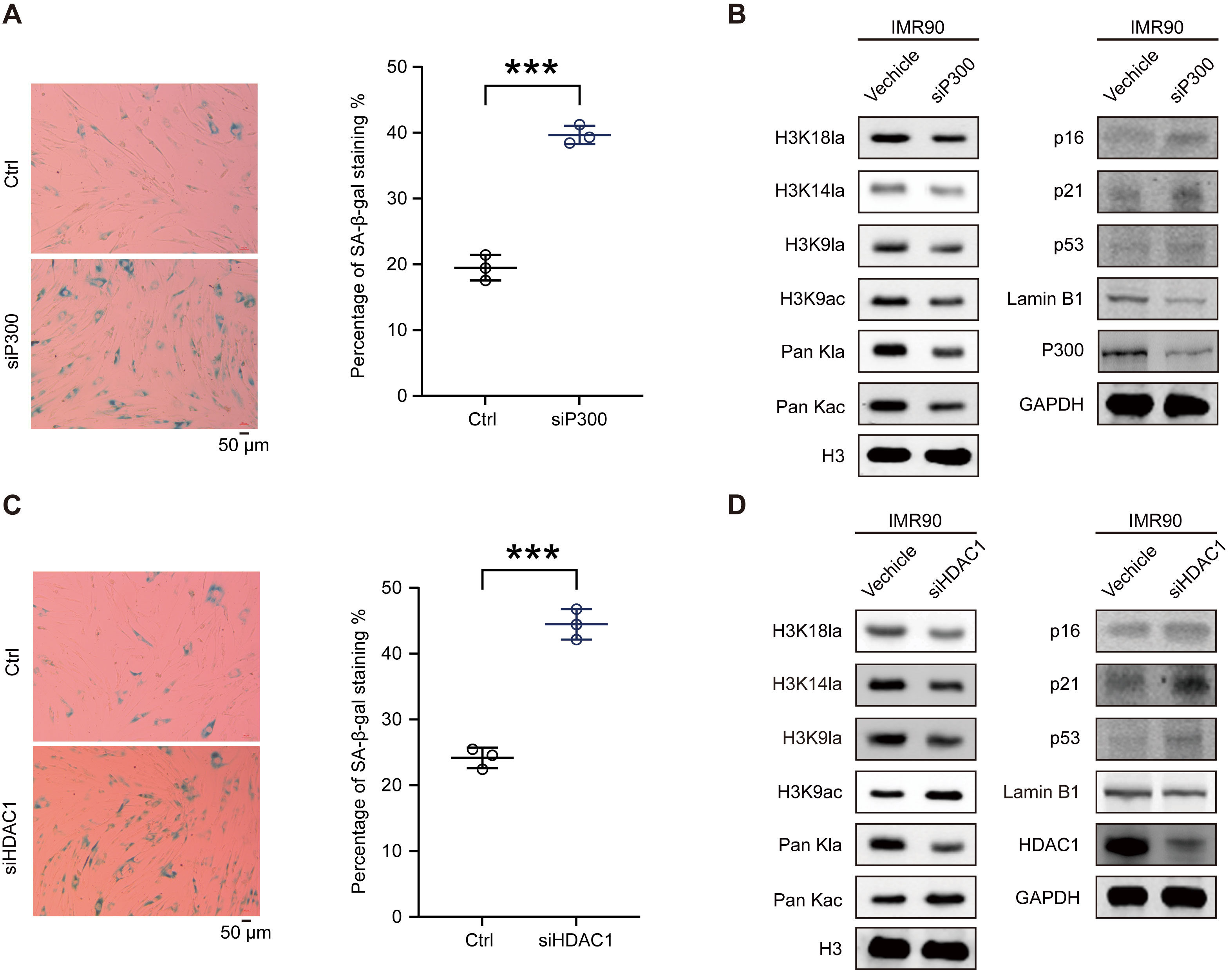
**Fig. S20. Knockdown of *p300* or *Hdac1* decreases histone lactylation and accelerates cellular senescence. A**, SA-β-gal staining of IMR90 cells in the presence or absence of *p300* siRNA. The percentages of SA-β-gal^+^ cells are shown on the right. **B**, Immunoblotting of H3K18la, H3K14la, H3K9la, H3K9ac, Pan Kla, Pan Kac, Lamin B1, p16, p21, and p53 in IMR90 cells in the presence or absence of *p300* siRNA. H3 and GAPDH served as the loading controls. **C**, SA-β-gal staining of IMR90 cells in the presence or absence of *Hdac1* siRNA. The percentages of SA-β-gal^+^ cells are shown on the right. **D**, Immunoblotting of H3K18la, H3K14la, H3K9la, H3K9ac, Pan Kla, Pan Kac, Lamin B1, p16, p21, and p53 in IMR90 cells in the presence or absence of *Hdac1* siRNA. H3 and GAPDH served as the loading controls. The error bars represent the S.D. of independent experiments, n = 3. Two-tailed, unpaired Student’s *t* tests were performed. ****P* < 0.001.

**
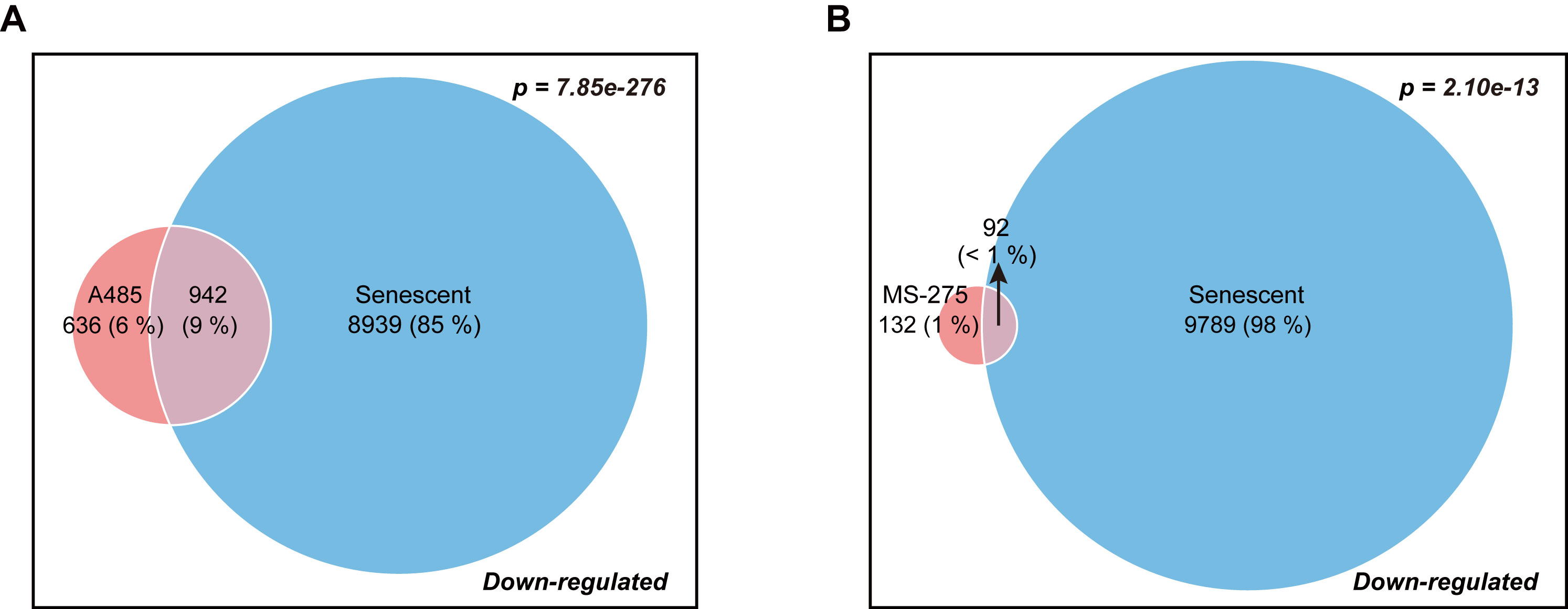
Fig. S21.** **A485 and MS-275 accelerate senescence by regulating senescence-associated genes. A**, Venn diagram showing the overlapping genes between genes downregulated upon A485 treatment and genes downregulated during senescence. **B**, Venn diagram showing the overlapping genes between genes downregulated upon MS-275 treatment and genes downregulated during cellular senescence.


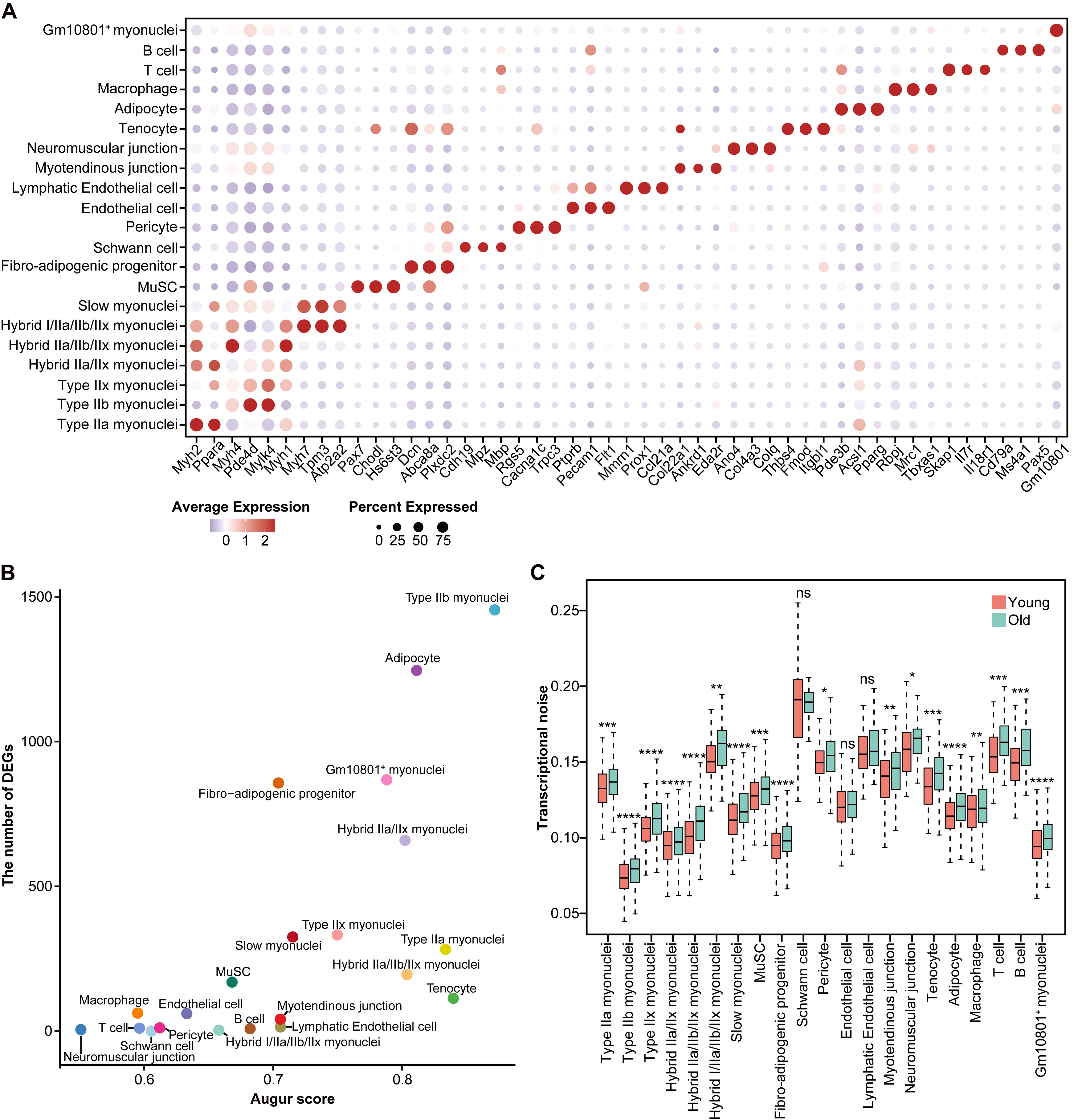
**Fig. S22. Identification and characterizing of cell populations in young and old skelatal muscle using single-nucleus RNA sequencing. A**, Dot plot of marker gene expression of the 21 major cell types in young and old muscle. The x-axis represents the marker genes, and the y-axis represents the cell types. **B**, Dotplot showing the transcriptional alterations during aging. The x-axis represents the sensitivity of cell types to aging, and the y-axis represents the number of DEGs. **C**, Boxplot showing the levels of transcriptional noise in 21 cell types along with skeletal muscle aging. **P* < 0.05, ***P* < 0.01, ****P* < 0.001, *****P* < 0.0001, ns, not significant.

**
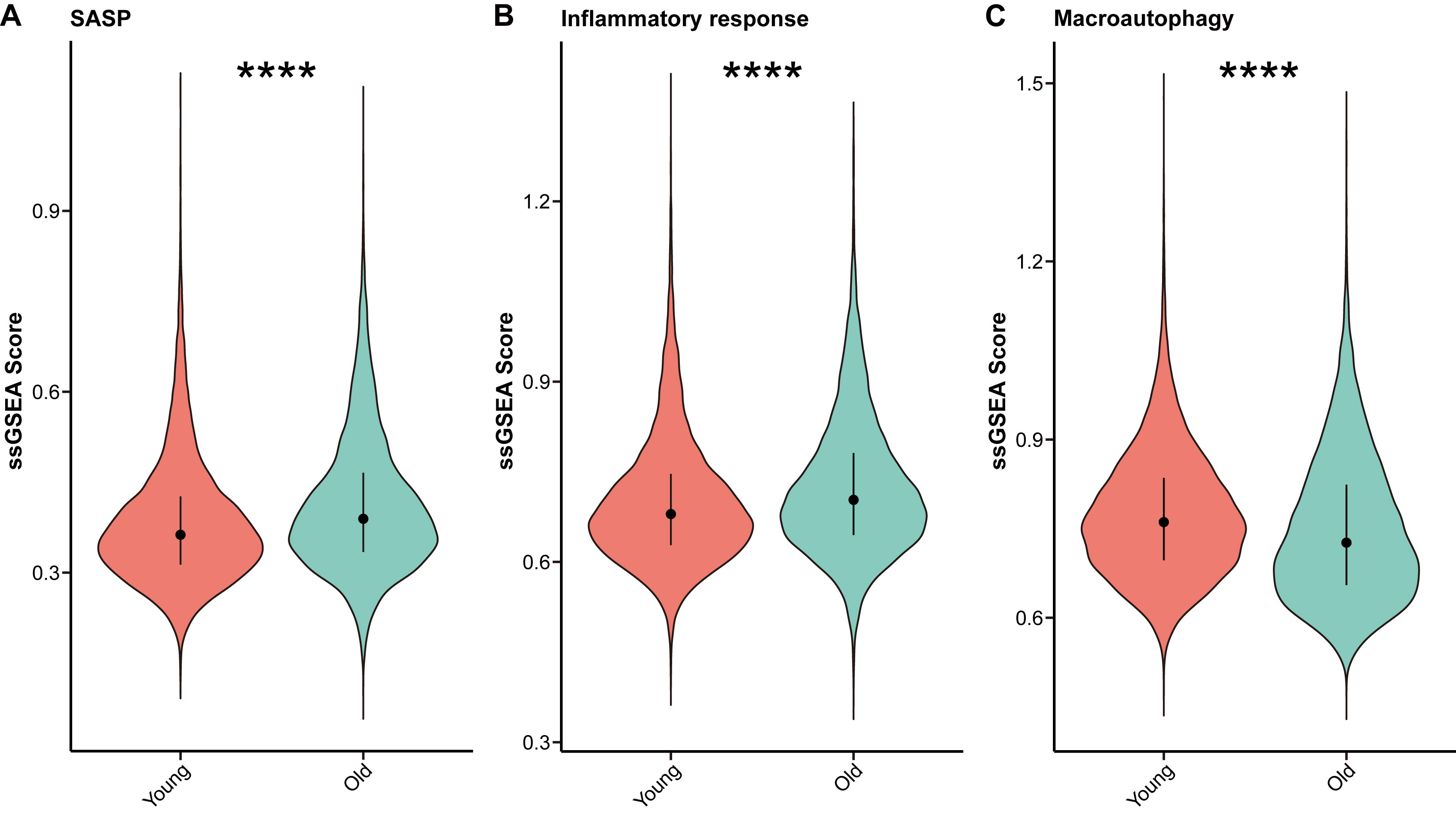
Fig. S23.** **The aging-related phenotype changes during muscle aging.** **A**-**C**, Alterations in the expression of SASP (**A**), inflammatory response (**B**), and macroautophagy (**C**) pathways in muscle of young and old mice. *****P* < 0.0001.

**
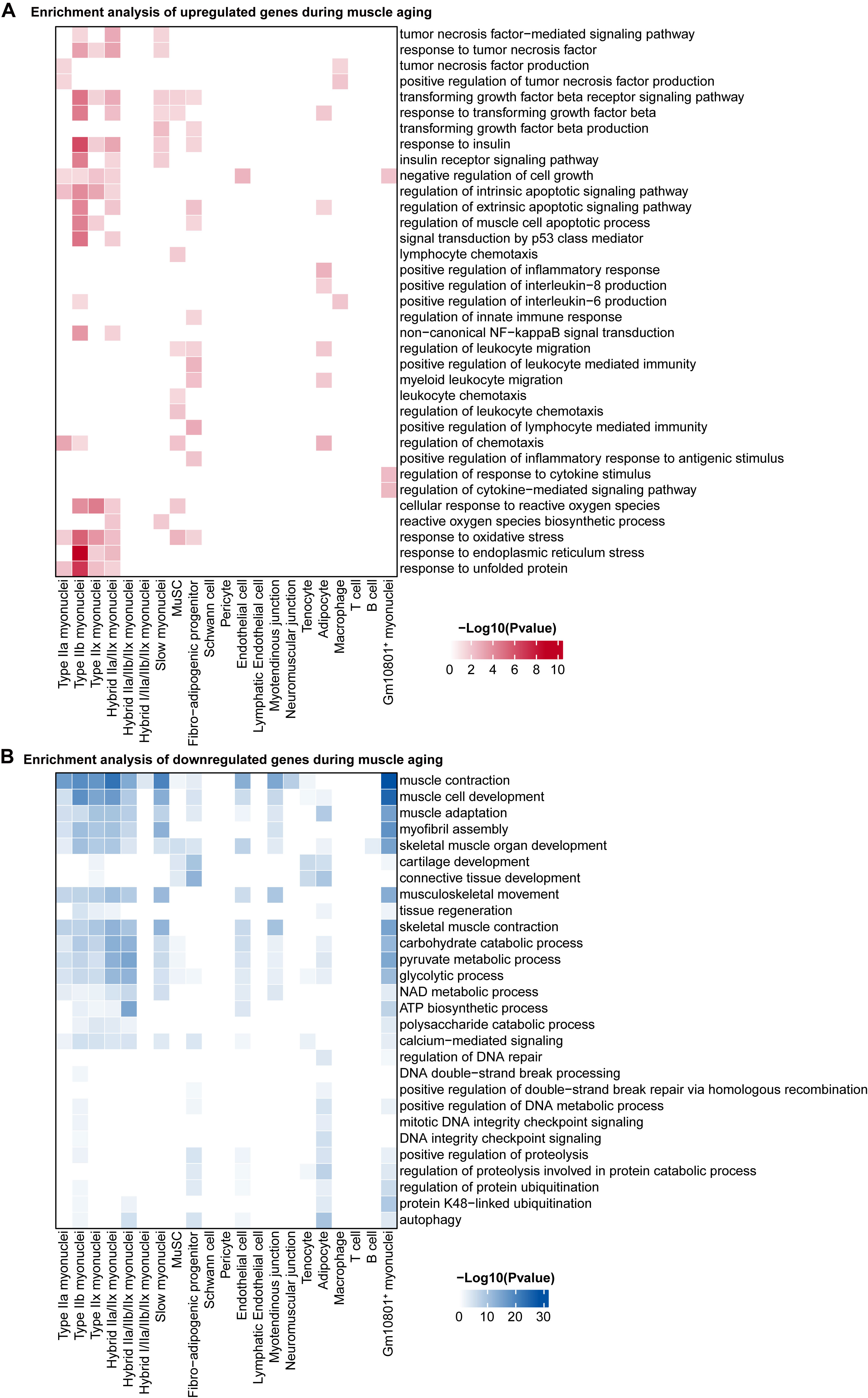
Fig. S24.** **Enrichment analysis of upregulated and downregulated genes in each cell type during muscle aging.** **A**, **B**, Gene Ontology pathway analysis of upregulated genes (**A**) and downregulated genes (**B**) during muscle aging.

**
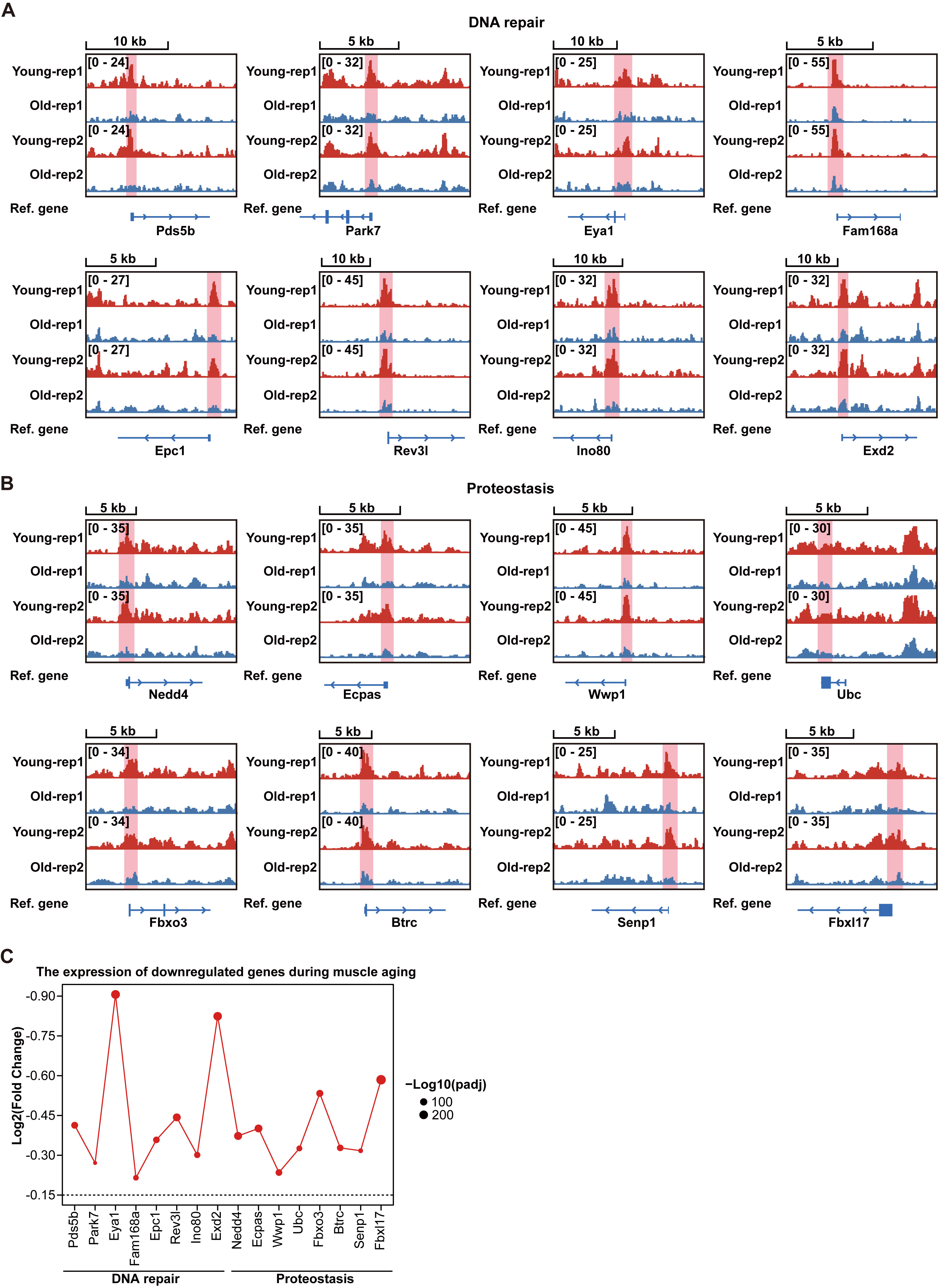
**

**Fig. S25.** **Histone lactylation and gene expression of DNA repair and proteostasis genes decrease during muscle aging. A**, **B**, Snapshots of H3K9la peaks at the promoters of DNA repair- (**A**) and proteostasis- (**B**) related genes in muscle of young and old mice. **C**, mRNA levels of DNA repair- and proteostasis- related genes in muscle of young and old mice.


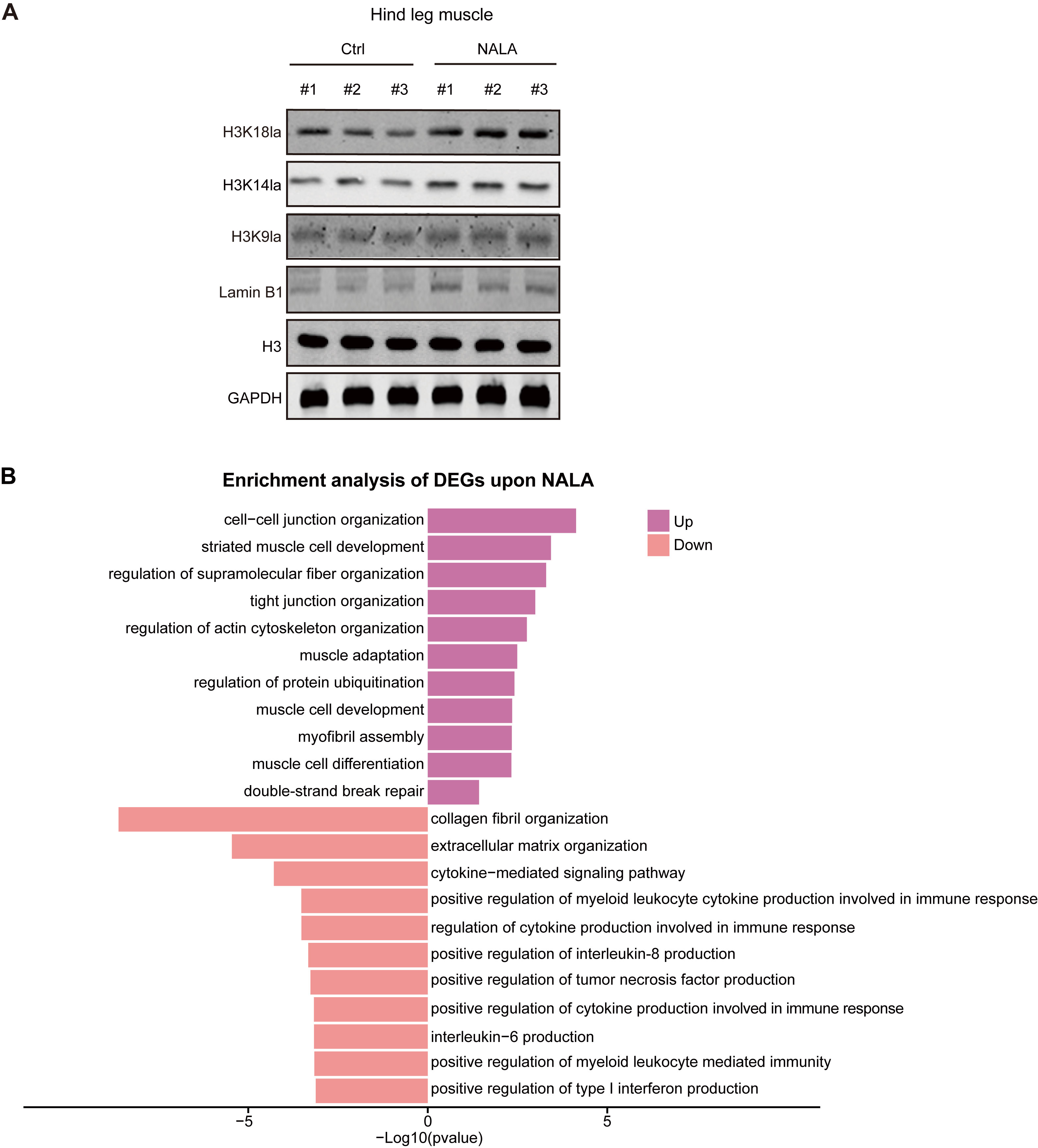
**Fig. S26. Sodium lactate injection restores muscle function during muscle aging.** **A**, Immunoblotting of H3K18la, H3K14la, H3K9la, and Lamin B1 in muscle with or without NALA treatment. H3 and GAPDH served as the loading controls. **B**, Gene Ontology pathway analysis of upregulated and downregulated genes in muscle with NALA treatment.

**
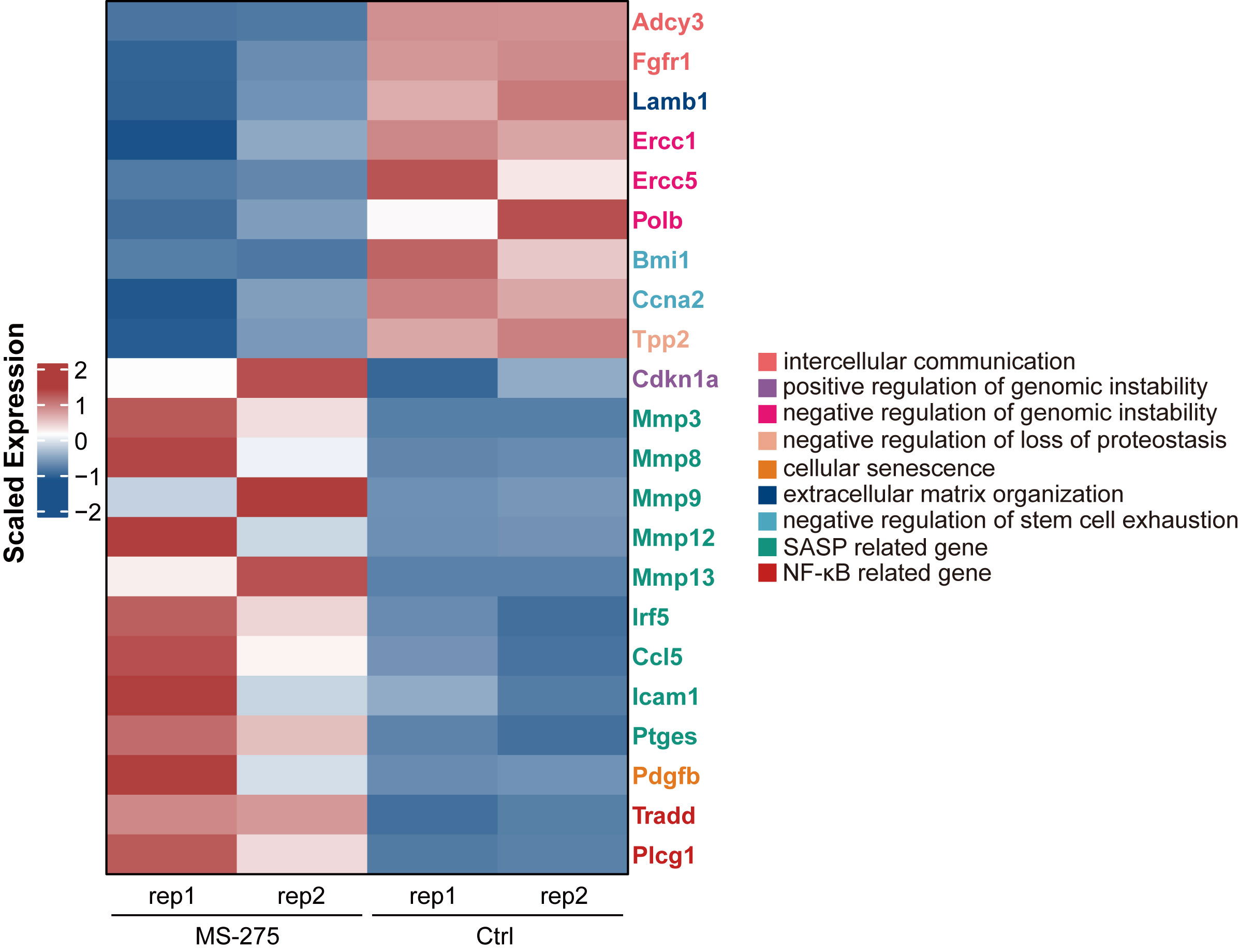
Fig. S27. Injection of MS-275 accelerates muscle aging.** Heatmap of aging-associated gene alterations in the gastrocnemius muscle with MS-275 treatment.

**
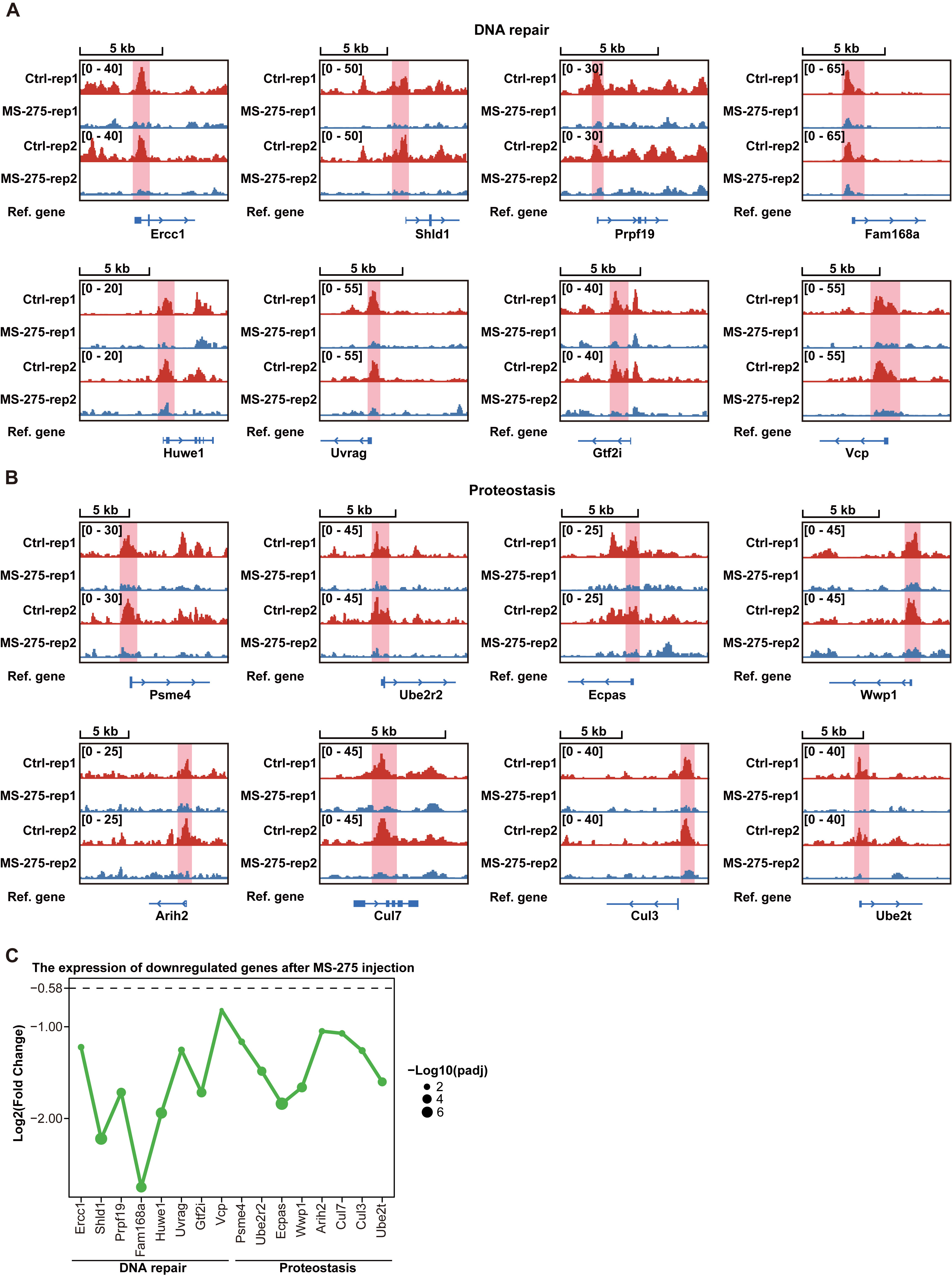
Fig. S28. Histone lactylation and gene expression of DNA repair and proteostasis genes decrease after MS-275 injection.** **A**, **B**, Snapshots of H3K9la peaks at the promoters of DNA repair- (**A**) and proteostasis- (**B**) related genes in muscle with or without MS-275 treatment. **C**, mRNA levels of DNA repair- and proteostasis- related genes in muscle with or without MS-275 treatment.

**
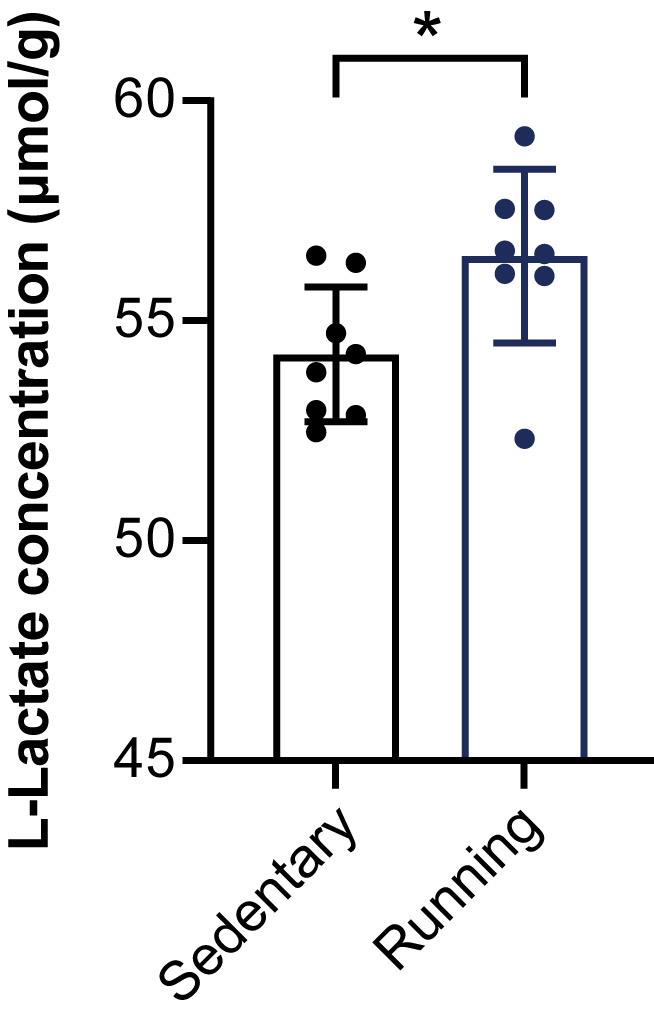
Fig. S29.** **Lactate increases in muscle of running mice compare to sedentary mice.** The L-Lactate concentration in muscle of sedentary and running mice. n=8. The error bars represent the S.D. of independent experiments. Two-tailed, unpaired Student’s *t* tests were performed. **P* < 0.05.


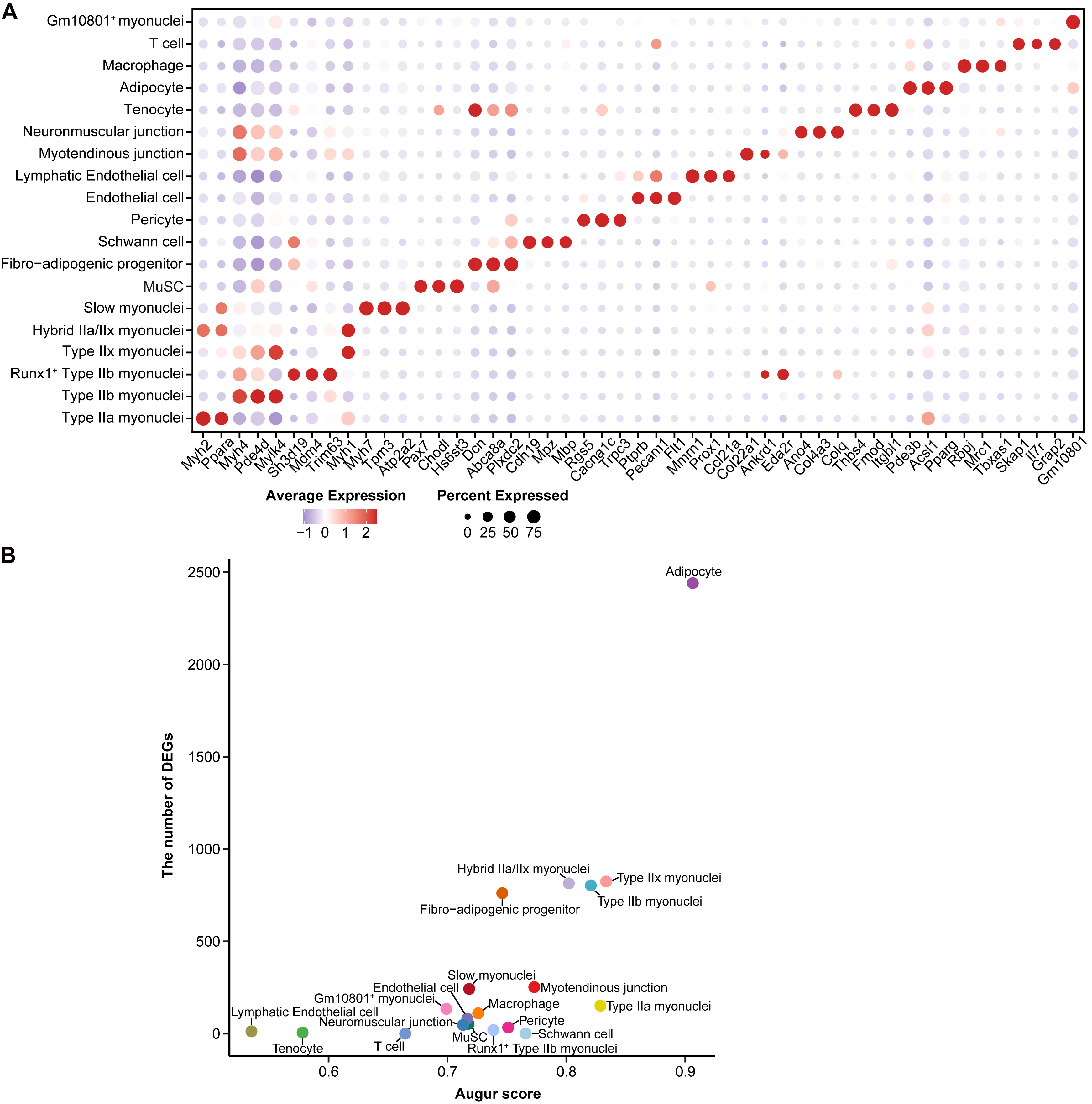


**Fig. S30. Identification and characterizing of cell populations in sedentary and running skelatal muscle using single-nucleus RNA sequencing. A**, Dot plot of marker gene expression of the 19 major cell types in sedentary and running muscle. The x-axis represents the marker genes, and the y-axis represents the cell types. **B**, Dotplot showing the transcriptional alterations after running exercise. The x-axis represents the sensitivity of cell types to running exercise, **
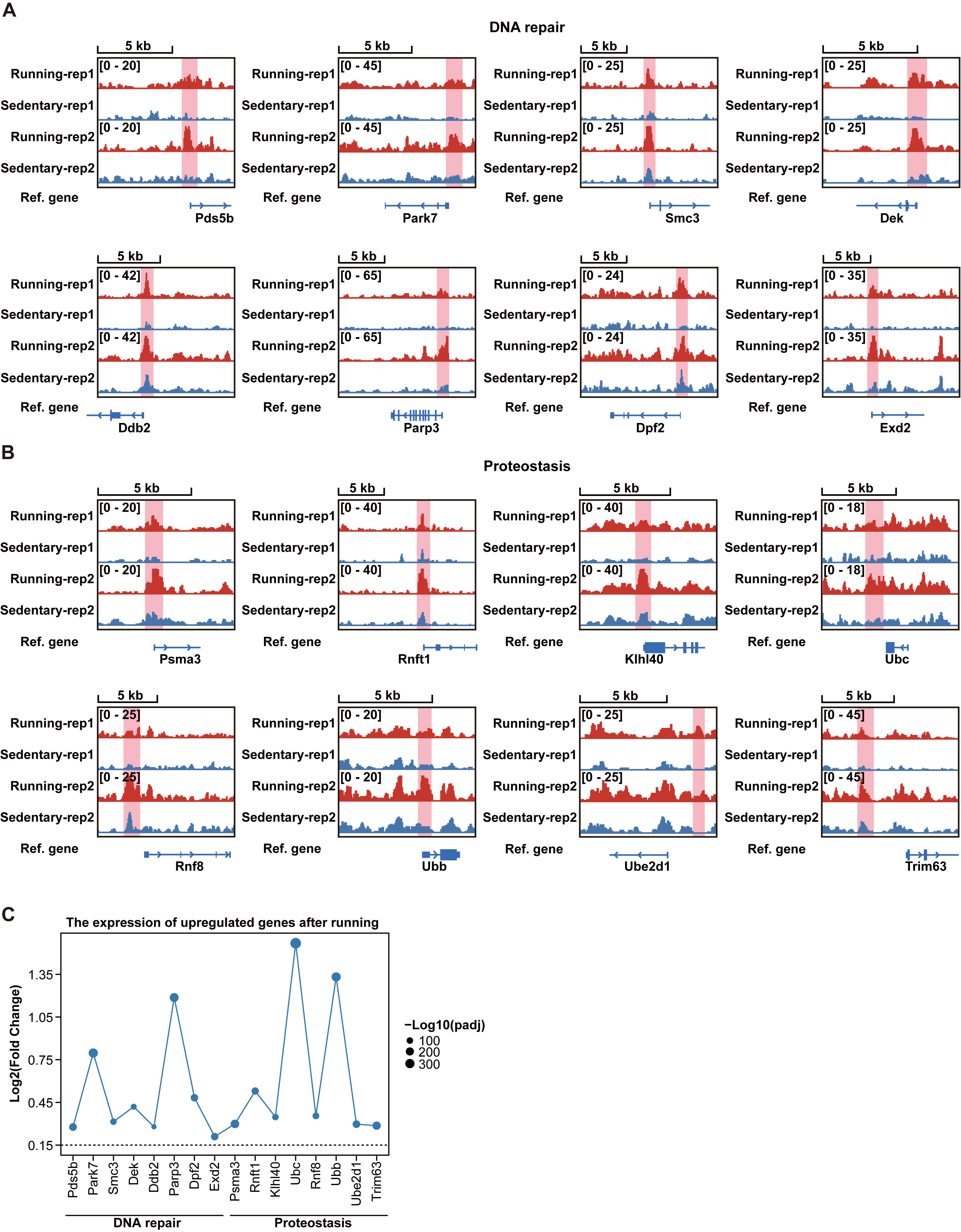
**and the y-axis represents the number of DEGs.

**Fig.** **S31. Histone lactylation and gene expression of DNA repair and proteostasis genes increase after running.** **A**, **B**. Snapshots of H3K9la peaks at the promoters of DNA repair (**A**) and proteostasis (**B**) related genes in muscle of sedentary and running mice. **C**, mRNA levels of DNA repair and proteostasis related genes in muscle of sedentary and running mice.

**Table S1. Primers used for RT‒qPCR**

| **Gene** | **Primer sequence** |
| --- | --- |
| ***KIF11*** | For: 5’- AGCAAGCTGCTTAACACAGTT -3’ |
|  | Rev: 5’- CCTTCTTACGATCCAGTTTGGAA -3’ |
| ***KAT6A*** | For: 5’- TCACCAGCAGTTACGATTGGC -3’ |
|  | Rev: 5’- CATCCACGTTGGTTGCTTTAGT -3’ |
| ***CDT1*** | For: 5’- CGGTGGACGAGGTTTCCAG -3’ |
|  | Rev: 5’- CTGCCGGGGTGGATTTCTT -3’ |
| ***CENPK*** | For: 5’- AGTACCTTGGGCGAGTTTCTA -3’ |
|  | Rev: 5’- AGGCAATTCCATTACGCAGCA -3’ |
| ***ACTB*** | For: 5’-CATGTACGTTGCTATCCAGGC-3’ |
|  | Rev: 5’-CTCCTTAATGTCACGCACGAT-3’ |
| ***NDRG1*** | For: 5’- CCAACAAAGACCACTCTCCTC -3’ |
|  | Rev: 5’- CCATGCCCTGCACGAAGTA -3’ |
| ***NSD2*** | For: 5’- ACCGCGAGTGTTCTGTGTTC -3’ |
|  | Rev: 5’- GTCGTGGCCGTTAAACTTCTG -3’ |
| ***LIG1*** | For: 5’- GAAGGAGGCATCCAATAGCAG -3’ |
|  | Rev: 5’- ACTCTCGGACACCACTCCATT -3’ |
| ***FANCC*** | For: 5’- TCAAGGTCTTGGGTATGCACC -3’ |
|  | Rev: 5’- GCCATTCGCCTTTGAGTGTTAAA -3’ |
| ***KIF20A*** | For: 5’- TGCTGTCCGATGACGATGTC -3’ |
|  | Rev: 5’- AGGTTCTTGCGTACCACAGAC -3’ |
| ***MCM3*** | For: 5’- TCAGAGAGATTACCTGGACTTCC -3’ |
|  | Rev: 5’- TCAGCCGGTATTGGTTGTCAC -3’ |
| ***CENPW*** | For: 5’- T AAGCCTCAACTTCGTCTGGAG -3’ |
|  | Rev: 5’- CACAAGCGTTTGTCCTGGACT -3’ |
| ***TACC3*** | For: 5’- TCGCCACCAGAAGTTACCG -3’ |
|  | Rev: 5’- TCCCGCAGAGGTGTCTGAAA -3’ |
| ***NEK2*** | For: 5’- TGCTTCGTGAACTGAAACATCC -3’ |
|  | Rev: 5’- CCAGAGTCAACTGAGTCATCACT -3’ |
| ***FANCA*** | For: 5’- GGCACACAGTATGTTCTCCCG -3’ |
|  | Rev: 5’- TTGTACGTGAAGATGCCACAC -3’ |
| ***FANCG*** | For: 5’- CAGGGATTGAAGGATGTCCTCC -3’ |
|  | Rev: 5’- TGGATTTCCCATCTTACGGTGA -3’ |
| ***INIP*** | For: 5’- AGCATATTGCAGCCCAACAGA -3’ |
|  | Rev: 5’- TGCAGAGTCTTGAGTGATGAAGT -3’ |
| ***XRCC3*** | For: 5’- CCCCATTCCGCTGTGAATTTG -3’ |
|  | Rev: 5’- GGTTAGCCCAGGTTATGCCA -3’ |
| ***FIRRM*** | For: 5’- AGCCTTTATGCTACCAGGATTTC -3’ |
|  | Rev: 5’- GGCAGGTCTAATTTACTGTCCAA -3’ |
| ***CENPA*** | For: 5’- GACGCCTATCTCCTCACCTTA -3’ |
|  | Rev: 5’- GTTGCACATCCTTTGGGAAGA -3’ |
| ***MELK*** | For: 5’- TATTCACCTCGATGATGATTGCG -3’ |
|  | Rev: 5’- AGAAAGCCTTAAACGAACTGGTT -3’ |
| ***MCM2*** | For: 5’- CCGTGACCTTCCACCATTTGA -3’ |
|  | Rev: 5’- GGTAGTCCCTTTCCATGCCAT -3’ |
| ***MCM7*** | For: 5’- ACTCTCAGAAACCTACCTGGAAG -3’ |
|  | Rev: 5’- CAGCTTTTCGTAGAAATCCTCCT -3’ |
| ***TTK*** | For: 5’- TCATGCCCATTTGGAAGAGTC -3’ |
|  | Rev: 5’- CCACTTGGTTTAGATCCAGGC -3’ |
| ***CDC7*** | For: 5’- GAGGCGTCTTTGGGGATTCAG -3’ |
|  | Rev: 5’- GGTCCTACTTGTAACTGTGCTG -3’ |
| ***PLK1*** | For: 5’- AAAGAGATCCCGGAGGTCCTA -3’ |
|  | Rev: 5’- GGCTGCGGTGAATGGATATTTC -3’ |
| ***SETD2*** | For: 5’- TGCTTCTAGTCGATTTTTGCCC -3’ |
|  | Rev: 5’- AGGGTTTGGAGTATCACTTTGC -3’ |
| ***SMC4*** | For: 5’- GGCTGTATGGGCGAAAAAGAT -3’ |
|  | Rev: 5’- TTGTGGCTTGATCCAAGTTGT -3’ |
| ***PCLAF*** | For: 5’- ATGGTGCGGACTAAAGCAGAC -3’ |
|  | Rev: 5’- CCTCGATGAAACTGATGTCGAAT -3’ |
| ***NCAPG2*** | For: 5’- TACAAGCCGTGTCTAAGGAGC -3’ |
|  | Rev: 5’- TTGAGCCATGTTCGGTTTCCA -3’ |
| ***HJURP*** | For: 5’- CACAAAGCCATCAAGCATCATC -3’ |
|  | Rev: 5’- TCAGAGCAGGGTATGAAGTTCT -3’ |
| ***POLQ*** | For: 5’- ACCTCTCCATCAAGGCATTTCT -3’ |
|  | Rev: 5’- GCAAAAGTTCCAGCAGATACCC -3’ |
| ***MCM4*** | For: 5’- TGAACCTCTATACATGCAACGAC -3’ |
|  | Rev: 5’- CAGGGTAACGGTCAAAGAAGATT -3’ |
| ***RAD54L*** | For: 5’- AGGCAGGTCCTGTGATGATGA -3’ |
|  | Rev: 5’- TCAAAGGTTTCCGAAAAGGAGAC-3’ |
| ***UHRF1*** | For: 5’- AGGTGGTCATGCTCAACTACA -3’ |
|  | Rev: 5’- CACGTTGGCGTAGAGTTCCC -3’ |

**Table S2. Primers used for** **ChIP‒qPCR**

| **Name** | **Primer sequence** |
| --- | --- |
| ***KIF20A*** | For: 5’ - TTTGAGGTGCCGGTTACTCC -3’ |
|  | Rev: 5’- AGCATCGTGAAGACCAACGT -3’ |
| ***MCM3*** | For: 5’- GCCTAGAAGCCTCAGAGCAG -3’ |
|  | Rev: 5’- TCACTGATGCGAGCGAGATC -3’ |
| ***CENPW*** | For: 5’- GGGTGTGGTGAGAGGAACTG -3’ |
|  | Rev: 5’- TCATACCCTAGGCGGTGTCA -3’ |
| ***TACC3*** | For: 5’- TTGACATCCGACTGTGAGGC -3’ |
|  | Rev: 5’- AGAGATGGATGGGGCTGAGT -3’ |
| ***NEK2*** | For: 5’- CTTGCAGCAGTTCCACACAC-3’ |
|  | Rev: 5’- CTATCGCCCAGCTCAAGGAG-3’ |
| ***FANCA*** | For: 5’- GGTCACAACCAGCAGTTCCT -3’ |
|  | Rev: 5’- ATCCTGGCCTCAAGCAATCC -3’ |
| ***FANCG*** | For: 5’- TACGCTTCCTCGACGGATTG -3’ |
|  | Rev: 5’- ACTCCGGAGCCAATGAACTG -3’ |
| ***INIP*** | For: 5’- CTCTCTGTGCTCCAGCCAAA -3’ |
|  | Rev: 5’- CTGAGATCGAGCCACTGGAC -3’ |
| ***XRCC3*** | For: 5’- CTGAGTACCTGAGTCCCCGA -3’ |
|  | Rev: 5’- GGGTTCCTCTCACCAGAACG -3’ |
| ***FIRRM*** | For: 5’- GGTCCTCTTTCCAAGCAGCT -3’ |
|  | Rev: 5’- AAGCAGCTGTATGGTGGGAC -3’ |
| ***CENPA*** | For: 5’- TAGGTCTACTGGCCCTGTCC -3’ |
|  | Rev: 5’- TGGCACAGCTGCTATGGAAA -3’ |
| ***MELK*** | For: 5’- GTGGTTCCGAACAACTTGGC -3’ |
|  | Rev: 5’- TTCTGACACCTGCTGCAACA -3’ |
| ***MCM2*** | For: 5’- CCTGTGCGCTCCTATCTCAG -3’ |
|  | Rev: 5’- GCTGGTCTCCAACTCCTGAC -3’ |
| ***MCM7*** | For: 5’- GCCACAATGCTGTTTGCTGA -3’ |
|  | Rev: 5’- GGATGAGGACAGGAGGAGGT -3’ |
| ***TTK*** | For: 5’- ACACAAAACCAACCCGTCCT -3’ |
|  | Rev: 5’- CCCACTTAAGGGCTCAGGTG -3’ |
| ***CDC7*** | For: 5’- GAGAATGCACACCATGGGGA -3’ |
|  | Rev: 5’- TCCAATCCAGGCCAAAGTCC -3’ |
| ***PLK1*** | For: 5’- AGCAGCTCCCAATGAGAACC-3’ |
|  | Rev: 5’- CAGGTGTAAGCCTCCCACAG -3’ |
| ***SETD2*** | For: 5’- AGACCAGCCTGACCAACATG -3’ |
|  | Rev: 5’- CCTCCCAGGTTCAAGCGATT -3’ |
